# The Hierarchical Modular Structure of HER2+ Breast Cancer Network

**DOI:** 10.3389/fphys.2018.01423

**Published:** 2018-10-11

**Authors:** Sergio Antonio Alcalá-Corona, Jesús Espinal-Enríquez, Guillermo de Anda-Jáuregui, Enrique Hernández-Lemus

**Affiliations:** ^1^Computational Genomics, National Institute of Genomic Medicine, Mexico City, Mexico; ^2^Centro de Ciencias de la Complejidad, Universidad Nacional Autónoma de Mexico, Ciudad de Mexico, Mexico

**Keywords:** modular networks, breast cancer, gene regulatory networks, transcription, genetic, signaling pathways, molecular subtypes

## Abstract

HER2-enriched breast cancer is a complex disease characterized by the overexpression of the ERBB2 amplicon. While the effects of this genomic aberration on the pathology have been studied, genome-wide deregulation patterns in this subtype of cancer are also observed. A novel approach to the study of this malignant neoplasy is the use of transcriptional networks. These networks generally exhibit modular structures, which in turn may be associated to biological processes. This modular regulation of biological functions may also exhibit a hierarchical structure, with deeper levels of modular organization accounting for more specific functional regulation. In this work, we identified the most probable (maximum likelihood) model of the hierarchical modular structure of the HER2-enriched transcriptional network as reconstructed from gene expression data, and analyzed the statistical associations of modules and submodules to biological functions. We found modular structures, independent from direct ERBB2 amplicon regulation, involved in different biological functions such as signaling, immunity, and cellular morphology. Higher resolution submodules were identified in more specific functions, such as micro-RNA regulation and the activation of viral-like immune response. We propose the approach presented here as one that may help to unveil mechanisms involved in the development of the pathology.

## 1. Introduction

Breast cancer is the malignant neoplasy with the highest incidence and mortality among women worldwide (Ferlay et al., 2014). Breast cancer is a heterogeneous disease, and this poses a challenge for its treatment: a multitude of clinical, physiological and survival outcomes, all affect the choice of therapeutic options (Polyak, 2011; Network, 2012). The heterogeneity of breast cancer can be traced down to the subcellular level, which includes changes in the transcriptional programs through mutations, alterations of epigenetic regulation, chromosomal aberrations, among others.

HER2-enriched (HER2+) breast cancer is a paradigmatic example of the role of alterations in the chromosomal structure throughout the development of cancer. HER2+ breast cancer is characterized by the overexpression of the *HER2* receptor, encoded in the *ERBB2* gene located on Chromosome 17: Amplification of the Chr17q12 locus leads to the overexpression of the receptor that can be identified through immunohistochemical and transcriptomic approaches (Perou et al., 2000; Burstein, 2005). This *HER2 amplicon* also includes genes such as STARD3, GRB7, PGAP3, TOP2, MED1, THRA, RARA, IGFPB4, CCR7, KRT20, KRT19, and GAST.

While the effects that genes in this region have in the HER2+ breast cancer phenotype have been extensively explored, high-throughput technologies that allow genome-wide studies open the possibility for further exploration of the genomic landscape of this pathology. Major breakthroughs in the study of genomic cancer landscapes have been accomplished through the use of network theory. For instance, relationships between gene expression levels can be modeled as transcriptional networks. These networks have proven to be useful to associate different biological features of interest to a particular phenotype (de Anda-Jáuregui et al., 2016; Espinal-Enríquez et al., 2017).

A major topological feature of transcriptional networks is the fact that these have *modular* structures. Modules in networks, also known in some contexts as *communities*, are structural sub-units (subnetworks) broadly defined as subsets of tightly interconnected nodes so that the density of *within-connections* is higher than that of *between-connections* (Girvan and Newman, 2002; Porter et al., 2009; Fortunato, 2010). One challenge of the community structure finding algorithms is that there is no consensus in the appropriate within/between ratio. One measure of the efficacy of different algorithms with variable ratios is given by the LFR benchmark (Lancichinetti et al., 2009). In some cases, modules in transcriptional networks are associated to biological processes and functions observed in a given phenotype (Cantini et al., 2015). Thus, modules in a gene transcriptional network may capture some functional aspects underlying the phenomenology.

In this work, we explored the modular structure in the HER2+ breast cancer transcriptional network. We previously inferred said network (de Anda-Jáuregui et al., 2015), using a Mutual Information (MI)- based algorithm, from gene expression patterns measured using microarrays. Then, we analyzed the modularity of this network at three different levels: connected components (islands), modules in the largest component, and submodules in the largest modules. Over-representation analysis (ORA) was used to identify modular structures associated with functional categories as defined in Gene Ontology (GO).

Our analysis identified a transcriptional network with an explicit modular structure at each level of modularity resolution explored. We found associations between some of these modules and biological features of interest. The highest level of modular organization, that of connected components, captures the transcriptional relationships associated with the HER2 amplicon. Modules inside connected components were identified linked to biological processes, including Extracellular Matrix (ECM) organization, signaling, and immune response. Finally, at the finest level of modular organization, we found groups of genes associated to more specific processes, such as viral response, plasma membrane organization, and micro-RNA regulation. These results show the usefulness of understanding the transcriptional architecture of disease in order to dissect the mechanisms behind the appearance of a pathological phenotype.

## 2. Methods

**Figure 1:**
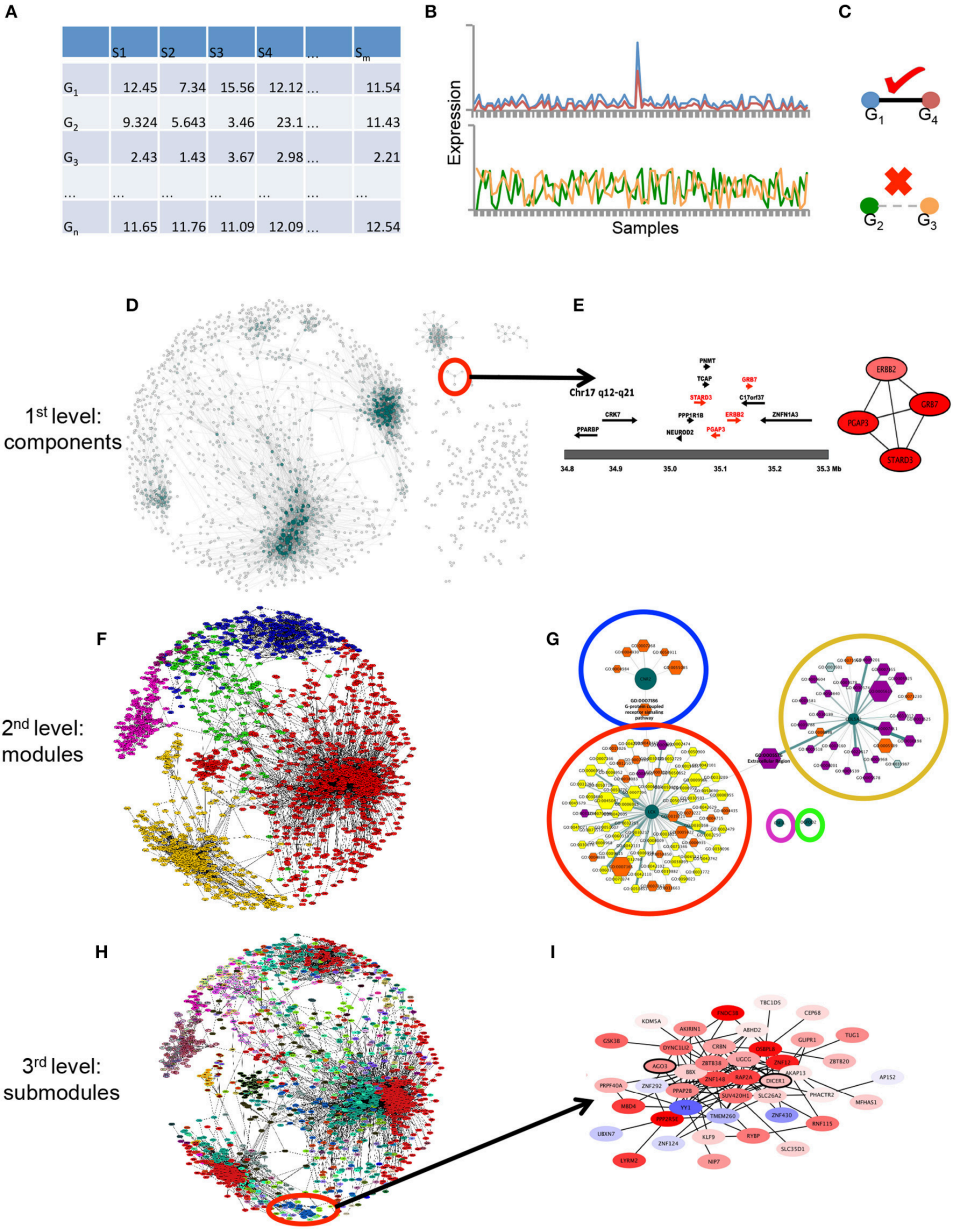
Graphical pipeline. **(A)** Data acquisition: The gene expression matrix (samples in columns, genes in rows) is generated with HER2+ samples from the datasets used in de Anda-Jáuregui et al. (2015). **(B)** Gene-gene relationships: By means of mutual information between couples of genes in **(A)**, gene expression across all samples are correlated. **(C)** High correlations are considered if they pass a certain threshold value (in this case the top 10,000 interactions). **(D)** First level of modularity: connected components. The largest level of modularity in this network is associated to certain structures, shown in **(E)**, the amplicon in Chr17 is depicted there. **(F)** Second level of modularity: Infomap-derived communities. In different colors, the modules generated by the infomap algorithm are depicted. **(G)** The aforementioned modules are enriched with Gene Ontology categories, which apparently represent specific categories (orange, violet and yellow nodes) per module (dark green centered nodes). **(H)** Third level of modularity: Hierarchical map equation. In different colorsthe modules obtained in **(F)** are systematically separated into submodules, and these are again enriched, as observed in **(I)**.

We used the *hierarchical map equation* (Rosvall and Bergstrom, 2011) to find nested submodules into the *HER2+ breast cancer network*, and then, using the approach of Alcalá-Corona et al. (2016), we identified whether those submodules were associated to a particular biological function. This section is divided as follows: network inference, differential expression analysis, modularity and submodularity detection and the hierarchical map equation, and functional analysis. A graphical representation of this methodology can be observed in Figure 1. It is worth mentioning that all the code used in this work is publicly available in our Github site https://github.com/CSB-IG/BioNetworkInference_ModularAnalysis.

### 2.1. Network inference

The network architecture of breast cancer molecular subtypes has been previously analyzed (de Anda-Jáuregui et al., 2016). There, network inference was carried out by using data on 493 microarray expression profiles for breast cancer samples processed on the Affymetrix HGU133A platform. Mutual information calculations were performed by means of the ARACNe algorithm (Margolin et al., 2006). PAM50-subtyped gene expression datasets were obtained as in de Anda-Jáuregui et al. (2015). From PAM50 algorithm we conserved the HER2+ subtypes only. In this work, we built upon such transcriptional network structure to carry out posterior analyses. As a result of this network inference, nodes represent genes in the transcriptional space and edges is the statistical dependence between two genes, which is a robust measure of the degree of co-expression existing in any couple of genes, and edges the statistical dependence between two genes, which is a robust measure of the degree of co-expression existing in any pair of genes.

### 2.2. Differential expression analysis

Independent gene-based linear models were adjusted using limma R package (R Core Team, 2013) to find differential expressed genes (DEGs) in the tumor samples compared to the control ones (61 samples in the same array, as described in de Anda-Jáuregui et al., 2016) using (1):

$$\begin{array}{r} y_{ij}=\mu_{+}\alpha_{i}+\varepsilon_{j} \end{array}$$

where *y*_*ij*_, is the *log*_2_(normalized gene expression); μ, is the global mean; α_*i*_ is the i*th* experimental condition (normal or tumor) and ε_*j*_*N*(0, σ) is the random error term of the j*th* sample. We also used a hypothesis tests based on empirical Bayes moderation of the standard errors toward a common value, in order to obtain *p*-values which were adjusted to control multiple comparisons using the False Discovery Rate (FDR) (Benjamini and Hochberg, 1995). We then defined a gene as differentially expressed if it had a *FDR* < 1 × 10^−5^ and a |*log*_2_(*foldchange*)| > 1. As it is observed, Figure 1B reflects how genes are grouped in a first modular structure, based on their differential expression, overexpressed genes have more overexpressed neighbors. Analogously, underexpressed genes are clustered with other underexpressed genes.

### 2.3. Modularity and submodularity detection and the hierarchical map equation

In order to find the hierarchical modular structure in the network, several methods to obtain modules from a given network have been developed (Girvan and Newman, 2002; Clauset et al., 2004; Adamcsek et al., 2006; Alves, 2007; Fortunato and Barthelemy, 2007; Arenas et al., 2008; Amini et al., 2013 for an in-depth revision, see Fortunato, 2010). Additionally, several of these algorithms have been used to find modules into gene regulatory networks (Dey and Meyer, 2015; Binder et al., 2016; Bonsang-Kitzis et al., 2016; Feng et al., 2016; Miecznikowski et al., 2016). For this work, we decide to use the generalization of the *map equation*, developed by Rosvall and Bergstrom (Rosvall and Bergstrom, 2011), as this is one of the best performing network partition algorithms as revealed by several stringent benchmark tests (Lancichinetti et al., 2009). In general, the hierarchical *map equation* method allows for multiple description levels of the movements of a random walker within the modules and allows an arbitrary number of such movements inside modules, submodules, subsubmodules, and so on, to the finest level. This additional description allows exploiting the fact that fine-level modules are themselves organized into larger modules. Modules and submodules are labeled according to the gene with the highest PageRank, a centrality measure that takes into account the cumulative weight of adjacent links (Brin and Page, 1998).

Graph partition by means of random walk-based methods (such as the method used here), generally speaking, optimize information flow within partitions and over the whole network. To do so, most of these algorithms perform constrained optimization over an entropic probability measure. In the present case, this is done by minimum description length optimization and bootstrapped permutation over an ensemble of encoded trajectories to generate an optimum partition. The outcome of the method is a maximum entropy partition. Hence, there is no need to evaluate different partitions, since there is no better partition compliant with the data than the one obtained. Infomap is one of the flagship of non-overlapping community detection methods. It has shown to be robust in the most stringent benchmarks (such as the LFR, Q-maximization and Spectral matrix-based tests), its computational complexity is linear in time, i.e., O(N). These facts have been already tested and are well established within the complex networks research community (Fortunato, 2010; Fortunato and Hric, 2016).

### 2.4. Functional analyses

Once the modules of the network have been detected, we explored whether the genes in these modules, arisen from the connections in the network (co-regulation model), are associated to a particular biological function. This is achieved by performing an *Over Representation Analysis (ORA)* based on the **hypergeometric test** over a category of genes whose function is known or annotated in a database. For this study, we use the *Gene Ontology Consortium* database **GO** (Ashburner et al., 2000). This *hypergeometric test* represents a *null model* to calculate how probable is it that a set of randomly chosen genes *k* belongs to a category (biological function) annotated in said database. Thus, a *p-value* is associated with this test, so the lower the *p-value* the lower the probability that the set *k* of genes belongs randomly to the category, and therefore represents a statistical trust about the particular gene set over the whole category. Additional analyses included the identification of differentially expressed genes (DEG) using the limma R package (Ritchie et al., 2015), and their functional characterization using Ingenuity Pathway Analysis (IPA) (Krämer et al., 2014).

### 2.5. Validation

In order to provide validation of our biological findings on an independent experimental dataset, we used 89 HER2+ samples from the METABRIC database. METABRIC (Molecular Taxonomy of Breast International Consortium) is a collection of clinically annotated primary fresh-frozen breast cancer specimens from tumor banks from the UK and Canada (Curtis et al., 2012), with transcriptomics data measured with the Illumina HT-12 v3 microarray platform.

Due to the differences in coverage between the Affymetrix 133A and the Illumina HT-12 v3 microarrays, we decided to discard for the downstream analysis genes that could not be simultaneously measured in both platforms. We used this filtered METABRIC expression matrix to infer the full set of Mutual Information values for all gene pairs using ARACNE. Then, this network was pruned by keeping the 10,000 gene pairs with the highest MI values, which formed our transcriptional network. The hierarchical modular structure of this transcriptional network was analyzed using the Infomap algorithm, and the resulting modules and submodules were inspected for functional associations following the same criteria previously used for the discovery network.

## 3. Results and discussion

Complex networks are mathematical models that represent the intricate interrelationship structure in complex systems. In the case of biological networks (in particular, gene regulatory networks), there is a search for modular partitions that may reflect a semi-mechanistic (ideally, a fully mechanistic) structure in which particular modules (and submodules) are responsible to carry out certain biological functions.

Conceptually, one may think of modules within a regulatory network that perform some functions in a way that it is not completely independent of the whole genome regulation program but is, to a certain extent, autonomous. This is thought to be so, since biological functions are often robust in their control and hence, resilient to potentially harmful systemic damage. Control theory has proven that one of the easiest way to combine robustness, with relative autonomy and global control is modularization.

The graph partition algorithm used here (InfoMap/MapEquation) is based on the consideration of an ensemble of random walkers performing stochastic trajectories over the network, a coding procedure is performed on the trajectories generating an ensemble of travel codes that are then subject to minimum description length optimization. The whole process is repeated in a large number of bootstrapped permutations that aside from the minimum description length optimization (and the fact that random walk trajectories are subject to the central limit theorem) ensures that one has the most probable modular partition of the network. Since this modular partition is subject to stringent optimization, we may refer to as the modular partition of the network, because even if it is not unique, is by far the most likely to happen.

### 3.1. The hierarchical structure of HER2+ network

**Figure 2:**
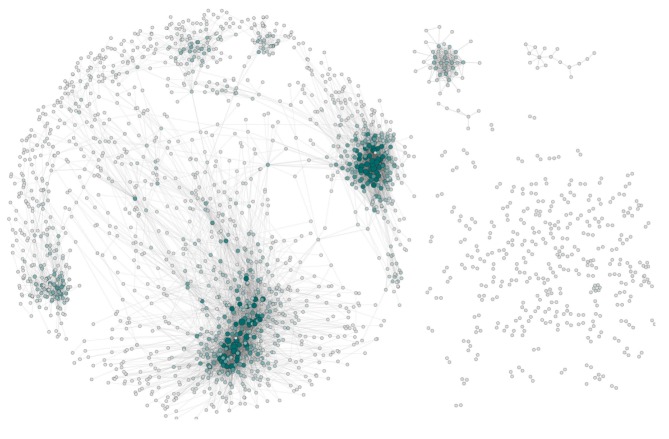
The network architecture of Her2+ breast cancer. The visualization shows the connected components (islands) of this network separately. In this representation nodes are colored and sized according to their node degree (i.e., the number of neighbors connected to a gene). The layout and arrangement of the network showcases the giant component **(Left)** and several small islands **(Right)**.

Transcriptional networks associated with specific molecular subtypes of breast cancer have characteristic structures (de Anda-Jáuregui et al., 2016). In the case of the HER2+ molecular subtype, we can observe a network with 2,100 nodes and 9,856 edges; this network is integrated by a giant component, along with several (161) smaller connected components or islands (Figure 2). The basic topological parameters of this network are shown in Table 1.

**Table 1:** Network metrics.

**Parameter**	**Value**
Nodes	2,100
Edges	9,856
Avg. node degree	4,583
Char. path length	5,917
Connected components	162

Breast cancer transcriptional networks also exhibit modular structures, as it has been previously reported (Alcalá-Corona et al., 2017). The HER2+ molecular subtype is no exception. The first level of modularity observed in this network is the aforementioned distribution of nodes into connected components. As it is shown in Figure 1, the bulk of connections in the network are part of the giant component. Moreover 1,649 out of the total number of nodes are part of it. It is in the giant component where we can analyze the modular hierarchical structure of HER2+ breast cancer network.

**Figure 3:**
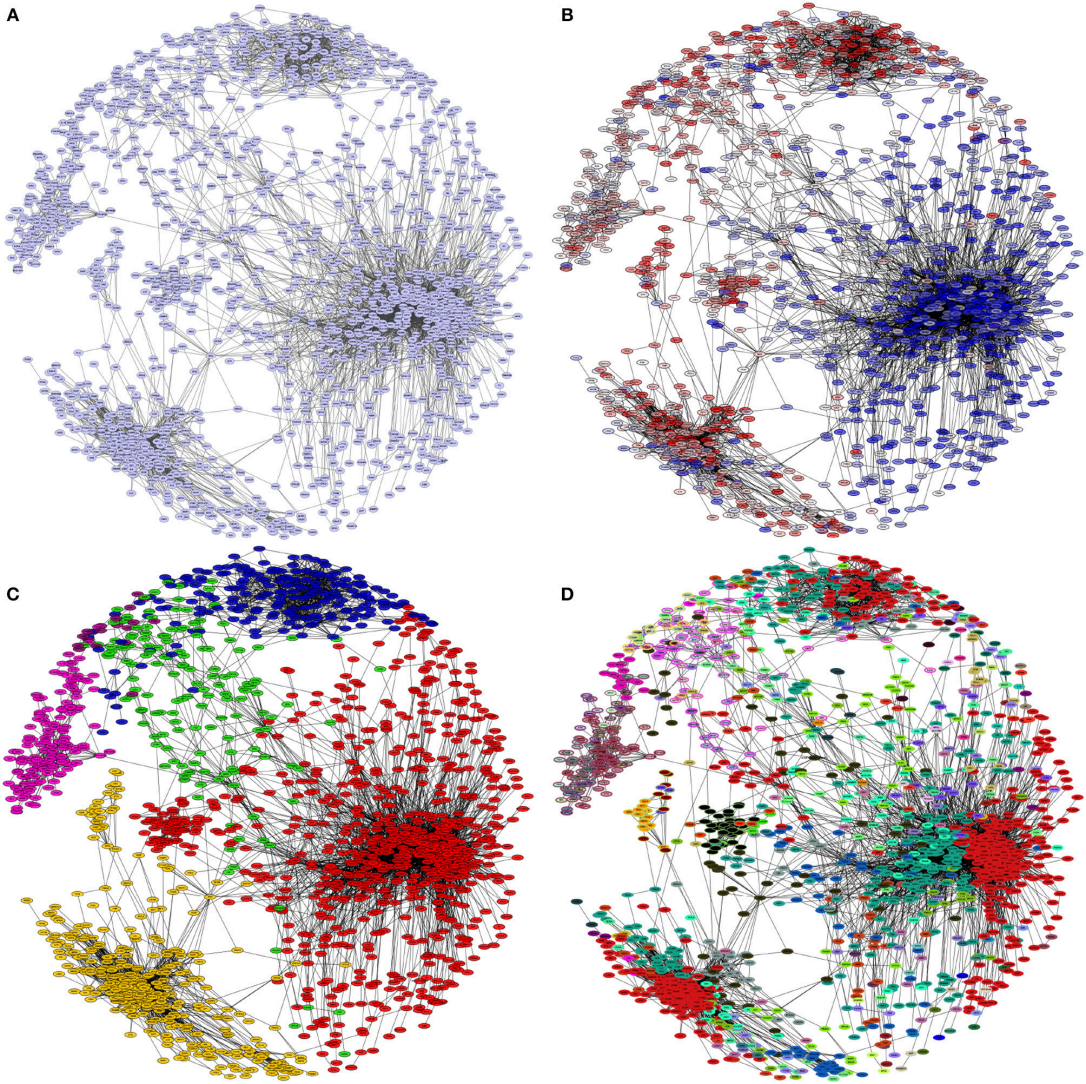
Hierarchical levels of modularity in the HER2+ network. In each panel, the giant component of the HER2+ breast cancer network is depicted. The colors of the nodes in each panel represent different groupings: **(A)** all nodes are colored the same, as they belong to the same connected component. **(B)** Nodes are colored by expression levels (blue: underexpressed, red: overexpressed) regardless of connectivity patterns. Notice that genes are grouped together depending on the differential expression pattern. **(C)** Nodes are colored according to modules detected by Infomap; in **(D)** nodes are colored by submodules inside the modules, these submodules were detected using the hierarchical *map equation*.

In Figure 3 we show different visualizations of the giant component, highlighting different properties: In panel A, we show this largest component using a spring-embedded layout. In panel B, we color the nodes according to differential expression (ranging from blue to red in terms of under and over expression); nodes with similar expression values are arranged together and have a higher density of connections among them, which is in accordance to the fact that connections in this network arise from common expression patterns. This in turn leads to panels C and D, that show modules and submodules, respectively, based on the connectivity patterns exhibited in the network, as identified using the Infomap algorithm based on the hierarchical *map equation*.

Previously, we have shown that modular structures in transcriptional networks are usually associated with biological features (Alcalá-Corona et al., 2016, 2017). Here we show that new insights on the biological features of HER2+ breast cancer may be revealed by exploring the functionality related to different layers of modularity.

### 3.2. The HER2 amplicon genes are modularly isolated in the transcriptional network

As we have previously mentioned, the nodes that integrate the HER2+ transcriptional network are distributed in 162 different connected components. The vast majority of nodes belong to a single giant component, whereas the rest of connected components are much smaller: the next component in size has only 39 nodes, and 120 components are composed by two nodes each. At this level of modularity, it is hard to systematically analyze functionality of connected components, as it is unlikely to identify biological functions and processes that can be associated with two or three molecules alone.

**Figure 4:**
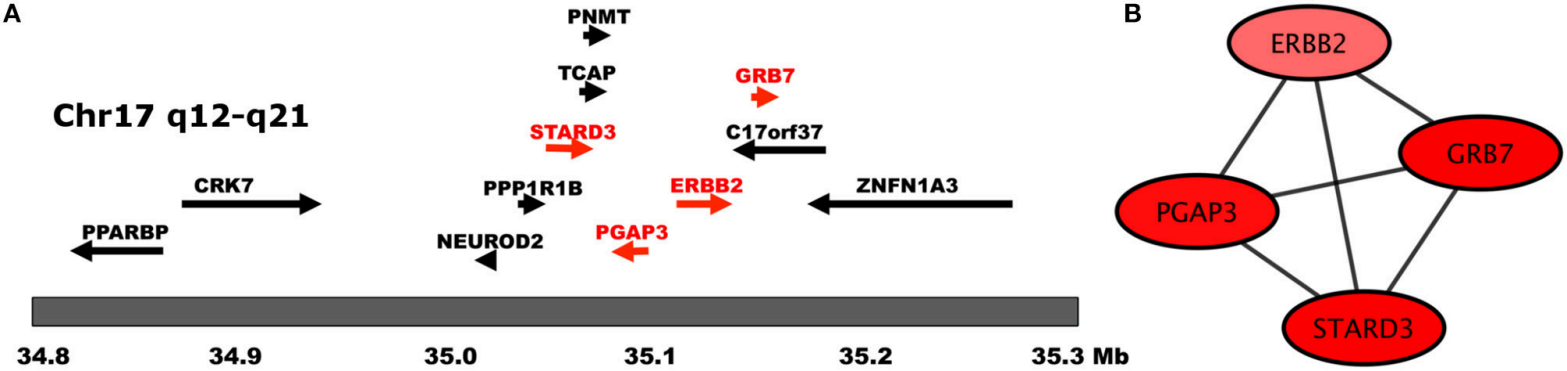
Genes of the HER2 Amplicon. In this figure we show **(A)** the chromosomal location of HER2 Amplicon genes (adapted from Kauraniemi and Kallioniemi, 2006), and **(B)** The transcription interactions of those genes in the regulatory network. Notice that in **(B)**, only genes belonging to the amplicon appear in said component.

However, an important exception is the case of a component integrated by four genes that belong to the HER2 amplicon: ERBB2, GRB7, PGAP3, and STARD3. These four genes are closely located at the core of the HER2 amplicon, which is the single, most important hallmark of this particular subtype of cancer (Kauraniemi and Kallioniemi, 2006). Their fully connected pattern (clique), is indicative of a close coexpression exhibited by these four genes, which may be related to their genomic proximity in the amplified region (Figure 4). In previous work (Espinal-Enríquez et al., 2017) we have shown that transcriptional networks of breast cancer recover connections between genes belonging to the same chromosome, which are related to a loss of trans regulation associated with breast cancer. In the case of the network analyzed in this work, it appears that the more statistically significant connections that are recovered for the ERBB2 gene are to its closest genomic neighbors.

### 3.3. Functional role of modules in HER2 transcriptional network

**Figure 5:**
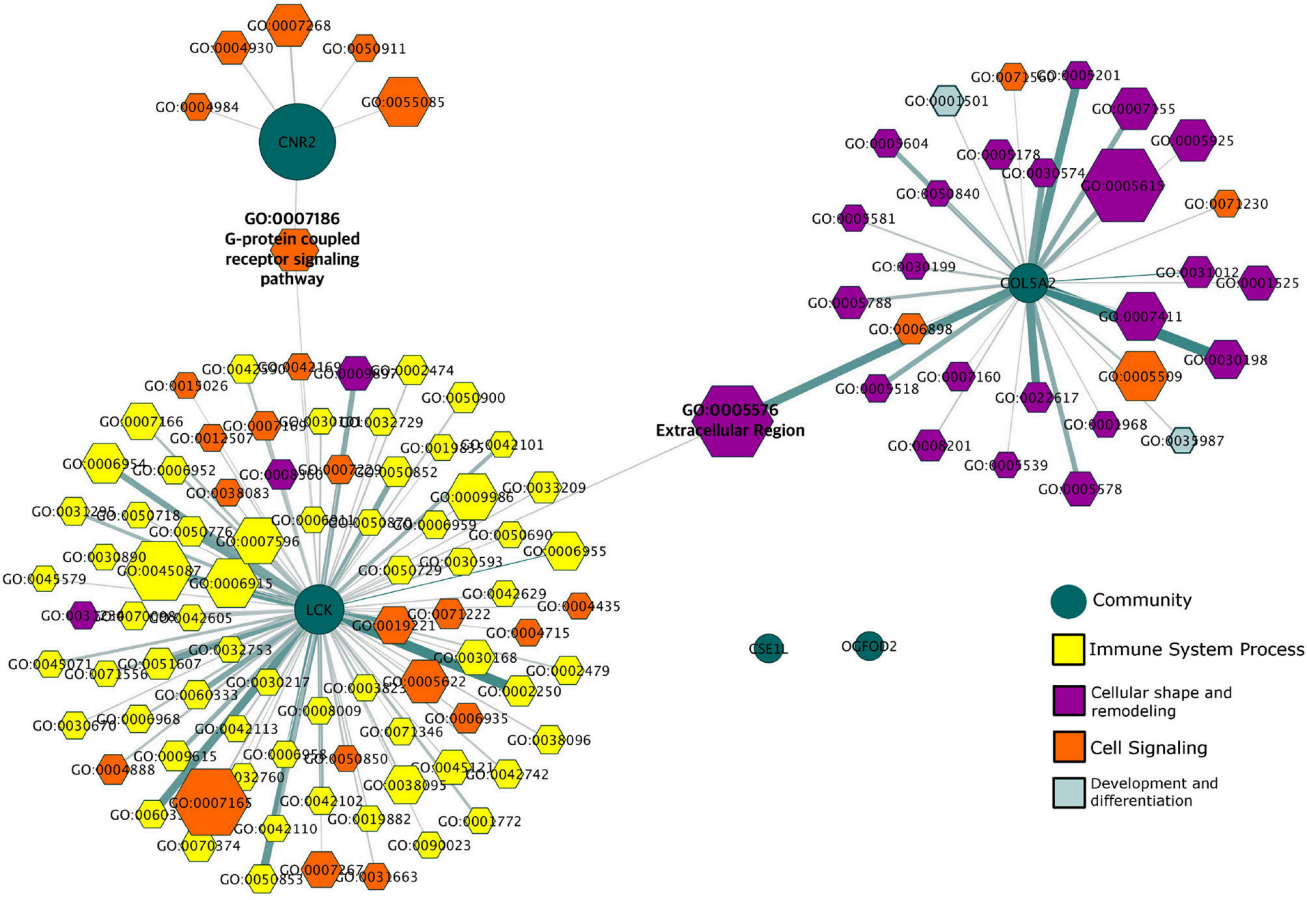
Gene Ontology Categories associated with HER2+ modules. This network depicts the different biological features (hexagons) to which the modules (green circles) in the giant component of the HER2+ network are associated. Processes are colored according to the general biological function in which they participate.

To analyze the modular structure of the giant component and their functionality is important to have a more detailed vision of these genes in the disease. This giant component has a modular structure composed of six modules. In Figure 5 we see that the functionality associated with these different modules can be clearly divided along functional themes, indicating a functional compartmentalization reflected in the transcriptional program.

We can observe that the three largest modules (labeled CNR2, LCK, and COL5A2 based on the genes with the highest PageRank in each module) are related to processes of signaling and transport, immunity, and extracellular matrix organization respectively. We can also observe the existence of two processes that connect these communities at a functional level: GO:007186 (G-protein coupled receptor signaling pathway) which is associated with both the CNR2 and the LCK modules; and GO:0005576 (Extracellular region) which is associated with both the LCK and COL5A2 modules. In this regard, it is important to recall that the hierarchic *map equation* recovers non-overlapping modules: therefore, the fact that these processes can be associated with two distinct modules indicates that the regulation of those processes involves two different sets of genes.

#### 3.3.1. The CNR2 submodules are associated with the regulation of signaling and micro-RNA assembly

The largest module in the giant component of the HER2+ network has 763 genes with 4,574 links between them. The gene with the highest PageRank in this community is the CNR2 gene, which codifies for the cannabinoid receptor type 2 (CB2). This CNR2 module is composed by 49 submodules. Additionally, two of these modules: GPATCH4 and ZBTB38, have sub-submodular structures themselves (with 4 and 5 sub-submodules, respectively). As mentioned before, the CNR2 module is associated with signaling and transport processes. Interestingly, few of their submodules show enrichment (each submodule contains genes that participate in a specific function): these are the CNR2, PGLYRP4, and ZBTB38 submodules.

The aforementioned modules may be associated with specific cellular components, with the first two modules being involved in the cell membrane, while the last one correlates to the cytosolic region. Furthermore, the ZBTB38 submodule (defined by the gene that encodes zinc finger and BTB domain containing 38) is also associated with protein binding processes; taken into account together, this may be indicative that the ZBTB38 submodule is more related to intracellular signaling than to membrane receptor-dependent signaling.

**Figure 6:**
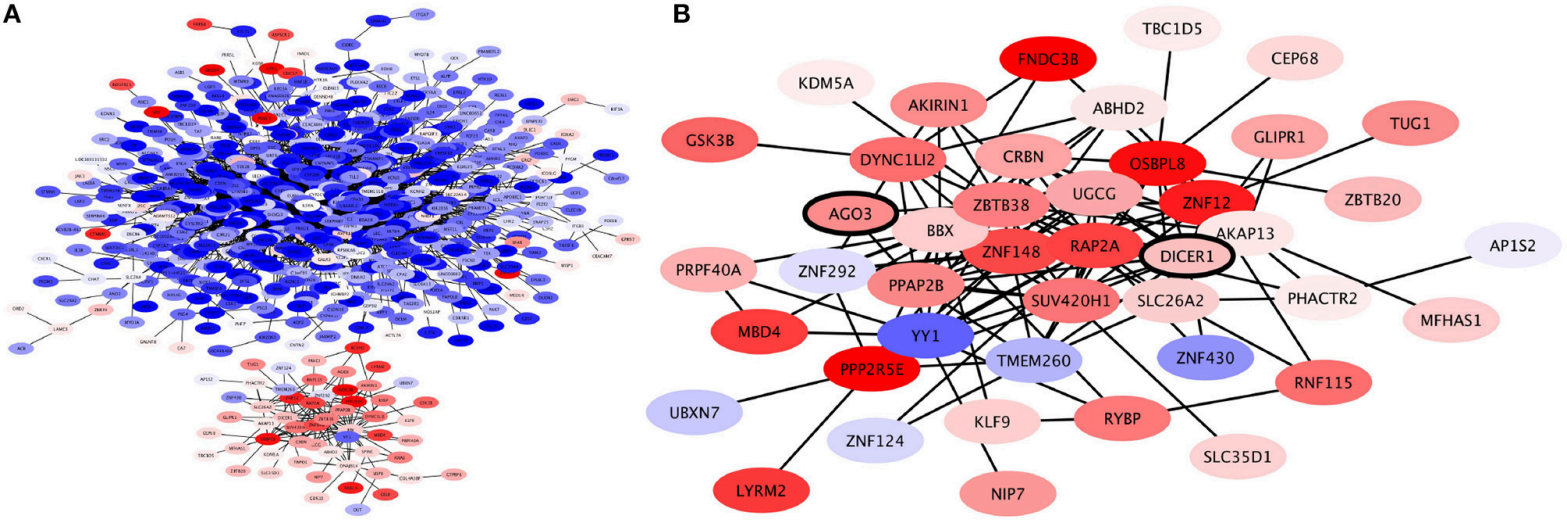
Expression profile of CNR2 Community. **(A)** This community is roughly divided in two based on expression levels: the genes in the communities depicted on the upper side of the network are underexpressed (blue); these are genes involved in plasma-membrane associated processes. Meanwhile, the genes in the community depicted at the bottom are overexpressed; these are genes involved in intracellular signaling. **(B)** Expression profile of ZBTB38 Submodule of CNR2 module. Genes in this module are mostly overexpressed. Two of the genes in this submodule are DICER and AGO3, crucial elements of micro-RNA regulation (bold).

When the expression levels of these genes are taken into account (as seen in Figure 6A) we may observe a clear separation in this submodule, where only the ZBTB38 submodule is overexpressed, while the rest of the submodules in the CNR2 module are underexpressed. Inside the ZBTB38 we can also find two crucial genes for micro-RNA assembly and regulation: DICER1 and AGO (Figure 6B). These two are the main components in the RISC complex (Daugaard and Hansen, 2017). It has been shown that micro-RNA regulation is crucial for the induction of both epithelial-to-mesenchymal transition and mesenchymal-to-epithelial processes in breast cancer Drago-Garca et al. (2017). Hence a submodule related to micro-RNA-gene regulation may be relevant to identify novel therapeutic options.

#### 3.3.2. The submodules of COL5A2 are involved in extracellular matrix organization

A module containing 169 nodes and 789 edges was identified in the giant component, containing the COL5A2 gene as the node with the highest PageRank. This COL5A2 module was associated with Extracellular Matrix (ECM) organization. This module in turn is composed by 18 submodules, with COL5A2 being the defining gene of the largest one. This COL5A2 submodule again is associated with ECM organization, collagen organization and cell adhesion. The COL5A2 gene codifies for the Collagen 5A2 protein, a key participant in shaping the ECM. Other genes in this subcommunity that are widely known to play important roles in ECM organization include genes such as LUM, fibronectin, VCAN and members of the collagen family beyond COL5A2, among others.

**Figure 7:**
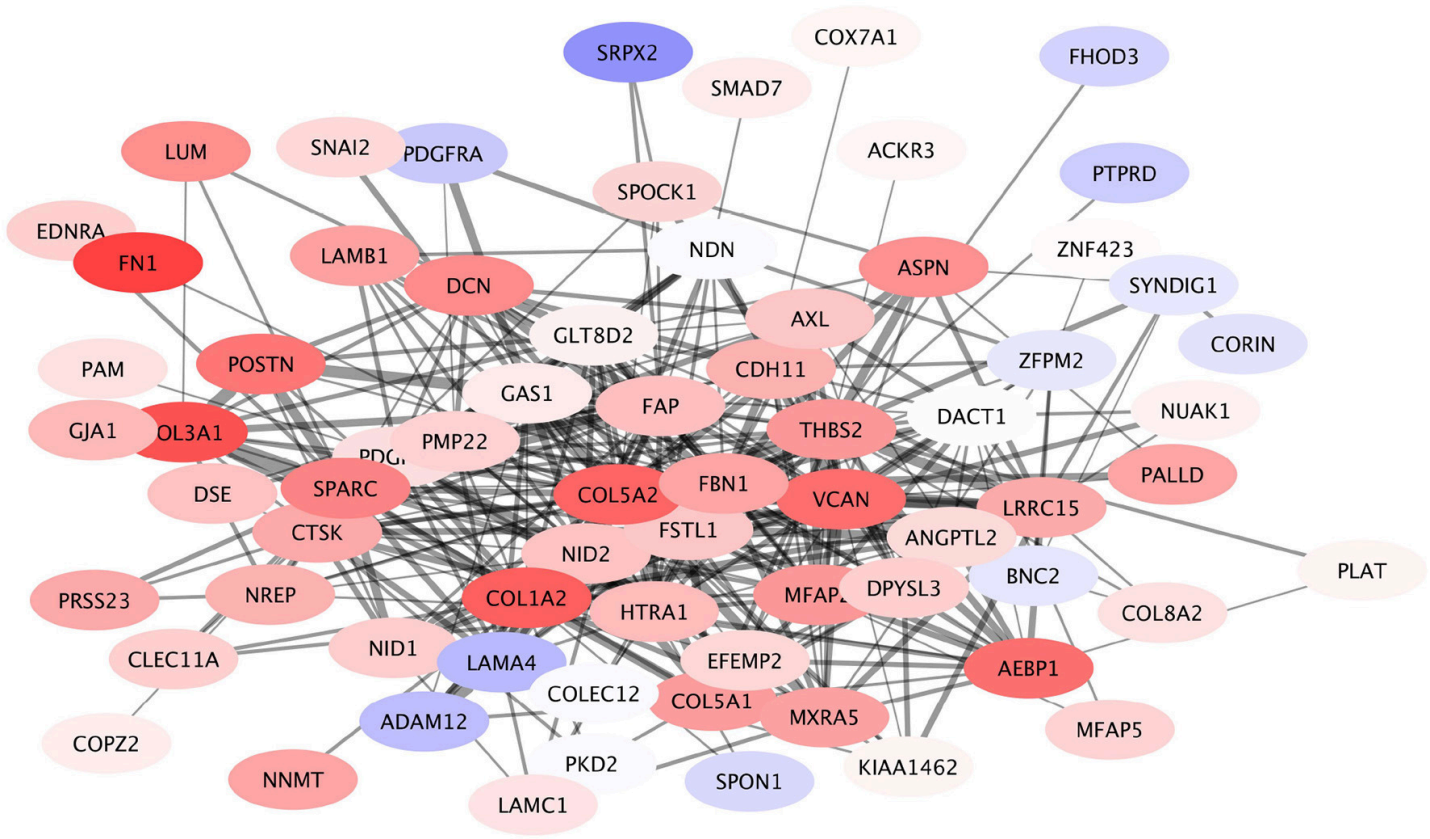
Expression profile of COL5A2 module. This submodule is composed mostly by overexpressed genes. Interestingly the underexpressed genes (depicted in light blue) have a small number of connections, compared to the number of links that the majority of overexpressed genes have.

In Figure 7 the expression patterns of these genes can be seen. It shows that overall the members of this submodule are overexpressed. This is a clear indicative that in HER2+ breast cancer subtype, remodeling of extracellular matrix is a key participant in shaping the phenotype, it is fundamental for invasiveness, migration, Epithelial-to-mesenchymal transition (EMT) and other processes ubiquitous in cancer.

#### 3.3.3. The role of the submodules of LCK in immune response

**Figure 8:**
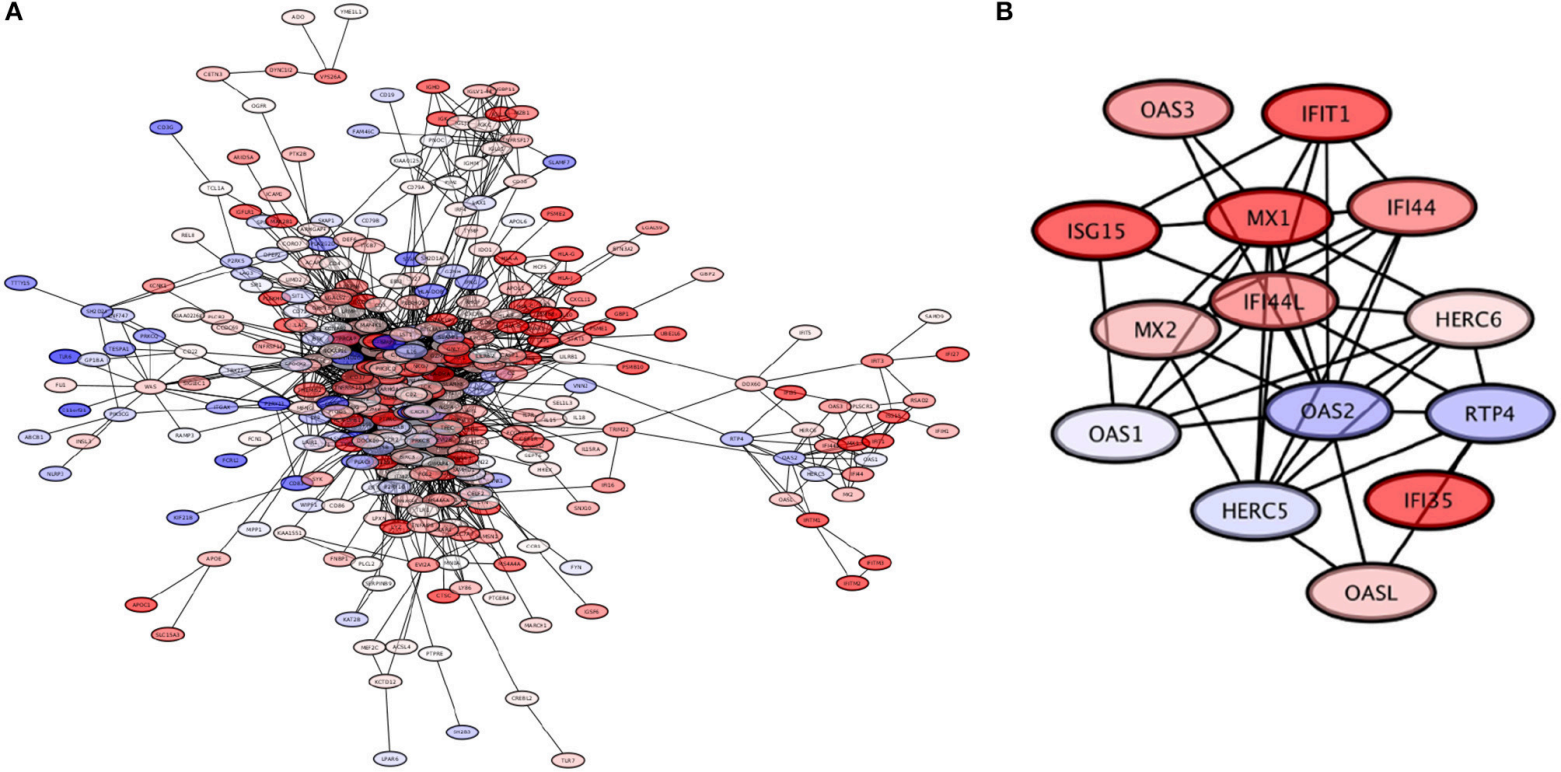
The LCK module. **(A)** The LCK module, with genes colored based on their expression levels. **(B)** The OAS2L submodule of the LCK module, which exhibits mostly overexpressed genes; this module is involved in processes related to response to viral infection.

The third largest module of the giant component is composed by 371 genes with 3,011 connections among them, with the highest *Page-Ranked* one being the LCK gene (which encodes the lymphocyte-specific protein tyrosine kinase). This module is subdivided into 27 submodules, with one of them (the OAS2L module) in turn having three sub-submodules. In Figure 8A, a visualization of this module is provided.

The LCK module is associated to the functioning of the immune system. In turn, five submodules of LCK: LCK, OAS2, PSMB9, SLAMF8, and TNFRSF17 show enrichment, again related to functions of the immune system. The OAS2L submodule in particular called our attention. At the level of submodular structure, this module can be associated with processes related to the response to viral infections: “defense response to virus,” “type I interferon signaling pathway,” “response to virus,” and “negative regulation of viral genome replication.” When the three sub-submodules of OAS2L (DDX60, IFITM1, OAS2) are analyzed independently, they in turn are associated with seven processes that are also involved in viral infection response: this includes the aforementioned “defense response to virus,” “type I interferon signaling pathway,” “response to virus,” and “negative regulation of viral genome replication,” as well as new emerging processes “response to interferon-gamma,” “negative regulation of viral entry into host cell,” and “interferon-gamma-mediated signaling pathway.”

**Figure 9:**
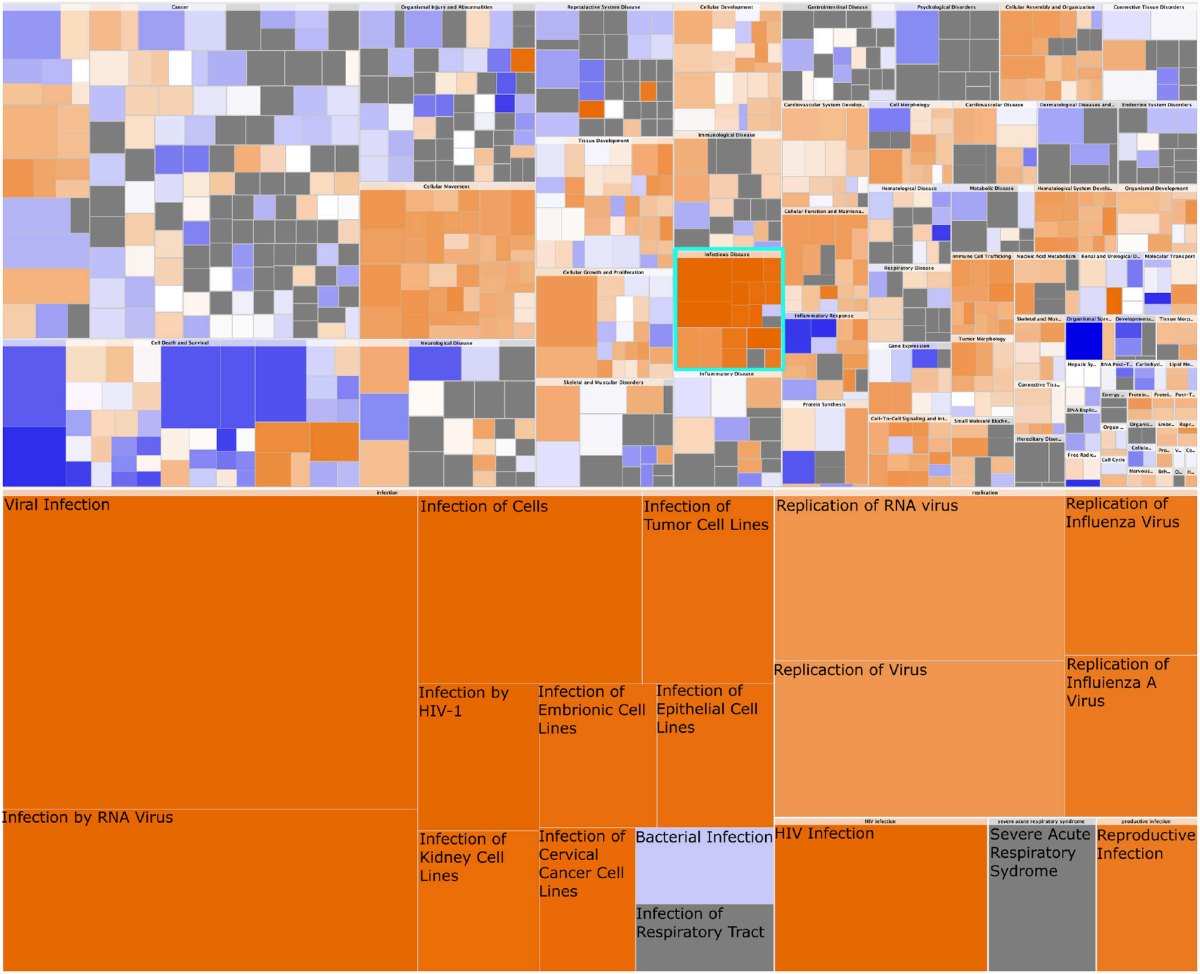
Heatmap of Diseases and Functions associated with HER2+ breast cancer. In this figure we observe the whole set of Diseases and Functions associated with the gene expression signature of HER2+ breast cancer. The heatmap in the upper part represents High-level functional categories: Cancer, Cellular movement, etc. Square color is the z-score of the function, it reflects the direction of change of the function, based on the differential expression of the genes in said function. Orange color represent a positive z-score, which indicates a trend toward an increase. Blue squares represent a decrease. Square size is proportional to the number of genes in said function. In turquoise is delimited the High-level category corresponding to *Infectious Disease*. This category is zoomed-in at the bottom of the figure. Notice that every process but *bacterial infection* are consistently increased, and those correspond to viral infection-related processes.

These results are complemented by the findings of analyzing the gene expression data using Ingenuity Pathway Analysis. The results of such analysis are shown in Figure 9. In particular, we highlight the appearance of the “infectious diseases” category; importantly, notice that the response detected is associated only to viral (as opposed to, for instance, bacterial) infection. The expression patterns of the OAS2L submodule (as seen in Figure 8B), leads us to argue that an activation of immunity with mechanisms used for the response to viral infections is found in HER2+ breast cancer subtype.

### 3.4. Validation

In the validation network we were able to recover several of the previously discussed findings regarding modular structure and biological functional associations (see Supplementary Material).

By applying the same methodology as in our discovery approach, we were able to recover an independent component that is composed by genes in the HER2 amplicon. In the discovery network, this component contains STARD3, GRB7, PGAP3 and ERBB2, whereas in the validation network it was composed by, GRB7, PGAP3, MIEN1, TCAP and ERBB2. MIEN1 and TCAP are also part of the amplicon. In both cases the important functional finding is the tight co-expression of the genes in the amplicon, and how these are completely isolated from other elements of the transcriptional network, including the largest connected component.

A largest connected component also appears in the validation network. As previously mentioned, this component is completely disconnected from HER2 amplicon regulation, suggesting independent mechanisms of regulation. We were also able to recover modular and sub-modular structure in this largest connected component with similar functional associations to those found in the discovery network. Some of these functional enrichments are described in the following lines:

We recovered a module containing several collagen-encoding genes, analogous to the COL5A2 module in the discovery network. Furthermore, this module was significantly associated to Extracellular Matrix processes, as well as other structural functions, concordant with the significant functional associations found in the discovery network.

We were also able to find a module functionally associated to immune system processes, as observed in the LCK module in the discovery network; in both cases, these were the largest modules. Furthermore, this validation immune module contain modular substructures, including a small group (25 genes), including OAS1, 2, and 3, as well as interferon alpha-inducing protein family members. This submodule was statistically associated to viral response processes, just like the one found in the discovery network.

Even though the node composition of these modules was not identical (due to the aforementioned differences in microarray technologies), they were functionally coincident. At the level of inquiry used here, some findings were not replicated in the validation network, in particular a module associated to cell signaling processes in the largest connected component.

## 4. Conclusions

The modular structure of transcriptional networks and its relationship to biological functionality are topics of current biomedical interest. In this work, we have implemented a hierarchical module detection method to identify the highest resolution of modular structure of the transcriptional network of HER2+ breast cancer, and the functions associated with each network module.

Using this approach, we have identified biological features associated with different levels of modular structure in this network. At the highest level of modularity we observe a distribution of genes into different connected components, with more than half the genes detected belonging to a giant connected component. Furthermore, we may observe connectivity among genes of the HER2-amplicon, the most important genomic element associated with the development of HER2+ breast cancer, in an independent, specific connected component.

At a higher modular resolution, we identified communities in the largest connected component, some of which are statistically associated with sets of Gene Ontology categories related to specific biological functions: immune system processes, cellular shape and remodeling, and cell signaling. Furthermore, some of these Gene Ontologies are associated with more than one non-overlapping module, indicating the need for joint regulation from specialized sets of genes.

Finally, submodular structures in the HER2+ network reveal finer details of the processes involved in the pathological state. For instance, specific modules responsible of the regulation of intracellular signaling, micro-RNA assembly, and viral infection were identified inside, more general modules. As such, we show that a higher modular resolutions allows for the emergence of more specific biological function regulation.

Our work showcases the importance and usefulness of analyzing the phenomenon of transcriptional regulation using a complex network approach. We present cases in which the study of the modular structures beneath the transcriptional network architecture unveil mechanisms associated with the pathological state, which may lead to insights relevant to the biomedical community.

## Author contributions

SAA-C contributed with methodological tools, performed calculations and analyses, collaborated in the theoretical design and discussion of results, and collaborated in writing and revising the paper. JE-E contributed with methodological tools, collaborated in the theoretical design and discussion of results, and collaborated in writing and revising the paper. GdA-J contributed with methodological tools, collaborated in the theoretical design and discussion of results, and collaborated in writing and revising the paper. EH-L contributed to the general design and supervision of the project, contributed with methodological tools, collaborated in the theoretical design and discussion of results, and collaborated in writing and revising the paper.

### Conflict of interest statement

The authors declare that the research was conducted in the absence of any commercial or financial relationships that could be construed as a potential conflict of interest.
