# Supplementary material for: The Hierarchical Modular Structure of HER2+ Breast Cancer Network
Source: Front Physiol. 2018 Oct 11;9:1423. doi: 10.3389/fphys.2018.01423 (PMC6193406; doi:10.3389/fphys.2018.01423)
Supplement: Supplementary file 1 [file Data_Sheet_1.zip › EnrichmentResults.zip!/ECM_EnrichmentValidation.gz!/./Report_wg_result1531781385.html]

WebGestalt (WEB-based GEne SeT AnaLysis Toolkit)


- Summary
- User ID Mapping Table
- GOSlim Summary
- Enrichment Results

### Summary   (Result Download)

**Enrich method:** ORA  
**Organism:**hsapiens  
**Enrichment Categories:** geneontology\_Biological\_Process  
**Interesting gene list:** textAreaUpload\_1531781385.txt. **ID type:** genesymbol  
The interesting gene list contains **123** user IDs in which **122** user IDs are unambiguously mapped to the unique Entrez Gene IDs and **1** user IDs are mapped to multiple Entrez Gene IDs or could not be mapped to any Entrez Gene ID. The GO Slim summary are based upon the **122** unique Entrez Gene IDs.  
Among the **122** unique Entrez Gene IDs, **114** IDs are annotated to the selected functional categories and also in the reference gene list, which are used for the enrichment analysis.  
**Reference gene list:**  all mapped Entrez Gene IDs from the selected platform genome  
The reference gene list contains **60119** IDs and  **16258** IDs are annotated to the selected functional categories that are used as the reference for the enrichment analysis.  
**Parameters for the enrichment analysis:**

- **Minimum number of Entrez Gene IDs in the category:**5
- **Maximum number of Entrez Gene IDs in the category:**2000
- **FDR Method:**BH
- **Significance Level:** Top10

Based on the above parameters, **10** categories are identified as enriched categories and all are shown in this report.

#### Mapped User IDs

| userid | Gene Symbol | Gene Name | Entrez Gene |
| --- | --- | --- | --- |
| CDH11 | CDH11 | cadherin 11 | 1009 |
| LRRC17 | LRRC17 | leucine rich repeat containing 17 | 10234 |
| MYL9 | MYL9 | myosin light chain 9 | 10398 |
| SPON1 | SPON1 | spondin 1 | 10418 |
| POSTN | POSTN | periostin | 10631 |
| CORIN | CORIN | corin, serine peptidase | 10699 |
| FERMT2 | FERMT2 | fermitin family member 2 | 10979 |
| EMILIN1 | EMILIN1 | elastin microfibril interfacer 1 | 11117 |
| FSTL1 | FSTL1 | follistatin like 1 | 11167 |
| PRKCDBP | PRKCDBP | protein kinase C delta binding protein | 112464 |
| TMEM200A | TMEM200A | transmembrane protein 200A | 114801 |
| CTHRC1 | CTHRC1 | collagen triple helix repeat containing 1 | 115908 |
| COL1A1 | COL1A1 | collagen type I alpha 1 chain | 1277 |
| COL1A2 | COL1A2 | collagen type I alpha 2 chain | 1278 |
| COL3A1 | COL3A1 | collagen type III alpha 1 chain | 1281 |
| COL5A1 | COL5A1 | collagen type V alpha 1 chain | 1289 |
| COL5A2 | COL5A2 | collagen type V alpha 2 chain | 1290 |
| COL6A1 | COL6A1 | collagen type VI alpha 1 chain | 1291 |
| COL6A3 | COL6A3 | collagen type VI alpha 3 chain | 1293 |
| COL8A2 | COL8A2 | collagen type VIII alpha 2 chain | 1296 |
| COL10A1 | COL10A1 | collagen type X alpha 1 chain | 1300 |
| COL11A1 | COL11A1 | collagen type XI alpha 1 chain | 1301 |
| COL16A1 | COL16A1 | collagen type XVI alpha 1 chain | 1307 |
| LRRC15 | LRRC15 | leucine rich repeat containing 15 | 131578 |
| VCAN | VCAN | versican | 1462 |
| CSPG4 | CSPG4 | chondroitin sulfate proteoglycan 4 | 1464 |
| CTSK | CTSK | cathepsin K | 1513 |
| CD55 | CD55 | CD55 molecule (Cromer blood group) | 1604 |
| DCN | DCN | decorin | 1634 |
| AEBP1 | AEBP1 | AE binding protein 1 | 165 |
| DPYSL3 | DPYSL3 | dihydropyrimidinase like 3 | 1809 |
| ECM2 | ECM2 | extracellular matrix protein 2 | 1842 |
| EDNRA | EDNRA | endothelin receptor type A | 1909 |
| FBLN1 | FBLN1 | fibulin 1 | 2192 |
| FBN1 | FBN1 | fibrillin 1 | 2200 |
| ITGA11 | ITGA11 | integrin subunit alpha 11 | 22801 |
| PALLD | PALLD | palladin, cytoskeletal associated protein | 23022 |
| PHLDB1 | PHLDB1 | pleckstrin homology like domain family B member 1 | 23187 |
| ANGPTL2 | ANGPTL2 | angiopoietin like 2 | 23452 |
| KIAA1549L | KIAA1549L | KIAA1549 like | 25758 |
| MXRA5 | MXRA5 | matrix remodeling associated 5 | 25878 |
| OLFML2B | OLFML2B | olfactomedin like 2B | 25903 |
| ZNF521 | ZNF521 | zinc finger protein 521 | 25925 |
| SETBP1 | SETBP1 | SET binding protein 1 | 26040 |
| ZBTB20 | ZBTB20 | zinc finger and BTB domain containing 20 | 26137 |
| LRRC32 | LRRC32 | leucine rich repeat containing 32 | 2615 |
| GAS1 | GAS1 | growth arrest specific 1 | 2619 |
| HEYL | HEYL | hes related family bHLH transcription factor with YRPW motif-like | 26508 |
| GJA1 | GJA1 | gap junction protein alpha 1 | 2697 |
| SRPX2 | SRPX2 | sushi repeat containing protein, X-linked 2 | 27286 |
| OLFML1 | OLFML1 | olfactomedin like 1 | 283298 |
| PTRF | PTRF | polymerase I and transcript release factor | 284119 |
| SSC5D | SSC5D | scavenger receptor cysteine rich family member with 5 domains | 284297 |
| DSE | DSE | dermatan sulfate epimerase | 29940 |
| EFEMP2 | EFEMP2 | EGF containing fibulin like extracellular matrix protein 2 | 30008 |
| CFH | CFH | complement factor H | 3075 |
| EHD2 | EHD2 | EH domain containing 2 | 30846 |
| IGFBP4 | IGFBP4 | insulin like growth factor binding protein 4 | 3487 |
| LAMA4 | LAMA4 | laminin subunit alpha 4 | 3910 |
| LUM | LUM | lumican | 4060 |
| MAP1A | MAP1A | microtubule associated protein 1A | 4130 |
| MMP2 | MMP2 | matrix metallopeptidase 2 | 4313 |
| MMP11 | MMP11 | matrix metallopeptidase 11 | 4320 |
| MMP16 | MMP16 | matrix metallopeptidase 16 | 4325 |
| MXRA7 | MXRA7 | matrix remodeling associated 7 | 439921 |
| NDN | NDN | necdin, MAGE family member | 4692 |
| NID1 | NID1 | nidogen 1 | 4811 |
| NNMT | NNMT | nicotinamide N-methyltransferase | 4837 |
| OMD | OMD | osteomodulin | 4958 |
| PAM | PAM | peptidylglycine alpha-amidating monooxygenase | 5066 |
| COPZ2 | COPZ2 | coatomer protein complex subunit zeta 2 | 51226 |
| DACT1 | DACT1 | dishevelled binding antagonist of beta catenin 1 | 51339 |
| PDGFRL | PDGFRL | platelet derived growth factor receptor like | 5157 |
| PDGFRB | PDGFRB | platelet derived growth factor receptor beta | 5159 |
| SERPINF1 | SERPINF1 | serpin family F member 1 | 5176 |
| PKD2 | PKD2 | polycystin 2, transient receptor potential cation channel | 5311 |
| PLAT | PLAT | plasminogen activator, tissue type | 5327 |
| BNC2 | BNC2 | basonuclin 2 | 54796 |
| TENM3 | TENM3 | teneurin transmembrane protein 3 | 55714 |
| AXL | AXL | AXL receptor tyrosine kinase | 558 |
| PROS1 | PROS1 | protein S (alpha) | 5627 |
| HTRA1 | HTRA1 | HtrA serine peptidase 1 | 5654 |
| PLSCR4 | PLSCR4 | phospholipid scramblase 4 | 57088 |
| PTH1R | PTH1R | parathyroid hormone 1 receptor | 5745 |
| TSHZ3 | TSHZ3 | teashirt zinc finger homeobox 3 | 57616 |
| ACTA2 | ACTA2 | actin, alpha 2, smooth muscle, aorta | 59 |
| BGN | BGN | biglycan | 633 |
| SFRP2 | SFRP2 | secreted frizzled related protein 2 | 6423 |
| SGCD | SGCD | sarcoglycan delta | 6444 |
| SNAI2 | SNAI2 | snail family transcriptional repressor 2 | 6591 |
| SPARC | SPARC | secreted protein acidic and cysteine rich | 6678 |
| SPOCK1 | SPOCK1 | SPARC/osteonectin, cwcv and kazal like domains proteoglycan 1 | 6695 |
| TAGLN | TAGLN | transgelin | 6876 |
| TBX15 | TBX15 | T-box 15 | 6913 |
| TCF4 | TCF4 | transcription factor 4 | 6925 |
| TGFB1I1 | TGFB1I1 | transforming growth factor beta 1 induced transcript 1 | 7041 |
| THBS2 | THBS2 | thrombospondin 2 | 7058 |
| THY1 | THY1 | Thy-1 cell surface antigen | 7070 |
| TIMP2 | TIMP2 | TIMP metallopeptidase inhibitor 2 | 7077 |
| C1R | C1R | complement C1r | 715 |
| TPM2 | TPM2 | tropomyosin 2 (beta) | 7169 |
| TRO | TRO | trophinin | 7216 |
| ARHGAP28 | ARHGAP28 | Rho GTPase activating protein 28 | 79822 |
| SYNDIG1 | SYNDIG1 | synapse differentiation inducing 1 | 79953 |
| BICC1 | BICC1 | BicC family RNA binding protein 1 | 80114 |
| FHOD3 | FHOD3 | formin homology 2 domain containing 3 | 80206 |
| MFAP5 | MFAP5 | microfibrillar associated protein 5 | 8076 |
| GLT8D2 | GLT8D2 | glycosyltransferase 8 domain containing 2 | 83468 |
| TMEM47 | TMEM47 | transmembrane protein 47 | 83604 |
| JAM3 | JAM3 | junctional adhesion molecule 3 | 83700 |
| CRISPLD2 | CRISPLD2 | cysteine rich secretory protein LCCL domain containing 2 | 83716 |
| SPARCL1 | SPARCL1 | SPARC like 1 | 8404 |
| SRPX | SRPX | sushi repeat containing protein, X-linked | 8406 |
| SGIP1 | SGIP1 | SH3 domain GRB2 like endophilin interacting protein 1 | 84251 |
| TUBB6 | TUBB6 | tubulin beta 6 class V | 84617 |
| AFAP1L2 | AFAP1L2 | actin filament associated protein 1 like 2 | 84632 |
| ALKBH6 | ALKBH6 | alkB homolog 6 | 84964 |
| WISP1 | WISP1 | WNT1 inducible signaling pathway protein 1 | 8840 |
| SPHK1 | SPHK1 | sphingosine kinase 1 | 8877 |
| KLK4 | KLK4 | kallikrein related peptidase 4 | 9622 |
| NUAK1 | NUAK1 | NUAK family kinase 1 | 9891 |
| GFPT2 | GFPT2 | glutamine-fructose-6-phosphate transaminase 2 | 9945 |

#### User IDs mapped to multiple Entrtez IDs or not mapped

userid

C5ORF46

#### GOSlim summary for the user list genes

Each Biological Process, Cellular Component and Molecular Function category is represented by a red, blue and green bar, repectively.  
The height of the bar represents the number of user list genes observed in the category.

#### Detailed information of the enriched categories

The statistics
 **- C: the number of reference genes in the category
- O: the number of genes in the user gene list and also in the category
- E: The expected number in the category
- R: ratio of enrichment
- PValue: p value from hyergeometric test
- FDR: FDR from BH** 
for the enriched categories and the genes in the user gene list and also in the category are listed in the table.

- GO:0030198 extracellular matrix organization
- GO:0030574 collagen catabolic process
- GO:0043062 extracellular structure organization
- GO:0044243 multicellular organismal catabolic process
- GO:0032963 collagen metabolic process
- GO:0044259 multicellular organismal macromolecule metabolic process
- GO:0044236 multicellular organism metabolic process
- GO:0022610 biological adhesion
- GO:0072359 circulatory system development
- GO:0030199 collagen fibril organization

  

Download Table

| **ID:GO:0030198     &nbspName:extracellular matrix organization** | | | |
| --- | --- | --- | --- |
| C=328; O=34; E=2.24; R=15.18; PValue=0e+00; FDR=0e+00 | | | |
| userid | Gene Symbol | Gene Name | Entrez Gene |
| POSTN | POSTN | periostin | 10631 |
| COL1A1 | COL1A1 | collagen type I alpha 1 chain | 1277 |
| COL1A2 | COL1A2 | collagen type I alpha 2 chain | 1278 |
| COL3A1 | COL3A1 | collagen type III alpha 1 chain | 1281 |
| COL5A1 | COL5A1 | collagen type V alpha 1 chain | 1289 |
| COL5A2 | COL5A2 | collagen type V alpha 2 chain | 1290 |
| COL6A1 | COL6A1 | collagen type VI alpha 1 chain | 1291 |
| COL6A3 | COL6A3 | collagen type VI alpha 3 chain | 1293 |
| COL8A2 | COL8A2 | collagen type VIII alpha 2 chain | 1296 |
| COL10A1 | COL10A1 | collagen type X alpha 1 chain | 1300 |
| COL11A1 | COL11A1 | collagen type XI alpha 1 chain | 1301 |
| COL16A1 | COL16A1 | collagen type XVI alpha 1 chain | 1307 |
| VCAN | VCAN | versican | 1462 |
| CTSK | CTSK | cathepsin K | 1513 |
| DCN | DCN | decorin | 1634 |
| ECM2 | ECM2 | extracellular matrix protein 2 | 1842 |
| FBLN1 | FBLN1 | fibulin 1 | 2192 |
| FBN1 | FBN1 | fibrillin 1 | 2200 |
| ITGA11 | ITGA11 | integrin subunit alpha 11 | 22801 |
| PHLDB1 | PHLDB1 | pleckstrin homology like domain family B member 1 | 23187 |
| LAMA4 | LAMA4 | laminin subunit alpha 4 | 3910 |
| LUM | LUM | lumican | 4060 |
| MMP2 | MMP2 | matrix metallopeptidase 2 | 4313 |
| MMP11 | MMP11 | matrix metallopeptidase 11 | 4320 |
| MMP16 | MMP16 | matrix metallopeptidase 16 | 4325 |
| NID1 | NID1 | nidogen 1 | 4811 |
| HTRA1 | HTRA1 | HtrA serine peptidase 1 | 5654 |
| BGN | BGN | biglycan | 633 |
| SFRP2 | SFRP2 | secreted frizzled related protein 2 | 6423 |
| SPARC | SPARC | secreted protein acidic and cysteine rich | 6678 |
| TIMP2 | TIMP2 | TIMP metallopeptidase inhibitor 2 | 7077 |
| MFAP5 | MFAP5 | microfibrillar associated protein 5 | 8076 |
| JAM3 | JAM3 | junctional adhesion molecule 3 | 83700 |
| CRISPLD2 | CRISPLD2 | cysteine rich secretory protein LCCL domain containing 2 | 83716 |

  
  

Download Table

| **ID:GO:0030574     &nbspName:collagen catabolic process** | | | |
| --- | --- | --- | --- |
| C=67; O=14; E=0.46; R=30.61; PValue=0e+00; FDR=0e+00 | | | |
| userid | Gene Symbol | Gene Name | Entrez Gene |
| COL1A1 | COL1A1 | collagen type I alpha 1 chain | 1277 |
| COL1A2 | COL1A2 | collagen type I alpha 2 chain | 1278 |
| COL3A1 | COL3A1 | collagen type III alpha 1 chain | 1281 |
| COL5A1 | COL5A1 | collagen type V alpha 1 chain | 1289 |
| COL5A2 | COL5A2 | collagen type V alpha 2 chain | 1290 |
| COL6A1 | COL6A1 | collagen type VI alpha 1 chain | 1291 |
| COL6A3 | COL6A3 | collagen type VI alpha 3 chain | 1293 |
| COL8A2 | COL8A2 | collagen type VIII alpha 2 chain | 1296 |
| COL10A1 | COL10A1 | collagen type X alpha 1 chain | 1300 |
| COL11A1 | COL11A1 | collagen type XI alpha 1 chain | 1301 |
| CTSK | CTSK | cathepsin K | 1513 |
| MMP2 | MMP2 | matrix metallopeptidase 2 | 4313 |
| MMP11 | MMP11 | matrix metallopeptidase 11 | 4320 |
| MMP16 | MMP16 | matrix metallopeptidase 16 | 4325 |

  
  

Download Table

| **ID:GO:0043062     &nbspName:extracellular structure organization** | | | |
| --- | --- | --- | --- |
| C=329; O=34; E=2.25; R=15.14; PValue=0e+00; FDR=0e+00 | | | |
| userid | Gene Symbol | Gene Name | Entrez Gene |
| POSTN | POSTN | periostin | 10631 |
| COL1A1 | COL1A1 | collagen type I alpha 1 chain | 1277 |
| COL1A2 | COL1A2 | collagen type I alpha 2 chain | 1278 |
| COL3A1 | COL3A1 | collagen type III alpha 1 chain | 1281 |
| COL5A1 | COL5A1 | collagen type V alpha 1 chain | 1289 |
| COL5A2 | COL5A2 | collagen type V alpha 2 chain | 1290 |
| COL6A1 | COL6A1 | collagen type VI alpha 1 chain | 1291 |
| COL6A3 | COL6A3 | collagen type VI alpha 3 chain | 1293 |
| COL8A2 | COL8A2 | collagen type VIII alpha 2 chain | 1296 |
| COL10A1 | COL10A1 | collagen type X alpha 1 chain | 1300 |
| COL11A1 | COL11A1 | collagen type XI alpha 1 chain | 1301 |
| COL16A1 | COL16A1 | collagen type XVI alpha 1 chain | 1307 |
| VCAN | VCAN | versican | 1462 |
| CTSK | CTSK | cathepsin K | 1513 |
| DCN | DCN | decorin | 1634 |
| ECM2 | ECM2 | extracellular matrix protein 2 | 1842 |
| FBLN1 | FBLN1 | fibulin 1 | 2192 |
| FBN1 | FBN1 | fibrillin 1 | 2200 |
| ITGA11 | ITGA11 | integrin subunit alpha 11 | 22801 |
| PHLDB1 | PHLDB1 | pleckstrin homology like domain family B member 1 | 23187 |
| LAMA4 | LAMA4 | laminin subunit alpha 4 | 3910 |
| LUM | LUM | lumican | 4060 |
| MMP2 | MMP2 | matrix metallopeptidase 2 | 4313 |
| MMP11 | MMP11 | matrix metallopeptidase 11 | 4320 |
| MMP16 | MMP16 | matrix metallopeptidase 16 | 4325 |
| NID1 | NID1 | nidogen 1 | 4811 |
| HTRA1 | HTRA1 | HtrA serine peptidase 1 | 5654 |
| BGN | BGN | biglycan | 633 |
| SFRP2 | SFRP2 | secreted frizzled related protein 2 | 6423 |
| SPARC | SPARC | secreted protein acidic and cysteine rich | 6678 |
| TIMP2 | TIMP2 | TIMP metallopeptidase inhibitor 2 | 7077 |
| MFAP5 | MFAP5 | microfibrillar associated protein 5 | 8076 |
| JAM3 | JAM3 | junctional adhesion molecule 3 | 83700 |
| CRISPLD2 | CRISPLD2 | cysteine rich secretory protein LCCL domain containing 2 | 83716 |

  
  

Download Table

| **ID:GO:0044243     &nbspName:multicellular organismal catabolic process** | | | |
| --- | --- | --- | --- |
| C=72; O=14; E=0.49; R=28.48; PValue=0e+00; FDR=0e+00 | | | |
| userid | Gene Symbol | Gene Name | Entrez Gene |
| COL1A1 | COL1A1 | collagen type I alpha 1 chain | 1277 |
| COL1A2 | COL1A2 | collagen type I alpha 2 chain | 1278 |
| COL3A1 | COL3A1 | collagen type III alpha 1 chain | 1281 |
| COL5A1 | COL5A1 | collagen type V alpha 1 chain | 1289 |
| COL5A2 | COL5A2 | collagen type V alpha 2 chain | 1290 |
| COL6A1 | COL6A1 | collagen type VI alpha 1 chain | 1291 |
| COL6A3 | COL6A3 | collagen type VI alpha 3 chain | 1293 |
| COL8A2 | COL8A2 | collagen type VIII alpha 2 chain | 1296 |
| COL10A1 | COL10A1 | collagen type X alpha 1 chain | 1300 |
| COL11A1 | COL11A1 | collagen type XI alpha 1 chain | 1301 |
| CTSK | CTSK | cathepsin K | 1513 |
| MMP2 | MMP2 | matrix metallopeptidase 2 | 4313 |
| MMP11 | MMP11 | matrix metallopeptidase 11 | 4320 |
| MMP16 | MMP16 | matrix metallopeptidase 16 | 4325 |

  
  

Download Table

| **ID:GO:0032963     &nbspName:collagen metabolic process** | | | |
| --- | --- | --- | --- |
| C=109; O=15; E=0.74; R=20.16; PValue=6.66e-16; FDR=1.14e-12 | | | |
| userid | Gene Symbol | Gene Name | Entrez Gene |
| COL1A1 | COL1A1 | collagen type I alpha 1 chain | 1277 |
| COL1A2 | COL1A2 | collagen type I alpha 2 chain | 1278 |
| COL3A1 | COL3A1 | collagen type III alpha 1 chain | 1281 |
| COL5A1 | COL5A1 | collagen type V alpha 1 chain | 1289 |
| COL5A2 | COL5A2 | collagen type V alpha 2 chain | 1290 |
| COL6A1 | COL6A1 | collagen type VI alpha 1 chain | 1291 |
| COL6A3 | COL6A3 | collagen type VI alpha 3 chain | 1293 |
| COL8A2 | COL8A2 | collagen type VIII alpha 2 chain | 1296 |
| COL10A1 | COL10A1 | collagen type X alpha 1 chain | 1300 |
| COL11A1 | COL11A1 | collagen type XI alpha 1 chain | 1301 |
| CTSK | CTSK | cathepsin K | 1513 |
| MMP2 | MMP2 | matrix metallopeptidase 2 | 4313 |
| MMP11 | MMP11 | matrix metallopeptidase 11 | 4320 |
| MMP16 | MMP16 | matrix metallopeptidase 16 | 4325 |
| PDGFRB | PDGFRB | platelet derived growth factor receptor beta | 5159 |

  
  

Download Table

| **ID:GO:0044259     &nbspName:multicellular organismal macromolecule metabolic process** | | | |
| --- | --- | --- | --- |
| C=114; O=15; E=0.78; R=19.27; PValue=1.44e-15; FDR=2.06e-12 | | | |
| userid | Gene Symbol | Gene Name | Entrez Gene |
| COL1A1 | COL1A1 | collagen type I alpha 1 chain | 1277 |
| COL1A2 | COL1A2 | collagen type I alpha 2 chain | 1278 |
| COL3A1 | COL3A1 | collagen type III alpha 1 chain | 1281 |
| COL5A1 | COL5A1 | collagen type V alpha 1 chain | 1289 |
| COL5A2 | COL5A2 | collagen type V alpha 2 chain | 1290 |
| COL6A1 | COL6A1 | collagen type VI alpha 1 chain | 1291 |
| COL6A3 | COL6A3 | collagen type VI alpha 3 chain | 1293 |
| COL8A2 | COL8A2 | collagen type VIII alpha 2 chain | 1296 |
| COL10A1 | COL10A1 | collagen type X alpha 1 chain | 1300 |
| COL11A1 | COL11A1 | collagen type XI alpha 1 chain | 1301 |
| CTSK | CTSK | cathepsin K | 1513 |
| MMP2 | MMP2 | matrix metallopeptidase 2 | 4313 |
| MMP11 | MMP11 | matrix metallopeptidase 11 | 4320 |
| MMP16 | MMP16 | matrix metallopeptidase 16 | 4325 |
| PDGFRB | PDGFRB | platelet derived growth factor receptor beta | 5159 |

  
  

Download Table

| **ID:GO:0044236     &nbspName:multicellular organism metabolic process** | | | |
| --- | --- | --- | --- |
| C=129; O=15; E=0.88; R=17.03; PValue=9.66e-15; FDR=1.18e-11 | | | |
| userid | Gene Symbol | Gene Name | Entrez Gene |
| COL1A1 | COL1A1 | collagen type I alpha 1 chain | 1277 |
| COL1A2 | COL1A2 | collagen type I alpha 2 chain | 1278 |
| COL3A1 | COL3A1 | collagen type III alpha 1 chain | 1281 |
| COL5A1 | COL5A1 | collagen type V alpha 1 chain | 1289 |
| COL5A2 | COL5A2 | collagen type V alpha 2 chain | 1290 |
| COL6A1 | COL6A1 | collagen type VI alpha 1 chain | 1291 |
| COL6A3 | COL6A3 | collagen type VI alpha 3 chain | 1293 |
| COL8A2 | COL8A2 | collagen type VIII alpha 2 chain | 1296 |
| COL10A1 | COL10A1 | collagen type X alpha 1 chain | 1300 |
| COL11A1 | COL11A1 | collagen type XI alpha 1 chain | 1301 |
| CTSK | CTSK | cathepsin K | 1513 |
| MMP2 | MMP2 | matrix metallopeptidase 2 | 4313 |
| MMP11 | MMP11 | matrix metallopeptidase 11 | 4320 |
| MMP16 | MMP16 | matrix metallopeptidase 16 | 4325 |
| PDGFRB | PDGFRB | platelet derived growth factor receptor beta | 5159 |

  
  

Download Table

| **ID:GO:0022610     &nbspName:biological adhesion** | | | |
| --- | --- | --- | --- |
| C=1673; O=39; E=11.42; R=3.41; PValue=1.7e-12; FDR=1.81e-09 | | | |
| userid | Gene Symbol | Gene Name | Entrez Gene |
| CDH11 | CDH11 | cadherin 11 | 1009 |
| MYL9 | MYL9 | myosin light chain 9 | 10398 |
| SPON1 | SPON1 | spondin 1 | 10418 |
| POSTN | POSTN | periostin | 10631 |
| FERMT2 | FERMT2 | fermitin family member 2 | 10979 |
| EMILIN1 | EMILIN1 | elastin microfibril interfacer 1 | 11117 |
| COL1A1 | COL1A1 | collagen type I alpha 1 chain | 1277 |
| COL3A1 | COL3A1 | collagen type III alpha 1 chain | 1281 |
| COL5A1 | COL5A1 | collagen type V alpha 1 chain | 1289 |
| COL6A1 | COL6A1 | collagen type VI alpha 1 chain | 1291 |
| COL6A3 | COL6A3 | collagen type VI alpha 3 chain | 1293 |
| COL8A2 | COL8A2 | collagen type VIII alpha 2 chain | 1296 |
| COL16A1 | COL16A1 | collagen type XVI alpha 1 chain | 1307 |
| LRRC15 | LRRC15 | leucine rich repeat containing 15 | 131578 |
| VCAN | VCAN | versican | 1462 |
| CD55 | CD55 | CD55 molecule (Cromer blood group) | 1604 |
| ECM2 | ECM2 | extracellular matrix protein 2 | 1842 |
| FBLN1 | FBLN1 | fibulin 1 | 2192 |
| FBN1 | FBN1 | fibrillin 1 | 2200 |
| ITGA11 | ITGA11 | integrin subunit alpha 11 | 22801 |
| LRRC32 | LRRC32 | leucine rich repeat containing 32 | 2615 |
| SRPX2 | SRPX2 | sushi repeat containing protein, X-linked 2 | 27286 |
| LAMA4 | LAMA4 | laminin subunit alpha 4 | 3910 |
| NID1 | NID1 | nidogen 1 | 4811 |
| OMD | OMD | osteomodulin | 4958 |
| TENM3 | TENM3 | teneurin transmembrane protein 3 | 55714 |
| AXL | AXL | AXL receptor tyrosine kinase | 558 |
| SFRP2 | SFRP2 | secreted frizzled related protein 2 | 6423 |
| SNAI2 | SNAI2 | snail family transcriptional repressor 2 | 6591 |
| SPOCK1 | SPOCK1 | SPARC/osteonectin, cwcv and kazal like domains proteoglycan 1 | 6695 |
| TGFB1I1 | TGFB1I1 | transforming growth factor beta 1 induced transcript 1 | 7041 |
| THBS2 | THBS2 | thrombospondin 2 | 7058 |
| THY1 | THY1 | Thy-1 cell surface antigen | 7070 |
| TRO | TRO | trophinin | 7216 |
| TMEM47 | TMEM47 | transmembrane protein 47 | 83604 |
| JAM3 | JAM3 | junctional adhesion molecule 3 | 83700 |
| SRPX | SRPX | sushi repeat containing protein, X-linked | 8406 |
| WISP1 | WISP1 | WNT1 inducible signaling pathway protein 1 | 8840 |
| NUAK1 | NUAK1 | NUAK family kinase 1 | 9891 |

  
  

Download Table

| **ID:GO:0072359     &nbspName:circulatory system development** | | | |
| --- | --- | --- | --- |
| C=920; O=29; E=6.28; R=4.62; PValue=2.13e-12; FDR=2.02e-09 | | | |
| userid | Gene Symbol | Gene Name | Entrez Gene |
| COL1A1 | COL1A1 | collagen type I alpha 1 chain | 1277 |
| COL1A2 | COL1A2 | collagen type I alpha 2 chain | 1278 |
| COL3A1 | COL3A1 | collagen type III alpha 1 chain | 1281 |
| COL5A1 | COL5A1 | collagen type V alpha 1 chain | 1289 |
| COL8A2 | COL8A2 | collagen type VIII alpha 2 chain | 1296 |
| COL11A1 | COL11A1 | collagen type XI alpha 1 chain | 1301 |
| CSPG4 | CSPG4 | chondroitin sulfate proteoglycan 4 | 1464 |
| DCN | DCN | decorin | 1634 |
| EDNRA | EDNRA | endothelin receptor type A | 1909 |
| FBN1 | FBN1 | fibrillin 1 | 2200 |
| HEYL | HEYL | hes related family bHLH transcription factor with YRPW motif-like | 26508 |
| GJA1 | GJA1 | gap junction protein alpha 1 | 2697 |
| SRPX2 | SRPX2 | sushi repeat containing protein, X-linked 2 | 27286 |
| MMP2 | MMP2 | matrix metallopeptidase 2 | 4313 |
| PAM | PAM | peptidylglycine alpha-amidating monooxygenase | 5066 |
| PDGFRB | PDGFRB | platelet derived growth factor receptor beta | 5159 |
| SERPINF1 | SERPINF1 | serpin family F member 1 | 5176 |
| PKD2 | PKD2 | polycystin 2, transient receptor potential cation channel | 5311 |
| ACTA2 | ACTA2 | actin, alpha 2, smooth muscle, aorta | 59 |
| SFRP2 | SFRP2 | secreted frizzled related protein 2 | 6423 |
| SGCD | SGCD | sarcoglycan delta | 6444 |
| SNAI2 | SNAI2 | snail family transcriptional repressor 2 | 6591 |
| SPARC | SPARC | secreted protein acidic and cysteine rich | 6678 |
| THBS2 | THBS2 | thrombospondin 2 | 7058 |
| THY1 | THY1 | Thy-1 cell surface antigen | 7070 |
| BICC1 | BICC1 | BicC family RNA binding protein 1 | 80114 |
| FHOD3 | FHOD3 | formin homology 2 domain containing 3 | 80206 |
| JAM3 | JAM3 | junctional adhesion molecule 3 | 83700 |
| SPHK1 | SPHK1 | sphingosine kinase 1 | 8877 |

  
  

Download Table

| **ID:GO:0030199     &nbspName:collagen fibril organization** | | | |
| --- | --- | --- | --- |
| C=38; O=9; E=0.26; R=34.69; PValue=3.2e-12; FDR=2.74e-09 | | | |
| userid | Gene Symbol | Gene Name | Entrez Gene |
| COL1A1 | COL1A1 | collagen type I alpha 1 chain | 1277 |
| COL1A2 | COL1A2 | collagen type I alpha 2 chain | 1278 |
| COL3A1 | COL3A1 | collagen type III alpha 1 chain | 1281 |
| COL5A1 | COL5A1 | collagen type V alpha 1 chain | 1289 |
| COL5A2 | COL5A2 | collagen type V alpha 2 chain | 1290 |
| COL11A1 | COL11A1 | collagen type XI alpha 1 chain | 1301 |
| LUM | LUM | lumican | 4060 |
| MMP11 | MMP11 | matrix metallopeptidase 11 | 4320 |
| SFRP2 | SFRP2 | secreted frizzled related protein 2 | 6423 |
