# Supplementary material for: The Hierarchical Modular Structure of HER2+ Breast Cancer Network
Source: Front Physiol. 2018 Oct 11;9:1423. doi: 10.3389/fphys.2018.01423 (PMC6193406; doi:10.3389/fphys.2018.01423)
Supplement: Supplementary file 1 [file Data_Sheet_1.zip › EnrichmentResults.zip!/EnrichmentViralResponse.gz!/./Report_wg_result1531782380.html]

WebGestalt (WEB-based GEne SeT AnaLysis Toolkit)


- Summary
- User ID Mapping Table
- GOSlim Summary
- Enrichment Results

### Summary   (Result Download)

**Enrich method:** ORA  
**Organism:**hsapiens  
**Enrichment Categories:** geneontology\_Biological\_Process  
**Interesting gene list:** textAreaUpload\_1531782380.txt. **ID type:** genesymbol  
The interesting gene list contains **25** user IDs in which **25** user IDs are unambiguously mapped to the unique Entrez Gene IDs and **0** user IDs are mapped to multiple Entrez Gene IDs or could not be mapped to any Entrez Gene ID. The GO Slim summary are based upon the **25** unique Entrez Gene IDs.  
Among the **25** unique Entrez Gene IDs, **23** IDs are annotated to the selected functional categories and also in the reference gene list, which are used for the enrichment analysis.  
**Reference gene list:**  all mapped Entrez Gene IDs from the selected platform genome  
The reference gene list contains **60119** IDs and  **16258** IDs are annotated to the selected functional categories that are used as the reference for the enrichment analysis.  
**Parameters for the enrichment analysis:**

- **Minimum number of Entrez Gene IDs in the category:**5
- **Maximum number of Entrez Gene IDs in the category:**2000
- **FDR Method:**BH
- **Significance Level:** Top10

Based on the above parameters, **10** categories are identified as enriched categories and all are shown in this report.

#### Mapped User IDs

| userid | Gene Symbol | Gene Name | Entrez Gene |
| --- | --- | --- | --- |
| IFI44 | IFI44 | interferon induced protein 44 | 10561 |
| IFI44L | IFI44L | interferon induced protein 44 like | 10964 |
| TDRD7 | TDRD7 | tudor domain containing 7 | 23424 |
| IFI27 | IFI27 | interferon alpha inducible protein 27 | 3429 |
| IFI35 | IFI35 | interferon induced protein 35 | 3430 |
| IFIT1 | IFIT1 | interferon induced protein with tetratricopeptide repeats 1 | 3434 |
| IRF7 | IRF7 | interferon regulatory factor 7 | 3665 |
| MAFA | MAFA | MAF bZIP transcription factor A | 389692 |
| MX1 | MX1 | MX dynamin like GTPase 1 | 4599 |
| MX2 | MX2 | MX dynamin like GTPase 2 | 4600 |
| OAS1 | OAS1 | 2'-5'-oligoadenylate synthetase 1 | 4938 |
| OAS2 | OAS2 | 2'-5'-oligoadenylate synthetase 2 | 4939 |
| OAS3 | OAS3 | 2'-5'-oligoadenylate synthetase 3 | 4940 |
| HERC5 | HERC5 | HECT and RLD domain containing E3 ubiquitin protein ligase 5 | 51191 |
| PARP14 | PARP14 | poly(ADP-ribose) polymerase family member 14 | 54625 |
| SAMD9 | SAMD9 | sterile alpha motif domain containing 9 | 54809 |
| HERC6 | HERC6 | HECT and RLD domain containing E3 ubiquitin protein ligase family member 6 | 55008 |
| DDX60 | DDX60 | DExD/H-box helicase 60 | 55601 |
| ZNFX1 | ZNFX1 | zinc finger NFX1-type containing 1 | 57169 |
| RTP4 | RTP4 | receptor transporter protein 4 | 64108 |
| PARP12 | PARP12 | poly(ADP-ribose) polymerase family member 12 | 64761 |
| IFITM1 | IFITM1 | interferon induced transmembrane protein 1 | 8519 |
| OASL | OASL | 2'-5'-oligoadenylate synthetase like | 8638 |
| RSAD2 | RSAD2 | radical S-adenosyl methionine domain containing 2 | 91543 |
| ISG15 | ISG15 | ISG15 ubiquitin-like modifier | 9636 |

#### User IDs mapped to multiple Entrtez IDs or not mapped

userid

NA

#### GOSlim summary for the user list genes

Each Biological Process, Cellular Component and Molecular Function category is represented by a red, blue and green bar, repectively.  
The height of the bar represents the number of user list genes observed in the category.

#### Detailed information of the enriched categories

The statistics
 **- C: the number of reference genes in the category
- O: the number of genes in the user gene list and also in the category
- E: The expected number in the category
- R: ratio of enrichment
- PValue: p value from hyergeometric test
- FDR: FDR from BH** 
for the enriched categories and the genes in the user gene list and also in the category are listed in the table.

- GO:0009615 response to virus
- GO:0034340 response to type I interferon
- GO:0051607 defense response to virus
- GO:0060337 type I interferon signaling pathway
- GO:0071357 cellular response to type I interferon
- GO:0098542 defense response to other organism
- GO:0043207 response to external biotic stimulus
- GO:0051707 response to other organism
- GO:0045087 innate immune response
- GO:0045071 negative regulation of viral genome replication

  

Download Table

| **ID:GO:0009615     &nbspName:response to virus** | | | |
| --- | --- | --- | --- |
| C=304; O=15; E=0.39; R=38.2; PValue=0e+00; FDR=0e+00 | | | |
| userid | Gene Symbol | Gene Name | Entrez Gene |
| IFI44 | IFI44 | interferon induced protein 44 | 10561 |
| IFI44L | IFI44L | interferon induced protein 44 like | 10964 |
| IFIT1 | IFIT1 | interferon induced protein with tetratricopeptide repeats 1 | 3434 |
| IRF7 | IRF7 | interferon regulatory factor 7 | 3665 |
| MX1 | MX1 | MX dynamin like GTPase 1 | 4599 |
| MX2 | MX2 | MX dynamin like GTPase 2 | 4600 |
| OAS1 | OAS1 | 2'-5'-oligoadenylate synthetase 1 | 4938 |
| OAS2 | OAS2 | 2'-5'-oligoadenylate synthetase 2 | 4939 |
| OAS3 | OAS3 | 2'-5'-oligoadenylate synthetase 3 | 4940 |
| HERC5 | HERC5 | HECT and RLD domain containing E3 ubiquitin protein ligase 5 | 51191 |
| DDX60 | DDX60 | DExD/H-box helicase 60 | 55601 |
| IFITM1 | IFITM1 | interferon induced transmembrane protein 1 | 8519 |
| OASL | OASL | 2'-5'-oligoadenylate synthetase like | 8638 |
| RSAD2 | RSAD2 | radical S-adenosyl methionine domain containing 2 | 91543 |
| ISG15 | ISG15 | ISG15 ubiquitin-like modifier | 9636 |

  
  

Download Table

| **ID:GO:0034340     &nbspName:response to type I interferon** | | | |
| --- | --- | --- | --- |
| C=79; O=13; E=0.1; R=127.4; PValue=0e+00; FDR=0e+00 | | | |
| userid | Gene Symbol | Gene Name | Entrez Gene |
| IFI27 | IFI27 | interferon alpha inducible protein 27 | 3429 |
| IFI35 | IFI35 | interferon induced protein 35 | 3430 |
| IFIT1 | IFIT1 | interferon induced protein with tetratricopeptide repeats 1 | 3434 |
| IRF7 | IRF7 | interferon regulatory factor 7 | 3665 |
| MX1 | MX1 | MX dynamin like GTPase 1 | 4599 |
| MX2 | MX2 | MX dynamin like GTPase 2 | 4600 |
| OAS1 | OAS1 | 2'-5'-oligoadenylate synthetase 1 | 4938 |
| OAS2 | OAS2 | 2'-5'-oligoadenylate synthetase 2 | 4939 |
| OAS3 | OAS3 | 2'-5'-oligoadenylate synthetase 3 | 4940 |
| IFITM1 | IFITM1 | interferon induced transmembrane protein 1 | 8519 |
| OASL | OASL | 2'-5'-oligoadenylate synthetase like | 8638 |
| RSAD2 | RSAD2 | radical S-adenosyl methionine domain containing 2 | 91543 |
| ISG15 | ISG15 | ISG15 ubiquitin-like modifier | 9636 |

  
  

Download Table

| **ID:GO:0051607     &nbspName:defense response to virus** | | | |
| --- | --- | --- | --- |
| C=227; O=14; E=0.29; R=47.75; PValue=0e+00; FDR=0e+00 | | | |
| userid | Gene Symbol | Gene Name | Entrez Gene |
| IFI44L | IFI44L | interferon induced protein 44 like | 10964 |
| IFIT1 | IFIT1 | interferon induced protein with tetratricopeptide repeats 1 | 3434 |
| IRF7 | IRF7 | interferon regulatory factor 7 | 3665 |
| MX1 | MX1 | MX dynamin like GTPase 1 | 4599 |
| MX2 | MX2 | MX dynamin like GTPase 2 | 4600 |
| OAS1 | OAS1 | 2'-5'-oligoadenylate synthetase 1 | 4938 |
| OAS2 | OAS2 | 2'-5'-oligoadenylate synthetase 2 | 4939 |
| OAS3 | OAS3 | 2'-5'-oligoadenylate synthetase 3 | 4940 |
| HERC5 | HERC5 | HECT and RLD domain containing E3 ubiquitin protein ligase 5 | 51191 |
| DDX60 | DDX60 | DExD/H-box helicase 60 | 55601 |
| IFITM1 | IFITM1 | interferon induced transmembrane protein 1 | 8519 |
| OASL | OASL | 2'-5'-oligoadenylate synthetase like | 8638 |
| RSAD2 | RSAD2 | radical S-adenosyl methionine domain containing 2 | 91543 |
| ISG15 | ISG15 | ISG15 ubiquitin-like modifier | 9636 |

  
  

Download Table

| **ID:GO:0060337     &nbspName:type I interferon signaling pathway** | | | |
| --- | --- | --- | --- |
| C=75; O=13; E=0.1; R=134.19; PValue=0e+00; FDR=0e+00 | | | |
| userid | Gene Symbol | Gene Name | Entrez Gene |
| IFI27 | IFI27 | interferon alpha inducible protein 27 | 3429 |
| IFI35 | IFI35 | interferon induced protein 35 | 3430 |
| IFIT1 | IFIT1 | interferon induced protein with tetratricopeptide repeats 1 | 3434 |
| IRF7 | IRF7 | interferon regulatory factor 7 | 3665 |
| MX1 | MX1 | MX dynamin like GTPase 1 | 4599 |
| MX2 | MX2 | MX dynamin like GTPase 2 | 4600 |
| OAS1 | OAS1 | 2'-5'-oligoadenylate synthetase 1 | 4938 |
| OAS2 | OAS2 | 2'-5'-oligoadenylate synthetase 2 | 4939 |
| OAS3 | OAS3 | 2'-5'-oligoadenylate synthetase 3 | 4940 |
| IFITM1 | IFITM1 | interferon induced transmembrane protein 1 | 8519 |
| OASL | OASL | 2'-5'-oligoadenylate synthetase like | 8638 |
| RSAD2 | RSAD2 | radical S-adenosyl methionine domain containing 2 | 91543 |
| ISG15 | ISG15 | ISG15 ubiquitin-like modifier | 9636 |

  
  

Download Table

| **ID:GO:0071357     &nbspName:cellular response to type I interferon** | | | |
| --- | --- | --- | --- |
| C=75; O=13; E=0.1; R=134.19; PValue=0e+00; FDR=0e+00 | | | |
| userid | Gene Symbol | Gene Name | Entrez Gene |
| IFI27 | IFI27 | interferon alpha inducible protein 27 | 3429 |
| IFI35 | IFI35 | interferon induced protein 35 | 3430 |
| IFIT1 | IFIT1 | interferon induced protein with tetratricopeptide repeats 1 | 3434 |
| IRF7 | IRF7 | interferon regulatory factor 7 | 3665 |
| MX1 | MX1 | MX dynamin like GTPase 1 | 4599 |
| MX2 | MX2 | MX dynamin like GTPase 2 | 4600 |
| OAS1 | OAS1 | 2'-5'-oligoadenylate synthetase 1 | 4938 |
| OAS2 | OAS2 | 2'-5'-oligoadenylate synthetase 2 | 4939 |
| OAS3 | OAS3 | 2'-5'-oligoadenylate synthetase 3 | 4940 |
| IFITM1 | IFITM1 | interferon induced transmembrane protein 1 | 8519 |
| OASL | OASL | 2'-5'-oligoadenylate synthetase like | 8638 |
| RSAD2 | RSAD2 | radical S-adenosyl methionine domain containing 2 | 91543 |
| ISG15 | ISG15 | ISG15 ubiquitin-like modifier | 9636 |

  
  

Download Table

| **ID:GO:0098542     &nbspName:defense response to other organism** | | | |
| --- | --- | --- | --- |
| C=443; O=14; E=0.57; R=24.47; PValue=0e+00; FDR=0e+00 | | | |
| userid | Gene Symbol | Gene Name | Entrez Gene |
| IFI44L | IFI44L | interferon induced protein 44 like | 10964 |
| IFIT1 | IFIT1 | interferon induced protein with tetratricopeptide repeats 1 | 3434 |
| IRF7 | IRF7 | interferon regulatory factor 7 | 3665 |
| MX1 | MX1 | MX dynamin like GTPase 1 | 4599 |
| MX2 | MX2 | MX dynamin like GTPase 2 | 4600 |
| OAS1 | OAS1 | 2'-5'-oligoadenylate synthetase 1 | 4938 |
| OAS2 | OAS2 | 2'-5'-oligoadenylate synthetase 2 | 4939 |
| OAS3 | OAS3 | 2'-5'-oligoadenylate synthetase 3 | 4940 |
| HERC5 | HERC5 | HECT and RLD domain containing E3 ubiquitin protein ligase 5 | 51191 |
| DDX60 | DDX60 | DExD/H-box helicase 60 | 55601 |
| IFITM1 | IFITM1 | interferon induced transmembrane protein 1 | 8519 |
| OASL | OASL | 2'-5'-oligoadenylate synthetase like | 8638 |
| RSAD2 | RSAD2 | radical S-adenosyl methionine domain containing 2 | 91543 |
| ISG15 | ISG15 | ISG15 ubiquitin-like modifier | 9636 |

  
  

Download Table

| **ID:GO:0043207     &nbspName:response to external biotic stimulus** | | | |
| --- | --- | --- | --- |
| C=771; O=15; E=1; R=15.06; PValue=4.44e-16; FDR=4.75e-13 | | | |
| userid | Gene Symbol | Gene Name | Entrez Gene |
| IFI44 | IFI44 | interferon induced protein 44 | 10561 |
| IFI44L | IFI44L | interferon induced protein 44 like | 10964 |
| IFIT1 | IFIT1 | interferon induced protein with tetratricopeptide repeats 1 | 3434 |
| IRF7 | IRF7 | interferon regulatory factor 7 | 3665 |
| MX1 | MX1 | MX dynamin like GTPase 1 | 4599 |
| MX2 | MX2 | MX dynamin like GTPase 2 | 4600 |
| OAS1 | OAS1 | 2'-5'-oligoadenylate synthetase 1 | 4938 |
| OAS2 | OAS2 | 2'-5'-oligoadenylate synthetase 2 | 4939 |
| OAS3 | OAS3 | 2'-5'-oligoadenylate synthetase 3 | 4940 |
| HERC5 | HERC5 | HECT and RLD domain containing E3 ubiquitin protein ligase 5 | 51191 |
| DDX60 | DDX60 | DExD/H-box helicase 60 | 55601 |
| IFITM1 | IFITM1 | interferon induced transmembrane protein 1 | 8519 |
| OASL | OASL | 2'-5'-oligoadenylate synthetase like | 8638 |
| RSAD2 | RSAD2 | radical S-adenosyl methionine domain containing 2 | 91543 |
| ISG15 | ISG15 | ISG15 ubiquitin-like modifier | 9636 |

  
  

Download Table

| **ID:GO:0051707     &nbspName:response to other organism** | | | |
| --- | --- | --- | --- |
| C=770; O=15; E=0.99; R=15.08; PValue=4.44e-16; FDR=4.75e-13 | | | |
| userid | Gene Symbol | Gene Name | Entrez Gene |
| IFI44 | IFI44 | interferon induced protein 44 | 10561 |
| IFI44L | IFI44L | interferon induced protein 44 like | 10964 |
| IFIT1 | IFIT1 | interferon induced protein with tetratricopeptide repeats 1 | 3434 |
| IRF7 | IRF7 | interferon regulatory factor 7 | 3665 |
| MX1 | MX1 | MX dynamin like GTPase 1 | 4599 |
| MX2 | MX2 | MX dynamin like GTPase 2 | 4600 |
| OAS1 | OAS1 | 2'-5'-oligoadenylate synthetase 1 | 4938 |
| OAS2 | OAS2 | 2'-5'-oligoadenylate synthetase 2 | 4939 |
| OAS3 | OAS3 | 2'-5'-oligoadenylate synthetase 3 | 4940 |
| HERC5 | HERC5 | HECT and RLD domain containing E3 ubiquitin protein ligase 5 | 51191 |
| DDX60 | DDX60 | DExD/H-box helicase 60 | 55601 |
| IFITM1 | IFITM1 | interferon induced transmembrane protein 1 | 8519 |
| OASL | OASL | 2'-5'-oligoadenylate synthetase like | 8638 |
| RSAD2 | RSAD2 | radical S-adenosyl methionine domain containing 2 | 91543 |
| ISG15 | ISG15 | ISG15 ubiquitin-like modifier | 9636 |

  
  

Download Table

| **ID:GO:0045087     &nbspName:innate immune response** | | | |
| --- | --- | --- | --- |
| C=775; O=15; E=1; R=14.98; PValue=5.55e-16; FDR=5.27e-13 | | | |
| userid | Gene Symbol | Gene Name | Entrez Gene |
| IFI27 | IFI27 | interferon alpha inducible protein 27 | 3429 |
| IFI35 | IFI35 | interferon induced protein 35 | 3430 |
| IFIT1 | IFIT1 | interferon induced protein with tetratricopeptide repeats 1 | 3434 |
| IRF7 | IRF7 | interferon regulatory factor 7 | 3665 |
| MX1 | MX1 | MX dynamin like GTPase 1 | 4599 |
| MX2 | MX2 | MX dynamin like GTPase 2 | 4600 |
| OAS1 | OAS1 | 2'-5'-oligoadenylate synthetase 1 | 4938 |
| OAS2 | OAS2 | 2'-5'-oligoadenylate synthetase 2 | 4939 |
| OAS3 | OAS3 | 2'-5'-oligoadenylate synthetase 3 | 4940 |
| HERC5 | HERC5 | HECT and RLD domain containing E3 ubiquitin protein ligase 5 | 51191 |
| DDX60 | DDX60 | DExD/H-box helicase 60 | 55601 |
| IFITM1 | IFITM1 | interferon induced transmembrane protein 1 | 8519 |
| OASL | OASL | 2'-5'-oligoadenylate synthetase like | 8638 |
| RSAD2 | RSAD2 | radical S-adenosyl methionine domain containing 2 | 91543 |
| ISG15 | ISG15 | ISG15 ubiquitin-like modifier | 9636 |

  
  

Download Table

| **ID:GO:0045071     &nbspName:negative regulation of viral genome replication** | | | |
| --- | --- | --- | --- |
| C=48; O=8; E=0.06; R=129.03; PValue=6.66e-16; FDR=5.69e-13 | | | |
| userid | Gene Symbol | Gene Name | Entrez Gene |
| IFIT1 | IFIT1 | interferon induced protein with tetratricopeptide repeats 1 | 3434 |
| MX1 | MX1 | MX dynamin like GTPase 1 | 4599 |
| OAS1 | OAS1 | 2'-5'-oligoadenylate synthetase 1 | 4938 |
| OAS3 | OAS3 | 2'-5'-oligoadenylate synthetase 3 | 4940 |
| IFITM1 | IFITM1 | interferon induced transmembrane protein 1 | 8519 |
| OASL | OASL | 2'-5'-oligoadenylate synthetase like | 8638 |
| RSAD2 | RSAD2 | radical S-adenosyl methionine domain containing 2 | 91543 |
| ISG15 | ISG15 | ISG15 ubiquitin-like modifier | 9636 |
