# Supplementary material for: The Hierarchical Modular Structure of HER2+ Breast Cancer Network
Source: Front Physiol. 2018 Oct 11;9:1423. doi: 10.3389/fphys.2018.01423 (PMC6193406; doi:10.3389/fphys.2018.01423)
Supplement: Supplementary file 1 [file Data_Sheet_1.zip › EnrichmentResults.zip!/LargestComponent_ImmunityValidation.gz!/./Report_wg_result1531779839.html]

WebGestalt (WEB-based GEne SeT AnaLysis Toolkit)


- Summary
- User ID Mapping Table
- GOSlim Summary
- Enrichment Results

### Summary   (Result Download)

**Enrich method:** ORA  
**Organism:**hsapiens  
**Enrichment Categories:** geneontology\_Biological\_Process  
**Interesting gene list:** textAreaUpload\_1531779839.txt. **ID type:** genesymbol  
The interesting gene list contains **346** user IDs in which **343** user IDs are unambiguously mapped to the unique Entrez Gene IDs and **3** user IDs are mapped to multiple Entrez Gene IDs or could not be mapped to any Entrez Gene ID. The GO Slim summary are based upon the **343** unique Entrez Gene IDs.  
Among the **343** unique Entrez Gene IDs, **305** IDs are annotated to the selected functional categories and also in the reference gene list, which are used for the enrichment analysis.  
**Reference gene list:**  all mapped Entrez Gene IDs from the selected platform genome  
The reference gene list contains **60119** IDs and  **16258** IDs are annotated to the selected functional categories that are used as the reference for the enrichment analysis.  
**Parameters for the enrichment analysis:**

- **Minimum number of Entrez Gene IDs in the category:**5
- **Maximum number of Entrez Gene IDs in the category:**2000
- **FDR Method:**BH
- **Significance Level:** FDR<1e-05

Based on the above parameters, **334** categories are identified as enriched categories, in which **40** most significant categories are shown in this report. All significant categories can be downloaded from the 'Result Download' link.

#### Mapped User IDs

| userid | Gene Symbol | Gene Name | Entrez Gene |
| --- | --- | --- | --- |
| SH2D3C | SH2D3C | SH2 domain containing 3C | 10044 |
| RASGRP1 | RASGRP1 | RAS guanyl releasing protein 1 | 10125 |
| EBI3 | EBI3 | Epstein-Barr virus induced 3 | 10148 |
| KLRG1 | KLRG1 | killer cell lectin like receptor G1 | 10219 |
| CD96 | CD96 | CD96 molecule | 10225 |
| IGSF6 | IGSF6 | immunoglobulin superfamily member 6 | 10261 |
| LILRB2 | LILRB2 | leukocyte immunoglobulin like receptor B2 | 10288 |
| IKZF1 | IKZF1 | IKAROS family zinc finger 1 | 10320 |
| TRIM22 | TRIM22 | tripartite motif containing 22 | 10346 |
| BTN3A3 | BTN3A3 | butyrophilin subfamily 3 member A3 | 10384 |
| IFITM3 | IFITM3 | interferon induced transmembrane protein 3 | 10410 |
| CD52 | CD52 | CD52 molecule | 1043 |
| CLEC10A | CLEC10A | C-type lectin domain family 10 member A | 10462 |
| SEMA4D | SEMA4D | semaphorin 4D | 10507 |
| IFI44 | IFI44 | interferon induced protein 44 | 10561 |
| CXCL13 | CXCL13 | C-X-C motif chemokine ligand 13 | 10563 |
| GNLY | GNLY | granulysin | 10578 |
| IFITM2 | IFITM2 | interferon induced transmembrane protein 2 | 10581 |
| CELF2 | CELF2 | CUGBP Elav-like family member 2 | 10659 |
| CXCR6 | CXCR6 | C-X-C motif chemokine receptor 6 | 10663 |
| TNFSF13B | TNFSF13B | tumor necrosis factor superfamily member 13b | 10673 |
| CTSC | CTSC | cathepsin C | 1075 |
| HCP5 | HCP5 | HLA complex P5 (non-protein coding) | 10866 |
| HCST | HCST | hematopoietic cell signal transducer | 10870 |
| FGL2 | FGL2 | fibrinogen like 2 | 10875 |
| IFI44L | IFI44L | interferon induced protein 44 like | 10964 |
| LILRB4 | LILRB4 | leukocyte immunoglobulin like receptor B4 | 11006 |
| GLIPR1 | GLIPR1 | GLI pathogenesis related 1 | 11010 |
| PIM2 | PIM2 | Pim-2 proto-oncogene, serine/threonine kinase | 11040 |
| BTN3A1 | BTN3A1 | butyrophilin subfamily 3 member A1 | 11119 |
| CORO1A | CORO1A | coronin 1A | 11151 |
| MAP4K1 | MAP4K1 | mitogen-activated protein kinase kinase kinase kinase 1 | 11184 |
| SP140 | SP140 | SP140 nuclear body protein | 11262 |
| SLCO2B1 | SLCO2B1 | solute carrier organic anion transporter family member 2B1 | 11309 |
| NLRP3 | NLRP3 | NLR family pyrin domain containing 3 | 114548 |
| CARD16 | CARD16 | caspase recruitment domain family member 16 | 114769 |
| BATF2 | BATF2 | basic leucine zipper ATF-like transcription factor 2 | 116071 |
| TAGAP | TAGAP | T-cell activation RhoGTPase activating protein | 117289 |
| CLIC2 | CLIC2 | chloride intracellular channel 2 | 1193 |
| CCR1 | CCR1 | C-C motif chemokine receptor 1 | 1230 |
| CCR7 | CCR7 | C-C motif chemokine receptor 7 | 1236 |
| OSCAR | OSCAR | osteoclast associated, immunoglobulin-like receptor | 126014 |
| ZNF831 | ZNF831 | zinc finger protein 831 | 128611 |
| TMEM71 | TMEM71 | transmembrane protein 71 | 137835 |
| DOCK11 | DOCK11 | dedicator of cytokinesis 11 | 139818 |
| TRIM69 | TRIM69 | tripartite motif containing 69 | 140691 |
| SIRPA | SIRPA | signal regulatory protein alpha | 140885 |
| CSF1R | CSF1R | colony stimulating factor 1 receptor | 1436 |
| HAPLN3 | HAPLN3 | hyaluronan and proteoglycan link protein 3 | 145864 |
| PYHIN1 | PYHIN1 | pyrin and HIN domain family member 1 | 149628 |
| CTSS | CTSS | cathepsin S | 1520 |
| CTSW | CTSW | cathepsin W | 1521 |
| DOCK2 | DOCK2 | dedicator of cytokinesis 2 | 1794 |
| DPYD | DPYD | dihydropyrimidine dehydrogenase | 1806 |
| GPR183 | GPR183 | G protein-coupled receptor 183 | 1880 |
| S1PR1 | S1PR1 | sphingosine-1-phosphate receptor 1 | 1901 |
| PATL2 | PATL2 | PAT1 homolog 2 | 197135 |
| AIF1 | AIF1 | allograft inflammatory factor 1 | 199 |
| ETS1 | ETS1 | ETS proto-oncogene 1, transcription factor | 2113 |
| EVI2A | EVI2A | ecotropic viral integration site 2A | 2123 |
| EVI2B | EVI2B | ecotropic viral integration site 2B | 2124 |
| ACSL4 | ACSL4 | acyl-CoA synthetase long-chain family member 4 | 2182 |
| FCN1 | FCN1 | ficolin 1 | 2219 |
| SYT11 | SYT11 | synaptotagmin 11 | 23208 |
| PLCL2 | PLCL2 | phospholipase C like 2 | 23228 |
| FLT3LG | FLT3LG | fms related tyrosine kinase 3 ligand | 2323 |
| SEL1L3 | SEL1L3 | SEL1L family member 3 | 23231 |
| TDRD7 | TDRD7 | tudor domain containing 7 | 23424 |
| TNFRSF13B | TNFRSF13B | TNF receptor superfamily member 13B | 23495 |
| ALOX5AP | ALOX5AP | arachidonate 5-lipoxygenase activating protein | 241 |
| FYN | FYN | FYN proto-oncogene, Src family tyrosine kinase | 2534 |
| ARHGAP30 | ARHGAP30 | Rho GTPase activating protein 30 | 257106 |
| QPCT | QPCT | glutaminyl-peptide cyclotransferase | 25797 |
| ABI3BP | ABI3BP | ABI family member 3 binding protein | 25890 |
| IFFO1 | IFFO1 | intermediate filament family orphan 1 | 25900 |
| PPP1R16B | PPP1R16B | protein phosphatase 1 regulatory subunit 16B | 26051 |
| PTPN22 | PTPN22 | protein tyrosine phosphatase, non-receptor type 22 | 26191 |
| GBP1 | GBP1 | guanylate binding protein 1 | 2633 |
| GBP2 | GBP2 | guanylate binding protein 2 | 2634 |
| AMPD1 | AMPD1 | adenosine monophosphate deaminase 1 | 270 |
| LAT | LAT | linker for activation of T-cells | 27040 |
| LAMP3 | LAMP3 | lysosomal associated membrane protein 3 | 27074 |
| CYTH4 | CYTH4 | cytohesin 4 | 27128 |
| ADAMDEC1 | ADAMDEC1 | ADAM like decysin 1 | 27299 |
| P2RY10 | P2RY10 | purinergic receptor P2Y10 | 27334 |
| XCR1 | XCR1 | X-C motif chemokine receptor 1 | 2829 |
| CXCR3 | CXCR3 | C-X-C motif chemokine receptor 3 | 2833 |
| GPR18 | GPR18 | G protein-coupled receptor 18 | 2841 |
| SLC9A9 | SLC9A9 | solute carrier family 9 member A9 | 285195 |
| TRBV29-1 | TRBV29-1 | T cell receptor beta variable 29-1 | 28558 |
| TRAV21 | TRAV21 | T cell receptor alpha variable 21 | 28662 |
| TRAC | TRAC | T-cell receptor alpha constant | 28755 |
| IGKV1D-8 | IGKV1D-8 | immunoglobulin kappa variable 1D-8 | 28904 |
| IGKV5-2 | IGKV5-2 | immunoglobulin kappa variable 5-2 | 28907 |
| IGKV2-40 | IGKV2-40 | immunoglobulin kappa variable 2-40 | 28916 |
| IGKV1-33 | IGKV1-33 | immunoglobulin kappa variable 1-33 | 28933 |
| VPREB3 | VPREB3 | pre-B lymphocyte 3 | 29802 |
| MDFIC | MDFIC | MyoD family inhibitor domain containing | 29969 |
| GZMH | GZMH | granzyme H | 2999 |
| GZMA | GZMA | granzyme A | 3001 |
| GZMB | GZMB | granzyme B | 3002 |
| GZMK | GZMK | granzyme K | 3003 |
| HCK | HCK | HCK proto-oncogene, Src family tyrosine kinase | 3055 |
| HCLS1 | HCLS1 | hematopoietic cell-specific Lyn substrate 1 | 3059 |
| NCKAP1L | NCKAP1L | NCK associated protein 1 like | 3071 |
| HLA-A | HLA-A | major histocompatibility complex, class I, A | 3105 |
| HLA-DMA | HLA-DMA | major histocompatibility complex, class II, DM alpha | 3108 |
| HLA-DMB | HLA-DMB | major histocompatibility complex, class II, DM beta | 3109 |
| HLA-DOB | HLA-DOB | major histocompatibility complex, class II, DO beta | 3112 |
| HLA-DPA1 | HLA-DPA1 | major histocompatibility complex, class II, DP alpha 1 | 3113 |
| HLA-DPB1 | HLA-DPB1 | major histocompatibility complex, class II, DP beta 1 | 3115 |
| HLA-DRA | HLA-DRA | major histocompatibility complex, class II, DR alpha | 3122 |
| AOAH | AOAH | acyloxyacyl hydrolase | 313 |
| HLA-E | HLA-E | major histocompatibility complex, class I, E | 3133 |
| HLA-F | HLA-F | major histocompatibility complex, class I, F | 3134 |
| HLA-G | HLA-G | major histocompatibility complex, class I, G | 3135 |
| ICAM2 | ICAM2 | intercellular adhesion molecule 2 | 3384 |
| IRF8 | IRF8 | interferon regulatory factor 8 | 3394 |
| TMEM173 | TMEM173 | transmembrane protein 173 | 340061 |
| APOC1 | APOC1 | apolipoprotein C1 | 341 |
| IFI16 | IFI16 | interferon gamma inducible protein 16 | 3428 |
| IFI27 | IFI27 | interferon alpha inducible protein 27 | 3429 |
| IFI35 | IFI35 | interferon induced protein 35 | 3430 |
| IFIT1 | IFIT1 | interferon induced protein with tetratricopeptide repeats 1 | 3434 |
| IFIT3 | IFIT3 | interferon induced protein with tetratricopeptide repeats 3 | 3437 |
| IFNG | IFNG | interferon gamma | 3458 |
| APOE | APOE | apolipoprotein E | 348 |
| IGKV3OR2-268 | IGKV3OR2-268 | immunoglobulin kappa variable 3/OR2-268 (non-functional) | 3523 |
| IL2RA | IL2RA | interleukin 2 receptor subunit alpha | 3559 |
| FASLG | FASLG | Fas ligand | 356 |
| IL2RB | IL2RB | interleukin 2 receptor subunit beta | 3560 |
| IL2RG | IL2RG | interleukin 2 receptor subunit gamma | 3561 |
| IL7R | IL7R | interleukin 7 receptor | 3575 |
| CXCR2P1 | CXCR2P1 | C-X-C motif chemokine receptor 2 pseudogene 1 | 3580 |
| IL10RA | IL10RA | interleukin 10 receptor subunit alpha | 3587 |
| IDO1 | IDO1 | indoleamine 2,3-dioxygenase 1 | 3620 |
| CXCL10 | CXCL10 | C-X-C motif chemokine ligand 10 | 3627 |
| IRF1 | IRF1 | interferon regulatory factor 1 | 3659 |
| IRF4 | IRF4 | interferon regulatory factor 4 | 3662 |
| IRF7 | IRF7 | interferon regulatory factor 7 | 3665 |
| ITGAL | ITGAL | integrin subunit alpha L | 3683 |
| ITGAX | ITGAX | integrin subunit alpha X | 3687 |
| ITGB2 | ITGB2 | integrin subunit beta 2 | 3689 |
| ITGB7 | ITGB7 | integrin subunit beta 7 | 3695 |
| ITK | ITK | IL2 inducible T-cell kinase | 3702 |
| TBC1D10C | TBC1D10C | TBC1 domain family member 10C | 374403 |
| KIR2DL3 | KIR2DL3 | killer cell immunoglobulin like receptor, two Ig domains and long cytoplasmic tail 3 | 3804 |
| KLRB1 | KLRB1 | killer cell lectin like receptor B1 | 3820 |
| THEMIS | THEMIS | thymocyte selection associated | 387357 |
| GVINP1 | GVINP1 | GTPase, very large interferon inducible pseudogene 1 | 387751 |
| RPL13AP20 | RPL13AP20 | ribosomal protein L13a pseudogene 20 | 387841 |
| MAFA | MAFA | MAF bZIP transcription factor A | 389692 |
| LAG3 | LAG3 | lymphocyte activating 3 | 3902 |
| LCK | LCK | LCK proto-oncogene, Src family tyrosine kinase | 3932 |
| LCP1 | LCP1 | lymphocyte cytosolic protein 1 | 3936 |
| LCP2 | LCP2 | lymphocyte cytosolic protein 2 | 3937 |
| LRMP | LRMP | lymphoid restricted membrane protein | 4033 |
| LTB | LTB | lymphotoxin beta | 4050 |
| LY9 | LY9 | lymphocyte antigen 9 | 4063 |
| LYN | LYN | LYN proto-oncogene, Src family tyrosine kinase | 4067 |
| SH2D1A | SH2D1A | SH2 domain containing 1A | 4068 |
| LYZ | LYZ | lysozyme | 4069 |
| MBP | MBP | myelin basic protein | 4155 |
| MEF2C | MEF2C | myocyte enhancer factor 2C | 4208 |
| MFNG | MFNG | MFNG O-fucosylpeptide 3-beta-N-acetylglucosaminyltransferase | 4242 |
| CIITA | CIITA | class II major histocompatibility complex transactivator | 4261 |
| CXCL9 | CXCL9 | C-X-C motif chemokine ligand 9 | 4283 |
| MPP1 | MPP1 | membrane palmitoylated protein 1 | 4354 |
| MYO1F | MYO1F | myosin IF | 4542 |
| MX1 | MX1 | MX dynamin like GTPase 1 | 4599 |
| MX2 | MX2 | MX dynamin like GTPase 2 | 4600 |
| GIMAP6 | GIMAP6 | GTPase, IMAP family member 6 | 474344 |
| NELL2 | NELL2 | neural EGFL like 2 | 4753 |
| NKG7 | NKG7 | natural killer cell granule protein 7 | 4818 |
| OAS1 | OAS1 | 2'-5'-oligoadenylate synthetase 1 | 4938 |
| OAS2 | OAS2 | 2'-5'-oligoadenylate synthetase 2 | 4939 |
| OAS3 | OAS3 | 2'-5'-oligoadenylate synthetase 3 | 4940 |
| TIFAB | TIFAB | TIFA inhibitor | 497189 |
| IL21R | IL21R | interleukin 21 receptor | 50615 |
| TRAT1 | TRAT1 | T-cell receptor associated transmembrane adaptor 1 | 50852 |
| PLEKHO1 | PLEKHO1 | pleckstrin homology domain containing O1 | 51177 |
| HERC5 | HERC5 | HECT and RLD domain containing E3 ubiquitin protein ligase 5 | 51191 |
| CLEC1A | CLEC1A | C-type lectin domain family 1 member A | 51267 |
| TLR7 | TLR7 | toll like receptor 7 | 51284 |
| SLC15A3 | SLC15A3 | solute carrier family 15 member 3 | 51296 |
| TLR8 | TLR8 | toll like receptor 8 | 51311 |
| PLAC8 | PLAC8 | placenta specific 8 | 51316 |
| TRPV2 | TRPV2 | transient receptor potential cation channel subfamily V member 2 | 51393 |
| ENPP2 | ENPP2 | ectonucleotide pyrophosphatase/phosphodiesterase 2 | 5168 |
| CECR1 | CECR1 | cat eye syndrome chromosome region, candidate 1 | 51816 |
| PIK3CD | PIK3CD | phosphatidylinositol-4,5-bisphosphate 3-kinase catalytic subunit delta | 5293 |
| PIK3CG | PIK3CG | phosphatidylinositol-4,5-bisphosphate 3-kinase catalytic subunit gamma | 5294 |
| PIP4K2A | PIP4K2A | phosphatidylinositol-5-phosphate 4-kinase type 2 alpha | 5305 |
| PLCB2 | PLCB2 | phospholipase C beta 2 | 5330 |
| PLCG2 | PLCG2 | phospholipase C gamma 2 | 5336 |
| PLEK | PLEK | pleckstrin | 5341 |
| PLSCR1 | PLSCR1 | phospholipid scramblase 1 | 5359 |
| PNOC | PNOC | prepronociceptin | 5368 |
| SASH3 | SASH3 | SAM and SH3 domain containing 3 | 54440 |
| CPVL | CPVL | carboxypeptidase, vitellogenic like | 54504 |
| APBB1IP | APBB1IP | amyloid beta precursor protein binding family B member 1 interacting protein | 54518 |
| PARP14 | PARP14 | poly(ADP-ribose) polymerase family member 14 | 54625 |
| SAMD9 | SAMD9 | sterile alpha motif domain containing 9 | 54809 |
| FAM46C | FAM46C | family with sequence similarity 46 member C | 54855 |
| LAX1 | LAX1 | lymphocyte transmembrane adaptor 1 | 54900 |
| HERC6 | HERC6 | HECT and RLD domain containing E3 ubiquitin protein ligase family member 6 | 55008 |
| PPP2R3C | PPP2R3C | protein phosphatase 2 regulatory subunit B''gamma | 55012 |
| MARCH1 | MARCH1 | membrane associated ring-CH-type finger 1 | 55016 |
| BANK1 | BANK1 | B-cell scaffold protein with ankyrin repeats 1 | 55024 |
| TAPBPL | TAPBPL | TAP binding protein like | 55080 |
| KIAA1551 | KIAA1551 | KIAA1551 | 55196 |
| TMEM140 | TMEM140 | transmembrane protein 140 | 55281 |
| GIMAP4 | GIMAP4 | GTPase, IMAP family member 4 | 55303 |
| TMEM176A | TMEM176A | transmembrane protein 176A | 55365 |
| SIRPG | SIRPG | signal regulatory protein gamma | 55423 |
| PRF1 | PRF1 | perforin 1 | 5551 |
| SRGN | SRGN | serglycin | 5552 |
| DDX60 | DDX60 | DExD/H-box helicase 60 | 55601 |
| DOCK10 | DOCK10 | dedicator of cytokinesis 10 | 55619 |
| FLVCR2 | FLVCR2 | feline leukemia virus subgroup C cellular receptor family member 2 | 55640 |
| ARHGAP15 | ARHGAP15 | Rho GTPase activating protein 15 | 55843 |
| SUCNR1 | SUCNR1 | succinate receptor 1 | 56670 |
| SLAMF8 | SLAMF8 | SLAM family member 8 | 56833 |
| PSMB8 | PSMB8 | proteasome subunit beta 8 | 5696 |
| PSMB9 | PSMB9 | proteasome subunit beta 9 | 5698 |
| PSMB10 | PSMB10 | proteasome subunit beta 10 | 5699 |
| CDC42SE2 | CDC42SE2 | CDC42 small effector 2 | 56990 |
| LPAR5 | LPAR5 | lysophosphatidic acid receptor 5 | 57121 |
| ZNFX1 | ZNFX1 | zinc finger NFX1-type containing 1 | 57169 |
| CAMK1G | CAMK1G | calcium/calmodulin dependent protein kinase IG | 57172 |
| PTGER4 | PTGER4 | prostaglandin E receptor 4 | 5734 |
| PTPN6 | PTPN6 | protein tyrosine phosphatase, non-receptor type 6 | 5777 |
| PTPN7 | PTPN7 | protein tyrosine phosphatase, non-receptor type 7 | 5778 |
| SLAMF7 | SLAMF7 | SLAM family member 7 | 57823 |
| PTPRE | PTPRE | protein tyrosine phosphatase, receptor type E | 5791 |
| RAC2 | RAC2 | ras-related C3 botulinum toxin substrate 2 (rho family, small GTP binding protein Rac2) | 5880 |
| RNASE6 | RNASE6 | ribonuclease A family member k6 | 6039 |
| TNFRSF17 | TNFRSF17 | TNF receptor superfamily member 17 | 608 |
| CCL2 | CCL2 | C-C motif chemokine ligand 2 | 6347 |
| CCL4 | CCL4 | C-C motif chemokine ligand 4 | 6351 |
| CCL5 | CCL5 | C-C motif chemokine ligand 5 | 6352 |
| GPSM3 | GPSM3 | G-protein signaling modulator 3 | 63940 |
| SELL | SELL | selectin L | 6402 |
| SAMSN1 | SAMSN1 | SAM domain, SH3 domain and nuclear localization signals 1 | 64092 |
| PARVG | PARVG | parvin gamma | 64098 |
| RTP4 | RTP4 | receptor transporter protein 4 | 64108 |
| DPEP2 | DPEP2 | dipeptidase 2 | 64174 |
| MS4A6A | MS4A6A | membrane spanning 4-domains A6A | 64231 |
| ARHGAP9 | ARHGAP9 | Rho GTPase activating protein 9 | 64333 |
| RGS18 | RGS18 | regulator of G-protein signaling 18 | 64407 |
| ARRDC5 | ARRDC5 | arrestin domain containing 5 | 645432 |
| SMAP2 | SMAP2 | small ArfGAP2 | 64744 |
| PARP12 | PARP12 | poly(ADP-ribose) polymerase family member 12 | 64761 |
| SLA | SLA | Src like adaptor | 6503 |
| SLAMF1 | SLAMF1 | signaling lymphocytic activation molecule family member 1 | 6504 |
| NCF1C | NCF1C | neutrophil cytosolic factor 1C pseudogene | 654817 |
| SPI1 | SPI1 | Spi-1 proto-oncogene | 6688 |
| STAT1 | STAT1 | signal transducer and activator of transcription 1 | 6772 |
| STAT4 | STAT4 | signal transducer and activator of transcription 4 | 6775 |
| ZFP36L2 | ZFP36L2 | ZFP36 ring finger protein like 2 | 678 |
| SYK | SYK | spleen associated tyrosine kinase | 6850 |
| TAP1 | TAP1 | transporter 1, ATP binding cassette subfamily B member | 6890 |
| TAP2 | TAP2 | transporter 2, ATP binding cassette subfamily B member | 6891 |
| TAPBP | TAPBP | TAP binding protein | 6892 |
| TBXAS1 | TBXAS1 | thromboxane A synthase 1 | 6916 |
| TCN2 | TCN2 | transcobalamin 2 | 6948 |
| BTK | BTK | Bruton tyrosine kinase | 695 |
| TLR4 | TLR4 | toll like receptor 4 | 7099 |
| SERPING1 | SERPING1 | serpin family G member 1 | 710 |
| C1QA | C1QA | complement C1q A chain | 712 |
| TNFAIP3 | TNFAIP3 | TNF alpha induced protein 3 | 7128 |
| C1QB | C1QB | complement C1q B chain | 713 |
| TNFRSF1B | TNFRSF1B | TNF receptor superfamily member 1B | 7133 |
| C2 | C2 | complement C2 | 717 |
| TRAF1 | TRAF1 | TNF receptor associated factor 1 | 7185 |
| C3AR1 | C3AR1 | complement C3a receptor 1 | 719 |
| CCR2 | CCR2 | C-C motif chemokine receptor 2 | 729230 |
| TNFRSF4 | TNFRSF4 | TNF receptor superfamily member 4 | 7293 |
| TYROBP | TYROBP | TYRO protein tyrosine kinase binding protein | 7305 |
| VAV1 | VAV1 | vav guanine nucleotide exchange factor 1 | 7409 |
| WARS | WARS | tryptophanyl-tRNA synthetase | 7453 |
| WAS | WAS | Wiskott-Aldrich syndrome | 7454 |
| WIPF1 | WIPF1 | WAS/WASL interacting protein family member 1 | 7456 |
| LAT2 | LAT2 | linker for activation of T-cells family member 2 | 7462 |
| ZAP70 | ZAP70 | zeta chain of T-cell receptor associated protein kinase 70 | 7535 |
| LAPTM5 | LAPTM5 | lysosomal protein transmembrane 5 | 7805 |
| ST8SIA4 | ST8SIA4 | ST8 alpha-N-acetyl-neuraminide alpha-2,8-sialyltransferase 4 | 7903 |
| LST1 | LST1 | leukocyte specific transcript 1 | 7940 |
| ZBED2 | ZBED2 | zinc finger BED-type containing 2 | 79413 |
| IGFLR1 | IGFLR1 | IGF like family receptor 1 | 79713 |
| DENND1C | DENND1C | DENN domain containing 1C | 79958 |
| PLEKHO2 | PLEKHO2 | pleckstrin homology domain containing O2 | 80301 |
| TRAF3IP3 | TRAF3IP3 | TRAF3 interacting protein 3 | 80342 |
| APOL6 | APOL6 | apolipoprotein L6 | 80830 |
| NPL | NPL | N-acetylneuraminate pyruvate lyase | 80896 |
| TCL1A | TCL1A | T-cell leukemia/lymphoma 1A | 8115 |
| PLVAP | PLVAP | plasmalemma vesicle associated protein | 83483 |
| FERMT3 | FERMT3 | fermitin family member 3 | 83706 |
| HAVCR2 | HAVCR2 | hepatitis A virus cellular receptor 2 | 84868 |
| TOX2 | TOX2 | TOX high mobility group box family member 2 | 84969 |
| IFITM1 | IFITM1 | interferon induced transmembrane protein 1 | 8519 |
| CST7 | CST7 | cystatin F | 8530 |
| SHANK3 | SHANK3 | SH3 and multiple ankyrin repeat domains 3 | 85358 |
| SKAP1 | SKAP1 | src kinase associated phosphoprotein 1 | 8631 |
| OASL | OASL | 2'-5'-oligoadenylate synthetase like | 8638 |
| S1PR4 | S1PR4 | sphingosine-1-phosphate receptor 4 | 8698 |
| TNFSF13 | TNFSF13 | tumor necrosis factor superfamily member 13 | 8741 |
| TNFRSF14 | TNFRSF14 | TNF receptor superfamily member 14 | 8764 |
| AP1S2 | AP1S2 | adaptor related protein complex 1 sigma 2 subunit | 8905 |
| CCND2 | CCND2 | cyclin D2 | 894 |
| SIGLEC10 | SIGLEC10 | sialic acid binding Ig like lectin 10 | 89790 |
| PSTPIP1 | PSTPIP1 | proline-serine-threonine phosphatase interacting protein 1 | 9051 |
| SLC7A7 | SLC7A7 | solute carrier family 7 member 7 | 9056 |
| N4BP2L1 | N4BP2L1 | NEDD4 binding protein 2 like 1 | 90634 |
| CD2 | CD2 | CD2 molecule | 914 |
| CD3D | CD3D | CD3d molecule | 915 |
| RSAD2 | RSAD2 | radical S-adenosyl methionine domain containing 2 | 91543 |
| CD3E | CD3E | CD3e molecule | 916 |
| CD247 | CD247 | CD247 molecule | 919 |
| CD4 | CD4 | CD4 molecule | 920 |
| RCSD1 | RCSD1 | RCSD domain containing 1 | 92241 |
| CD6 | CD6 | CD6 molecule | 923 |
| IL32 | IL32 | interleukin 32 | 9235 |
| CD7 | CD7 | CD7 molecule | 924 |
| UBE2L6 | UBE2L6 | ubiquitin conjugating enzyme E2 L6 | 9246 |
| CD8A | CD8A | CD8a molecule | 925 |
| CD14 | CD14 | CD14 molecule | 929 |
| CD83 | CD83 | CD83 molecule | 9308 |
| CD86 | CD86 | CD86 molecule | 942 |
| AIM2 | AIM2 | absent in melanoma 2 | 9447 |
| CD37 | CD37 | CD37 molecule | 951 |
| CD38 | CD38 | CD38 molecule | 952 |
| CD40LG | CD40LG | CD40 ligand | 959 |
| CD48 | CD48 | CD48 molecule | 962 |
| CD53 | CD53 | CD53 molecule | 963 |
| ISG15 | ISG15 | ISG15 ubiquitin-like modifier | 9636 |
| CD69 | CD69 | CD69 molecule | 969 |
| CD74 | CD74 | CD74 molecule | 972 |
| CD79B | CD79B | CD79b molecule | 974 |
| FAM65B | FAM65B | family with sequence similarity 65 member B | 9750 |
| SPOCK2 | SPOCK2 | SPARC/osteonectin, cwcv and kazal like domains proteoglycan 2 | 9806 |
| TESPA1 | TESPA1 | thymocyte expressed, positive selection associated 1 | 9840 |
| TRANK1 | TRANK1 | tetratricopeptide repeat and ankyrin repeat containing 1 | 9881 |

#### User IDs mapped to multiple Entrtez IDs or not mapped

userid

HMHA1

KIAA0125

KIAA0226L

#### GOSlim summary for the user list genes

Each Biological Process, Cellular Component and Molecular Function category is represented by a red, blue and green bar, repectively.  
The height of the bar represents the number of user list genes observed in the category.

#### Detailed information of the enriched categories

The statistics
 **- C: the number of reference genes in the category
- O: the number of genes in the user gene list and also in the category
- E: The expected number in the category
- R: ratio of enrichment
- PValue: p value from hyergeometric test
- FDR: FDR from BH** 
for the enriched categories and the genes in the user gene list and also in the category are listed in the table.

- GO:0001775 cell activation
- GO:0001816 cytokine production
- GO:0001817 regulation of cytokine production
- GO:0001819 positive regulation of cytokine production
- GO:0002250 adaptive immune response
- GO:0002252 immune effector process
- GO:0002253 activation of immune response
- GO:0002263 cell activation involved in immune response
- GO:0002274 myeloid leukocyte activation
- GO:0002366 leukocyte activation involved in immune response
- GO:0002429 immune response-activating cell surface receptor signaling pathway
- GO:0002443 leukocyte mediated immunity
- GO:0002449 lymphocyte mediated immunity
- GO:0002460 adaptive immune response based on somatic recombination of immune receptors built from immunoglobulin superfamily domains
- GO:0002520 immune system development
- GO:0002521 leukocyte differentiation
- GO:0002682 regulation of immune system process
- GO:0002683 negative regulation of immune system process
- GO:0002684 positive regulation of immune system process
- GO:0002694 regulation of leukocyte activation
- GO:0002696 positive regulation of leukocyte activation
- GO:0002697 regulation of immune effector process
- GO:0002757 immune response-activating signal transduction
- GO:0002764 immune response-regulating signaling pathway
- GO:0002768 immune response-regulating cell surface receptor signaling pathway
- GO:0006952 defense response
- GO:0006954 inflammatory response
- GO:0006955 immune response
- GO:0007155 cell adhesion
- GO:0007159 leukocyte cell-cell adhesion
- GO:0009605 response to external stimulus
- GO:0009607 response to biotic stimulus
- GO:0009615 response to virus
- GO:0016337 single organismal cell-cell adhesion
- GO:0019221 cytokine-mediated signaling pathway
- GO:0022407 regulation of cell-cell adhesion
- GO:0022409 positive regulation of cell-cell adhesion
- GO:0022610 biological adhesion
- GO:0030097 hemopoiesis
- GO:0030098 lymphocyte differentiation

  

Download Table

| **ID:GO:0001775     &nbspName:cell activation** | | | |
| --- | --- | --- | --- |
| C=871; O=105; E=15.86; R=6.62; PValue=0e+00; FDR=0e+00 | | | |
| userid | Gene Symbol | Gene Name | Entrez Gene |
| RASGRP1 | RASGRP1 | RAS guanyl releasing protein 1 | 10125 |
| EBI3 | EBI3 | Epstein-Barr virus induced 3 | 10148 |
| IKZF1 | IKZF1 | IKAROS family zinc finger 1 | 10320 |
| TNFSF13B | TNFSF13B | tumor necrosis factor superfamily member 13b | 10673 |
| BTN3A1 | BTN3A1 | butyrophilin subfamily 3 member A1 | 11119 |
| CORO1A | CORO1A | coronin 1A | 11151 |
| NLRP3 | NLRP3 | NLR family pyrin domain containing 3 | 114548 |
| BATF2 | BATF2 | basic leucine zipper ATF-like transcription factor 2 | 116071 |
| CCR7 | CCR7 | C-C motif chemokine receptor 7 | 1236 |
| DOCK2 | DOCK2 | dedicator of cytokinesis 2 | 1794 |
| GPR183 | GPR183 | G protein-coupled receptor 183 | 1880 |
| AIF1 | AIF1 | allograft inflammatory factor 1 | 199 |
| PLCL2 | PLCL2 | phospholipase C like 2 | 23228 |
| FLT3LG | FLT3LG | fms related tyrosine kinase 3 ligand | 2323 |
| TNFRSF13B | TNFRSF13B | TNF receptor superfamily member 13B | 23495 |
| FYN | FYN | FYN proto-oncogene, Src family tyrosine kinase | 2534 |
| PTPN22 | PTPN22 | protein tyrosine phosphatase, non-receptor type 22 | 26191 |
| LAT | LAT | linker for activation of T-cells | 27040 |
| GPR18 | GPR18 | G protein-coupled receptor 18 | 2841 |
| NCKAP1L | NCKAP1L | NCK associated protein 1 like | 3071 |
| HLA-DMB | HLA-DMB | major histocompatibility complex, class II, DM beta | 3109 |
| HLA-DPA1 | HLA-DPA1 | major histocompatibility complex, class II, DP alpha 1 | 3113 |
| HLA-DPB1 | HLA-DPB1 | major histocompatibility complex, class II, DP beta 1 | 3115 |
| HLA-DRA | HLA-DRA | major histocompatibility complex, class II, DR alpha | 3122 |
| HLA-E | HLA-E | major histocompatibility complex, class I, E | 3133 |
| HLA-G | HLA-G | major histocompatibility complex, class I, G | 3135 |
| IFNG | IFNG | interferon gamma | 3458 |
| APOE | APOE | apolipoprotein E | 348 |
| IL2RA | IL2RA | interleukin 2 receptor subunit alpha | 3559 |
| IL7R | IL7R | interleukin 7 receptor | 3575 |
| IDO1 | IDO1 | indoleamine 2,3-dioxygenase 1 | 3620 |
| CXCL10 | CXCL10 | C-X-C motif chemokine ligand 10 | 3627 |
| IRF1 | IRF1 | interferon regulatory factor 1 | 3659 |
| IRF4 | IRF4 | interferon regulatory factor 4 | 3662 |
| ITGAL | ITGAL | integrin subunit alpha L | 3683 |
| ITGB2 | ITGB2 | integrin subunit beta 2 | 3689 |
| ITK | ITK | IL2 inducible T-cell kinase | 3702 |
| TBC1D10C | TBC1D10C | TBC1 domain family member 10C | 374403 |
| THEMIS | THEMIS | thymocyte selection associated | 387357 |
| LAG3 | LAG3 | lymphocyte activating 3 | 3902 |
| LCK | LCK | LCK proto-oncogene, Src family tyrosine kinase | 3932 |
| LCP1 | LCP1 | lymphocyte cytosolic protein 1 | 3936 |
| LCP2 | LCP2 | lymphocyte cytosolic protein 2 | 3937 |
| LY9 | LY9 | lymphocyte antigen 9 | 4063 |
| LYN | LYN | LYN proto-oncogene, Src family tyrosine kinase | 4067 |
| MEF2C | MEF2C | myocyte enhancer factor 2C | 4208 |
| MFNG | MFNG | MFNG O-fucosylpeptide 3-beta-N-acetylglucosaminyltransferase | 4242 |
| IL21R | IL21R | interleukin 21 receptor | 50615 |
| TLR7 | TLR7 | toll like receptor 7 | 51284 |
| TLR8 | TLR8 | toll like receptor 8 | 51311 |
| PIK3CD | PIK3CD | phosphatidylinositol-4,5-bisphosphate 3-kinase catalytic subunit delta | 5293 |
| PIK3CG | PIK3CG | phosphatidylinositol-4,5-bisphosphate 3-kinase catalytic subunit gamma | 5294 |
| PLCG2 | PLCG2 | phospholipase C gamma 2 | 5336 |
| PLEK | PLEK | pleckstrin | 5341 |
| PLSCR1 | PLSCR1 | phospholipid scramblase 1 | 5359 |
| SASH3 | SASH3 | SAM and SH3 domain containing 3 | 54440 |
| APBB1IP | APBB1IP | amyloid beta precursor protein binding family B member 1 interacting protein | 54518 |
| LAX1 | LAX1 | lymphocyte transmembrane adaptor 1 | 54900 |
| PPP2R3C | PPP2R3C | protein phosphatase 2 regulatory subunit B''gamma | 55012 |
| BANK1 | BANK1 | B-cell scaffold protein with ankyrin repeats 1 | 55024 |
| SIRPG | SIRPG | signal regulatory protein gamma | 55423 |
| PRF1 | PRF1 | perforin 1 | 5551 |
| PTGER4 | PTGER4 | prostaglandin E receptor 4 | 5734 |
| PTPN6 | PTPN6 | protein tyrosine phosphatase, non-receptor type 6 | 5777 |
| SLAMF7 | SLAMF7 | SLAM family member 7 | 57823 |
| PTPRE | PTPRE | protein tyrosine phosphatase, receptor type E | 5791 |
| RAC2 | RAC2 | ras-related C3 botulinum toxin substrate 2 (rho family, small GTP binding protein Rac2) | 5880 |
| CCL2 | CCL2 | C-C motif chemokine ligand 2 | 6347 |
| CCL5 | CCL5 | C-C motif chemokine ligand 5 | 6352 |
| SAMSN1 | SAMSN1 | SAM domain, SH3 domain and nuclear localization signals 1 | 64092 |
| SLAMF1 | SLAMF1 | signaling lymphocytic activation molecule family member 1 | 6504 |
| SPI1 | SPI1 | Spi-1 proto-oncogene | 6688 |
| ZFP36L2 | ZFP36L2 | ZFP36 ring finger protein like 2 | 678 |
| SYK | SYK | spleen associated tyrosine kinase | 6850 |
| BTK | BTK | Bruton tyrosine kinase | 695 |
| TLR4 | TLR4 | toll like receptor 4 | 7099 |
| TNFAIP3 | TNFAIP3 | TNF alpha induced protein 3 | 7128 |
| CCR2 | CCR2 | C-C motif chemokine receptor 2 | 729230 |
| TNFRSF4 | TNFRSF4 | TNF receptor superfamily member 4 | 7293 |
| TYROBP | TYROBP | TYRO protein tyrosine kinase binding protein | 7305 |
| VAV1 | VAV1 | vav guanine nucleotide exchange factor 1 | 7409 |
| WAS | WAS | Wiskott-Aldrich syndrome | 7454 |
| LAT2 | LAT2 | linker for activation of T-cells family member 2 | 7462 |
| ZAP70 | ZAP70 | zeta chain of T-cell receptor associated protein kinase 70 | 7535 |
| LST1 | LST1 | leukocyte specific transcript 1 | 7940 |
| FERMT3 | FERMT3 | fermitin family member 3 | 83706 |
| HAVCR2 | HAVCR2 | hepatitis A virus cellular receptor 2 | 84868 |
| TNFSF13 | TNFSF13 | tumor necrosis factor superfamily member 13 | 8741 |
| TNFRSF14 | TNFRSF14 | TNF receptor superfamily member 14 | 8764 |
| CD2 | CD2 | CD2 molecule | 914 |
| CD3D | CD3D | CD3d molecule | 915 |
| RSAD2 | RSAD2 | radical S-adenosyl methionine domain containing 2 | 91543 |
| CD3E | CD3E | CD3e molecule | 916 |
| CD247 | CD247 | CD247 molecule | 919 |
| CD4 | CD4 | CD4 molecule | 920 |
| CD6 | CD6 | CD6 molecule | 923 |
| CD7 | CD7 | CD7 molecule | 924 |
| CD8A | CD8A | CD8a molecule | 925 |
| CD83 | CD83 | CD83 molecule | 9308 |
| CD86 | CD86 | CD86 molecule | 942 |
| CD38 | CD38 | CD38 molecule | 952 |
| CD40LG | CD40LG | CD40 ligand | 959 |
| CD74 | CD74 | CD74 molecule | 972 |
| CD79B | CD79B | CD79b molecule | 974 |
| TESPA1 | TESPA1 | thymocyte expressed, positive selection associated 1 | 9840 |

  
  

Download Table

| **ID:GO:0001816     &nbspName:cytokine production** | | | |
| --- | --- | --- | --- |
| C=616; O=65; E=11.22; R=5.8; PValue=0e+00; FDR=0e+00 | | | |
| userid | Gene Symbol | Gene Name | Entrez Gene |
| RASGRP1 | RASGRP1 | RAS guanyl releasing protein 1 | 10125 |
| EBI3 | EBI3 | Epstein-Barr virus induced 3 | 10148 |
| CD96 | CD96 | CD96 molecule | 10225 |
| BTN3A1 | BTN3A1 | butyrophilin subfamily 3 member A1 | 11119 |
| NLRP3 | NLRP3 | NLR family pyrin domain containing 3 | 114548 |
| CCR7 | CCR7 | C-C motif chemokine receptor 7 | 1236 |
| CSF1R | CSF1R | colony stimulating factor 1 receptor | 1436 |
| FCN1 | FCN1 | ficolin 1 | 2219 |
| SYT11 | SYT11 | synaptotagmin 11 | 23208 |
| PTPN22 | PTPN22 | protein tyrosine phosphatase, non-receptor type 22 | 26191 |
| GBP1 | GBP1 | guanylate binding protein 1 | 2633 |
| GPR18 | GPR18 | G protein-coupled receptor 18 | 2841 |
| NCKAP1L | NCKAP1L | NCK associated protein 1 like | 3071 |
| HLA-DPA1 | HLA-DPA1 | major histocompatibility complex, class II, DP alpha 1 | 3113 |
| HLA-DPB1 | HLA-DPB1 | major histocompatibility complex, class II, DP beta 1 | 3115 |
| HLA-E | HLA-E | major histocompatibility complex, class I, E | 3133 |
| HLA-G | HLA-G | major histocompatibility complex, class I, G | 3135 |
| IRF8 | IRF8 | interferon regulatory factor 8 | 3394 |
| TMEM173 | TMEM173 | transmembrane protein 173 | 340061 |
| IFI16 | IFI16 | interferon gamma inducible protein 16 | 3428 |
| IFNG | IFNG | interferon gamma | 3458 |
| IDO1 | IDO1 | indoleamine 2,3-dioxygenase 1 | 3620 |
| IRF1 | IRF1 | interferon regulatory factor 1 | 3659 |
| IRF4 | IRF4 | interferon regulatory factor 4 | 3662 |
| IRF7 | IRF7 | interferon regulatory factor 7 | 3665 |
| ITK | ITK | IL2 inducible T-cell kinase | 3702 |
| LAG3 | LAG3 | lymphocyte activating 3 | 3902 |
| LCP2 | LCP2 | lymphocyte cytosolic protein 2 | 3937 |
| LTB | LTB | lymphotoxin beta | 4050 |
| LY9 | LY9 | lymphocyte antigen 9 | 4063 |
| LYN | LYN | LYN proto-oncogene, Src family tyrosine kinase | 4067 |
| HERC5 | HERC5 | HECT and RLD domain containing E3 ubiquitin protein ligase 5 | 51191 |
| TLR7 | TLR7 | toll like receptor 7 | 51284 |
| TLR8 | TLR8 | toll like receptor 8 | 51311 |
| PIK3CD | PIK3CD | phosphatidylinositol-4,5-bisphosphate 3-kinase catalytic subunit delta | 5293 |
| PIK3CG | PIK3CG | phosphatidylinositol-4,5-bisphosphate 3-kinase catalytic subunit gamma | 5294 |
| PLCG2 | PLCG2 | phospholipase C gamma 2 | 5336 |
| SASH3 | SASH3 | SAM and SH3 domain containing 3 | 54440 |
| BANK1 | BANK1 | B-cell scaffold protein with ankyrin repeats 1 | 55024 |
| SRGN | SRGN | serglycin | 5552 |
| DDX60 | DDX60 | DExD/H-box helicase 60 | 55601 |
| PTGER4 | PTGER4 | prostaglandin E receptor 4 | 5734 |
| GPSM3 | GPSM3 | G-protein signaling modulator 3 | 63940 |
| SLAMF1 | SLAMF1 | signaling lymphocytic activation molecule family member 1 | 6504 |
| SYK | SYK | spleen associated tyrosine kinase | 6850 |
| BTK | BTK | Bruton tyrosine kinase | 695 |
| TLR4 | TLR4 | toll like receptor 4 | 7099 |
| TNFAIP3 | TNFAIP3 | TNF alpha induced protein 3 | 7128 |
| C3AR1 | C3AR1 | complement C3a receptor 1 | 719 |
| CCR2 | CCR2 | C-C motif chemokine receptor 2 | 729230 |
| HAVCR2 | HAVCR2 | hepatitis A virus cellular receptor 2 | 84868 |
| TNFRSF14 | TNFRSF14 | TNF receptor superfamily member 14 | 8764 |
| CD2 | CD2 | CD2 molecule | 914 |
| RSAD2 | RSAD2 | radical S-adenosyl methionine domain containing 2 | 91543 |
| CD3E | CD3E | CD3e molecule | 916 |
| CD4 | CD4 | CD4 molecule | 920 |
| CD6 | CD6 | CD6 molecule | 923 |
| UBE2L6 | UBE2L6 | ubiquitin conjugating enzyme E2 L6 | 9246 |
| CD14 | CD14 | CD14 molecule | 929 |
| CD83 | CD83 | CD83 molecule | 9308 |
| CD86 | CD86 | CD86 molecule | 942 |
| AIM2 | AIM2 | absent in melanoma 2 | 9447 |
| CD40LG | CD40LG | CD40 ligand | 959 |
| ISG15 | ISG15 | ISG15 ubiquitin-like modifier | 9636 |
| CD74 | CD74 | CD74 molecule | 972 |

  
  

Download Table

| **ID:GO:0001817     &nbspName:regulation of cytokine production** | | | |
| --- | --- | --- | --- |
| C=559; O=60; E=10.18; R=5.9; PValue=0e+00; FDR=0e+00 | | | |
| userid | Gene Symbol | Gene Name | Entrez Gene |
| RASGRP1 | RASGRP1 | RAS guanyl releasing protein 1 | 10125 |
| EBI3 | EBI3 | Epstein-Barr virus induced 3 | 10148 |
| CD96 | CD96 | CD96 molecule | 10225 |
| NLRP3 | NLRP3 | NLR family pyrin domain containing 3 | 114548 |
| CCR7 | CCR7 | C-C motif chemokine receptor 7 | 1236 |
| CSF1R | CSF1R | colony stimulating factor 1 receptor | 1436 |
| FCN1 | FCN1 | ficolin 1 | 2219 |
| SYT11 | SYT11 | synaptotagmin 11 | 23208 |
| PTPN22 | PTPN22 | protein tyrosine phosphatase, non-receptor type 22 | 26191 |
| GBP1 | GBP1 | guanylate binding protein 1 | 2633 |
| GPR18 | GPR18 | G protein-coupled receptor 18 | 2841 |
| NCKAP1L | NCKAP1L | NCK associated protein 1 like | 3071 |
| HLA-DPA1 | HLA-DPA1 | major histocompatibility complex, class II, DP alpha 1 | 3113 |
| HLA-DPB1 | HLA-DPB1 | major histocompatibility complex, class II, DP beta 1 | 3115 |
| HLA-E | HLA-E | major histocompatibility complex, class I, E | 3133 |
| HLA-G | HLA-G | major histocompatibility complex, class I, G | 3135 |
| IRF8 | IRF8 | interferon regulatory factor 8 | 3394 |
| TMEM173 | TMEM173 | transmembrane protein 173 | 340061 |
| IFI16 | IFI16 | interferon gamma inducible protein 16 | 3428 |
| IFNG | IFNG | interferon gamma | 3458 |
| IDO1 | IDO1 | indoleamine 2,3-dioxygenase 1 | 3620 |
| IRF1 | IRF1 | interferon regulatory factor 1 | 3659 |
| IRF4 | IRF4 | interferon regulatory factor 4 | 3662 |
| IRF7 | IRF7 | interferon regulatory factor 7 | 3665 |
| LAG3 | LAG3 | lymphocyte activating 3 | 3902 |
| LTB | LTB | lymphotoxin beta | 4050 |
| LY9 | LY9 | lymphocyte antigen 9 | 4063 |
| LYN | LYN | LYN proto-oncogene, Src family tyrosine kinase | 4067 |
| HERC5 | HERC5 | HECT and RLD domain containing E3 ubiquitin protein ligase 5 | 51191 |
| TLR7 | TLR7 | toll like receptor 7 | 51284 |
| TLR8 | TLR8 | toll like receptor 8 | 51311 |
| PLCG2 | PLCG2 | phospholipase C gamma 2 | 5336 |
| SASH3 | SASH3 | SAM and SH3 domain containing 3 | 54440 |
| BANK1 | BANK1 | B-cell scaffold protein with ankyrin repeats 1 | 55024 |
| SRGN | SRGN | serglycin | 5552 |
| DDX60 | DDX60 | DExD/H-box helicase 60 | 55601 |
| PTGER4 | PTGER4 | prostaglandin E receptor 4 | 5734 |
| GPSM3 | GPSM3 | G-protein signaling modulator 3 | 63940 |
| SLAMF1 | SLAMF1 | signaling lymphocytic activation molecule family member 1 | 6504 |
| SYK | SYK | spleen associated tyrosine kinase | 6850 |
| BTK | BTK | Bruton tyrosine kinase | 695 |
| TLR4 | TLR4 | toll like receptor 4 | 7099 |
| TNFAIP3 | TNFAIP3 | TNF alpha induced protein 3 | 7128 |
| C3AR1 | C3AR1 | complement C3a receptor 1 | 719 |
| CCR2 | CCR2 | C-C motif chemokine receptor 2 | 729230 |
| HAVCR2 | HAVCR2 | hepatitis A virus cellular receptor 2 | 84868 |
| TNFRSF14 | TNFRSF14 | TNF receptor superfamily member 14 | 8764 |
| CD2 | CD2 | CD2 molecule | 914 |
| RSAD2 | RSAD2 | radical S-adenosyl methionine domain containing 2 | 91543 |
| CD3E | CD3E | CD3e molecule | 916 |
| CD4 | CD4 | CD4 molecule | 920 |
| CD6 | CD6 | CD6 molecule | 923 |
| UBE2L6 | UBE2L6 | ubiquitin conjugating enzyme E2 L6 | 9246 |
| CD14 | CD14 | CD14 molecule | 929 |
| CD83 | CD83 | CD83 molecule | 9308 |
| CD86 | CD86 | CD86 molecule | 942 |
| AIM2 | AIM2 | absent in melanoma 2 | 9447 |
| CD40LG | CD40LG | CD40 ligand | 959 |
| ISG15 | ISG15 | ISG15 ubiquitin-like modifier | 9636 |
| CD74 | CD74 | CD74 molecule | 972 |

  
  

Download Table

| **ID:GO:0001819     &nbspName:positive regulation of cytokine production** | | | |
| --- | --- | --- | --- |
| C=374; O=46; E=6.81; R=6.76; PValue=0e+00; FDR=0e+00 | | | |
| userid | Gene Symbol | Gene Name | Entrez Gene |
| RASGRP1 | RASGRP1 | RAS guanyl releasing protein 1 | 10125 |
| EBI3 | EBI3 | Epstein-Barr virus induced 3 | 10148 |
| NLRP3 | NLRP3 | NLR family pyrin domain containing 3 | 114548 |
| CCR7 | CCR7 | C-C motif chemokine receptor 7 | 1236 |
| CSF1R | CSF1R | colony stimulating factor 1 receptor | 1436 |
| FCN1 | FCN1 | ficolin 1 | 2219 |
| PTPN22 | PTPN22 | protein tyrosine phosphatase, non-receptor type 22 | 26191 |
| HLA-DPA1 | HLA-DPA1 | major histocompatibility complex, class II, DP alpha 1 | 3113 |
| HLA-DPB1 | HLA-DPB1 | major histocompatibility complex, class II, DP beta 1 | 3115 |
| HLA-E | HLA-E | major histocompatibility complex, class I, E | 3133 |
| HLA-G | HLA-G | major histocompatibility complex, class I, G | 3135 |
| IRF8 | IRF8 | interferon regulatory factor 8 | 3394 |
| TMEM173 | TMEM173 | transmembrane protein 173 | 340061 |
| IFI16 | IFI16 | interferon gamma inducible protein 16 | 3428 |
| IFNG | IFNG | interferon gamma | 3458 |
| IDO1 | IDO1 | indoleamine 2,3-dioxygenase 1 | 3620 |
| IRF1 | IRF1 | interferon regulatory factor 1 | 3659 |
| IRF4 | IRF4 | interferon regulatory factor 4 | 3662 |
| IRF7 | IRF7 | interferon regulatory factor 7 | 3665 |
| LTB | LTB | lymphotoxin beta | 4050 |
| LY9 | LY9 | lymphocyte antigen 9 | 4063 |
| TLR7 | TLR7 | toll like receptor 7 | 51284 |
| TLR8 | TLR8 | toll like receptor 8 | 51311 |
| PLCG2 | PLCG2 | phospholipase C gamma 2 | 5336 |
| SASH3 | SASH3 | SAM and SH3 domain containing 3 | 54440 |
| DDX60 | DDX60 | DExD/H-box helicase 60 | 55601 |
| PTGER4 | PTGER4 | prostaglandin E receptor 4 | 5734 |
| GPSM3 | GPSM3 | G-protein signaling modulator 3 | 63940 |
| SLAMF1 | SLAMF1 | signaling lymphocytic activation molecule family member 1 | 6504 |
| SYK | SYK | spleen associated tyrosine kinase | 6850 |
| TLR4 | TLR4 | toll like receptor 4 | 7099 |
| C3AR1 | C3AR1 | complement C3a receptor 1 | 719 |
| CCR2 | CCR2 | C-C motif chemokine receptor 2 | 729230 |
| HAVCR2 | HAVCR2 | hepatitis A virus cellular receptor 2 | 84868 |
| TNFRSF14 | TNFRSF14 | TNF receptor superfamily member 14 | 8764 |
| CD2 | CD2 | CD2 molecule | 914 |
| RSAD2 | RSAD2 | radical S-adenosyl methionine domain containing 2 | 91543 |
| CD3E | CD3E | CD3e molecule | 916 |
| CD4 | CD4 | CD4 molecule | 920 |
| CD6 | CD6 | CD6 molecule | 923 |
| CD14 | CD14 | CD14 molecule | 929 |
| CD83 | CD83 | CD83 molecule | 9308 |
| CD86 | CD86 | CD86 molecule | 942 |
| AIM2 | AIM2 | absent in melanoma 2 | 9447 |
| CD40LG | CD40LG | CD40 ligand | 959 |
| CD74 | CD74 | CD74 molecule | 972 |

  
  

Download Table

| **ID:GO:0002250     &nbspName:adaptive immune response** | | | |
| --- | --- | --- | --- |
| C=336; O=65; E=6.12; R=10.63; PValue=0e+00; FDR=0e+00 | | | |
| userid | Gene Symbol | Gene Name | Entrez Gene |
| EBI3 | EBI3 | Epstein-Barr virus induced 3 | 10148 |
| BTN3A3 | BTN3A3 | butyrophilin subfamily 3 member A3 | 10384 |
| CLEC10A | CLEC10A | C-type lectin domain family 10 member A | 10462 |
| CXCL13 | CXCL13 | C-X-C motif chemokine ligand 13 | 10563 |
| TNFSF13B | TNFSF13B | tumor necrosis factor superfamily member 13b | 10673 |
| CTSC | CTSC | cathepsin C | 1075 |
| LILRB4 | LILRB4 | leukocyte immunoglobulin like receptor B4 | 11006 |
| BTN3A1 | BTN3A1 | butyrophilin subfamily 3 member A1 | 11119 |
| NLRP3 | NLRP3 | NLR family pyrin domain containing 3 | 114548 |
| CTSS | CTSS | cathepsin S | 1520 |
| GPR183 | GPR183 | G protein-coupled receptor 183 | 1880 |
| TNFRSF13B | TNFRSF13B | TNF receptor superfamily member 13B | 23495 |
| FYN | FYN | FYN proto-oncogene, Src family tyrosine kinase | 2534 |
| LAT | LAT | linker for activation of T-cells | 27040 |
| LAMP3 | LAMP3 | lysosomal associated membrane protein 3 | 27074 |
| HLA-E | HLA-E | major histocompatibility complex, class I, E | 3133 |
| IFNG | IFNG | interferon gamma | 3458 |
| IL7R | IL7R | interleukin 7 receptor | 3575 |
| IRF1 | IRF1 | interferon regulatory factor 1 | 3659 |
| IRF4 | IRF4 | interferon regulatory factor 4 | 3662 |
| IRF7 | IRF7 | interferon regulatory factor 7 | 3665 |
| ITK | ITK | IL2 inducible T-cell kinase | 3702 |
| THEMIS | THEMIS | thymocyte selection associated | 387357 |
| LY9 | LY9 | lymphocyte antigen 9 | 4063 |
| LYN | LYN | LYN proto-oncogene, Src family tyrosine kinase | 4067 |
| SH2D1A | SH2D1A | SH2 domain containing 1A | 4068 |
| MEF2C | MEF2C | myocyte enhancer factor 2C | 4208 |
| TRAT1 | TRAT1 | T-cell receptor associated transmembrane adaptor 1 | 50852 |
| TLR8 | TLR8 | toll like receptor 8 | 51311 |
| PIK3CD | PIK3CD | phosphatidylinositol-4,5-bisphosphate 3-kinase catalytic subunit delta | 5293 |
| PIK3CG | PIK3CG | phosphatidylinositol-4,5-bisphosphate 3-kinase catalytic subunit gamma | 5294 |
| SASH3 | SASH3 | SAM and SH3 domain containing 3 | 54440 |
| LAX1 | LAX1 | lymphocyte transmembrane adaptor 1 | 54900 |
| PTPN6 | PTPN6 | protein tyrosine phosphatase, non-receptor type 6 | 5777 |
| SLAMF7 | SLAMF7 | SLAM family member 7 | 57823 |
| TNFRSF17 | TNFRSF17 | TNF receptor superfamily member 17 | 608 |
| SAMSN1 | SAMSN1 | SAM domain, SH3 domain and nuclear localization signals 1 | 64092 |
| SLAMF1 | SLAMF1 | signaling lymphocytic activation molecule family member 1 | 6504 |
| SYK | SYK | spleen associated tyrosine kinase | 6850 |
| TAP1 | TAP1 | transporter 1, ATP binding cassette subfamily B member | 6890 |
| TAP2 | TAP2 | transporter 2, ATP binding cassette subfamily B member | 6891 |
| BTK | BTK | Bruton tyrosine kinase | 695 |
| TLR4 | TLR4 | toll like receptor 4 | 7099 |
| SERPING1 | SERPING1 | serpin family G member 1 | 710 |
| C1QA | C1QA | complement C1q A chain | 712 |
| TNFAIP3 | TNFAIP3 | TNF alpha induced protein 3 | 7128 |
| C1QB | C1QB | complement C1q B chain | 713 |
| C2 | C2 | complement C2 | 717 |
| CCR2 | CCR2 | C-C motif chemokine receptor 2 | 729230 |
| WAS | WAS | Wiskott-Aldrich syndrome | 7454 |
| LAT2 | LAT2 | linker for activation of T-cells family member 2 | 7462 |
| ZAP70 | ZAP70 | zeta chain of T-cell receptor associated protein kinase 70 | 7535 |
| HAVCR2 | HAVCR2 | hepatitis A virus cellular receptor 2 | 84868 |
| SKAP1 | SKAP1 | src kinase associated phosphoprotein 1 | 8631 |
| TNFSF13 | TNFSF13 | tumor necrosis factor superfamily member 13 | 8741 |
| RSAD2 | RSAD2 | radical S-adenosyl methionine domain containing 2 | 91543 |
| CD4 | CD4 | CD4 molecule | 920 |
| CD6 | CD6 | CD6 molecule | 923 |
| CD7 | CD7 | CD7 molecule | 924 |
| CD8A | CD8A | CD8a molecule | 925 |
| CD86 | CD86 | CD86 molecule | 942 |
| CD40LG | CD40LG | CD40 ligand | 959 |
| CD48 | CD48 | CD48 molecule | 962 |
| CD74 | CD74 | CD74 molecule | 972 |
| CD79B | CD79B | CD79b molecule | 974 |

  
  

Download Table

| **ID:GO:0002252     &nbspName:immune effector process** | | | |
| --- | --- | --- | --- |
| C=659; O=97; E=12; R=8.08; PValue=0e+00; FDR=0e+00 | | | |
| userid | Gene Symbol | Gene Name | Entrez Gene |
| RASGRP1 | RASGRP1 | RAS guanyl releasing protein 1 | 10125 |
| CD96 | CD96 | CD96 molecule | 10225 |
| TRIM22 | TRIM22 | tripartite motif containing 22 | 10346 |
| BTN3A3 | BTN3A3 | butyrophilin subfamily 3 member A3 | 10384 |
| IFITM3 | IFITM3 | interferon induced transmembrane protein 3 | 10410 |
| IFITM2 | IFITM2 | interferon induced transmembrane protein 2 | 10581 |
| CTSC | CTSC | cathepsin C | 1075 |
| IFI44L | IFI44L | interferon induced protein 44 like | 10964 |
| CORO1A | CORO1A | coronin 1A | 11151 |
| NLRP3 | NLRP3 | NLR family pyrin domain containing 3 | 114548 |
| TAGAP | TAGAP | T-cell activation RhoGTPase activating protein | 117289 |
| DOCK2 | DOCK2 | dedicator of cytokinesis 2 | 1794 |
| GPR183 | GPR183 | G protein-coupled receptor 183 | 1880 |
| FCN1 | FCN1 | ficolin 1 | 2219 |
| PLCL2 | PLCL2 | phospholipase C like 2 | 23228 |
| FYN | FYN | FYN proto-oncogene, Src family tyrosine kinase | 2534 |
| GBP1 | GBP1 | guanylate binding protein 1 | 2633 |
| LAT | LAT | linker for activation of T-cells | 27040 |
| GZMB | GZMB | granzyme B | 3002 |
| HCK | HCK | HCK proto-oncogene, Src family tyrosine kinase | 3055 |
| NCKAP1L | NCKAP1L | NCK associated protein 1 like | 3071 |
| HLA-DMB | HLA-DMB | major histocompatibility complex, class II, DM beta | 3109 |
| HLA-E | HLA-E | major histocompatibility complex, class I, E | 3133 |
| TMEM173 | TMEM173 | transmembrane protein 173 | 340061 |
| IFI16 | IFI16 | interferon gamma inducible protein 16 | 3428 |
| IFIT1 | IFIT1 | interferon induced protein with tetratricopeptide repeats 1 | 3434 |
| IFIT3 | IFIT3 | interferon induced protein with tetratricopeptide repeats 3 | 3437 |
| IFNG | IFNG | interferon gamma | 3458 |
| IL2RA | IL2RA | interleukin 2 receptor subunit alpha | 3559 |
| IL7R | IL7R | interleukin 7 receptor | 3575 |
| CXCL10 | CXCL10 | C-X-C motif chemokine ligand 10 | 3627 |
| IRF1 | IRF1 | interferon regulatory factor 1 | 3659 |
| IRF4 | IRF4 | interferon regulatory factor 4 | 3662 |
| IRF7 | IRF7 | interferon regulatory factor 7 | 3665 |
| ITGAL | ITGAL | integrin subunit alpha L | 3683 |
| LAG3 | LAG3 | lymphocyte activating 3 | 3902 |
| LCK | LCK | LCK proto-oncogene, Src family tyrosine kinase | 3932 |
| LCP1 | LCP1 | lymphocyte cytosolic protein 1 | 3936 |
| LY9 | LY9 | lymphocyte antigen 9 | 4063 |
| LYN | LYN | LYN proto-oncogene, Src family tyrosine kinase | 4067 |
| SH2D1A | SH2D1A | SH2 domain containing 1A | 4068 |
| MFNG | MFNG | MFNG O-fucosylpeptide 3-beta-N-acetylglucosaminyltransferase | 4242 |
| CXCL9 | CXCL9 | C-X-C motif chemokine ligand 9 | 4283 |
| MX1 | MX1 | MX dynamin like GTPase 1 | 4599 |
| MX2 | MX2 | MX dynamin like GTPase 2 | 4600 |
| OAS1 | OAS1 | 2'-5'-oligoadenylate synthetase 1 | 4938 |
| OAS2 | OAS2 | 2'-5'-oligoadenylate synthetase 2 | 4939 |
| OAS3 | OAS3 | 2'-5'-oligoadenylate synthetase 3 | 4940 |
| HERC5 | HERC5 | HECT and RLD domain containing E3 ubiquitin protein ligase 5 | 51191 |
| TLR7 | TLR7 | toll like receptor 7 | 51284 |
| TLR8 | TLR8 | toll like receptor 8 | 51311 |
| PIK3CD | PIK3CD | phosphatidylinositol-4,5-bisphosphate 3-kinase catalytic subunit delta | 5293 |
| PIK3CG | PIK3CG | phosphatidylinositol-4,5-bisphosphate 3-kinase catalytic subunit gamma | 5294 |
| PLCG2 | PLCG2 | phospholipase C gamma 2 | 5336 |
| PLSCR1 | PLSCR1 | phospholipid scramblase 1 | 5359 |
| SASH3 | SASH3 | SAM and SH3 domain containing 3 | 54440 |
| APBB1IP | APBB1IP | amyloid beta precursor protein binding family B member 1 interacting protein | 54518 |
| PRF1 | PRF1 | perforin 1 | 5551 |
| DDX60 | DDX60 | DExD/H-box helicase 60 | 55601 |
| PTGER4 | PTGER4 | prostaglandin E receptor 4 | 5734 |
| PTPN6 | PTPN6 | protein tyrosine phosphatase, non-receptor type 6 | 5777 |
| SLAMF7 | SLAMF7 | SLAM family member 7 | 57823 |
| RAC2 | RAC2 | ras-related C3 botulinum toxin substrate 2 (rho family, small GTP binding protein Rac2) | 5880 |
| SLAMF1 | SLAMF1 | signaling lymphocytic activation molecule family member 1 | 6504 |
| STAT1 | STAT1 | signal transducer and activator of transcription 1 | 6772 |
| SYK | SYK | spleen associated tyrosine kinase | 6850 |
| BTK | BTK | Bruton tyrosine kinase | 695 |
| TLR4 | TLR4 | toll like receptor 4 | 7099 |
| SERPING1 | SERPING1 | serpin family G member 1 | 710 |
| C1QA | C1QA | complement C1q A chain | 712 |
| TNFAIP3 | TNFAIP3 | TNF alpha induced protein 3 | 7128 |
| C1QB | C1QB | complement C1q B chain | 713 |
| C2 | C2 | complement C2 | 717 |
| C3AR1 | C3AR1 | complement C3a receptor 1 | 719 |
| CCR2 | CCR2 | C-C motif chemokine receptor 2 | 729230 |
| TNFRSF4 | TNFRSF4 | TNF receptor superfamily member 4 | 7293 |
| TYROBP | TYROBP | TYRO protein tyrosine kinase binding protein | 7305 |
| VAV1 | VAV1 | vav guanine nucleotide exchange factor 1 | 7409 |
| WAS | WAS | Wiskott-Aldrich syndrome | 7454 |
| WIPF1 | WIPF1 | WAS/WASL interacting protein family member 1 | 7456 |
| LAT2 | LAT2 | linker for activation of T-cells family member 2 | 7462 |
| ZAP70 | ZAP70 | zeta chain of T-cell receptor associated protein kinase 70 | 7535 |
| HAVCR2 | HAVCR2 | hepatitis A virus cellular receptor 2 | 84868 |
| IFITM1 | IFITM1 | interferon induced transmembrane protein 1 | 8519 |
| OASL | OASL | 2'-5'-oligoadenylate synthetase like | 8638 |
| TNFSF13 | TNFSF13 | tumor necrosis factor superfamily member 13 | 8741 |
| TNFRSF14 | TNFRSF14 | TNF receptor superfamily member 14 | 8764 |
| AP1S2 | AP1S2 | adaptor related protein complex 1 sigma 2 subunit | 8905 |
| RSAD2 | RSAD2 | radical S-adenosyl methionine domain containing 2 | 91543 |
| CD247 | CD247 | CD247 molecule | 919 |
| CD4 | CD4 | CD4 molecule | 920 |
| CD8A | CD8A | CD8a molecule | 925 |
| CD86 | CD86 | CD86 molecule | 942 |
| AIM2 | AIM2 | absent in melanoma 2 | 9447 |
| CD40LG | CD40LG | CD40 ligand | 959 |
| ISG15 | ISG15 | ISG15 ubiquitin-like modifier | 9636 |
| CD74 | CD74 | CD74 molecule | 972 |

  
  

Download Table

| **ID:GO:0002253     &nbspName:activation of immune response** | | | |
| --- | --- | --- | --- |
| C=482; O=67; E=8.78; R=7.63; PValue=0e+00; FDR=0e+00 | | | |
| userid | Gene Symbol | Gene Name | Entrez Gene |
| BTN3A1 | BTN3A1 | butyrophilin subfamily 3 member A1 | 11119 |
| CCR7 | CCR7 | C-C motif chemokine receptor 7 | 1236 |
| CTSS | CTSS | cathepsin S | 1520 |
| FCN1 | FCN1 | ficolin 1 | 2219 |
| PLCL2 | PLCL2 | phospholipase C like 2 | 23228 |
| FYN | FYN | FYN proto-oncogene, Src family tyrosine kinase | 2534 |
| PTPN22 | PTPN22 | protein tyrosine phosphatase, non-receptor type 22 | 26191 |
| GBP1 | GBP1 | guanylate binding protein 1 | 2633 |
| LAT | LAT | linker for activation of T-cells | 27040 |
| HCK | HCK | HCK proto-oncogene, Src family tyrosine kinase | 3055 |
| NCKAP1L | NCKAP1L | NCK associated protein 1 like | 3071 |
| HLA-DPA1 | HLA-DPA1 | major histocompatibility complex, class II, DP alpha 1 | 3113 |
| HLA-DPB1 | HLA-DPB1 | major histocompatibility complex, class II, DP beta 1 | 3115 |
| HLA-DRA | HLA-DRA | major histocompatibility complex, class II, DR alpha | 3122 |
| ICAM2 | ICAM2 | intercellular adhesion molecule 2 | 3384 |
| TMEM173 | TMEM173 | transmembrane protein 173 | 340061 |
| IFI16 | IFI16 | interferon gamma inducible protein 16 | 3428 |
| IFNG | IFNG | interferon gamma | 3458 |
| IRF1 | IRF1 | interferon regulatory factor 1 | 3659 |
| IRF4 | IRF4 | interferon regulatory factor 4 | 3662 |
| IRF7 | IRF7 | interferon regulatory factor 7 | 3665 |
| ITGB2 | ITGB2 | integrin subunit beta 2 | 3689 |
| ITK | ITK | IL2 inducible T-cell kinase | 3702 |
| THEMIS | THEMIS | thymocyte selection associated | 387357 |
| LCK | LCK | LCK proto-oncogene, Src family tyrosine kinase | 3932 |
| LCP2 | LCP2 | lymphocyte cytosolic protein 2 | 3937 |
| LYN | LYN | LYN proto-oncogene, Src family tyrosine kinase | 4067 |
| MEF2C | MEF2C | myocyte enhancer factor 2C | 4208 |
| TRAT1 | TRAT1 | T-cell receptor associated transmembrane adaptor 1 | 50852 |
| TLR7 | TLR7 | toll like receptor 7 | 51284 |
| TLR8 | TLR8 | toll like receptor 8 | 51311 |
| PIK3CD | PIK3CD | phosphatidylinositol-4,5-bisphosphate 3-kinase catalytic subunit delta | 5293 |
| PLCG2 | PLCG2 | phospholipase C gamma 2 | 5336 |
| PLSCR1 | PLSCR1 | phospholipid scramblase 1 | 5359 |
| LAX1 | LAX1 | lymphocyte transmembrane adaptor 1 | 54900 |
| DDX60 | DDX60 | DExD/H-box helicase 60 | 55601 |
| PSMB8 | PSMB8 | proteasome subunit beta 8 | 5696 |
| PSMB9 | PSMB9 | proteasome subunit beta 9 | 5698 |
| PSMB10 | PSMB10 | proteasome subunit beta 10 | 5699 |
| PTPN6 | PTPN6 | protein tyrosine phosphatase, non-receptor type 6 | 5777 |
| SYK | SYK | spleen associated tyrosine kinase | 6850 |
| BTK | BTK | Bruton tyrosine kinase | 695 |
| TLR4 | TLR4 | toll like receptor 4 | 7099 |
| SERPING1 | SERPING1 | serpin family G member 1 | 710 |
| C1QA | C1QA | complement C1q A chain | 712 |
| TNFAIP3 | TNFAIP3 | TNF alpha induced protein 3 | 7128 |
| C1QB | C1QB | complement C1q B chain | 713 |
| C2 | C2 | complement C2 | 717 |
| C3AR1 | C3AR1 | complement C3a receptor 1 | 719 |
| VAV1 | VAV1 | vav guanine nucleotide exchange factor 1 | 7409 |
| WAS | WAS | Wiskott-Aldrich syndrome | 7454 |
| WIPF1 | WIPF1 | WAS/WASL interacting protein family member 1 | 7456 |
| LAT2 | LAT2 | linker for activation of T-cells family member 2 | 7462 |
| ZAP70 | ZAP70 | zeta chain of T-cell receptor associated protein kinase 70 | 7535 |
| HAVCR2 | HAVCR2 | hepatitis A virus cellular receptor 2 | 84868 |
| SKAP1 | SKAP1 | src kinase associated phosphoprotein 1 | 8631 |
| CD3D | CD3D | CD3d molecule | 915 |
| RSAD2 | RSAD2 | radical S-adenosyl methionine domain containing 2 | 91543 |
| CD3E | CD3E | CD3e molecule | 916 |
| CD247 | CD247 | CD247 molecule | 919 |
| CD4 | CD4 | CD4 molecule | 920 |
| CD14 | CD14 | CD14 molecule | 929 |
| CD86 | CD86 | CD86 molecule | 942 |
| AIM2 | AIM2 | absent in melanoma 2 | 9447 |
| CD38 | CD38 | CD38 molecule | 952 |
| CD79B | CD79B | CD79b molecule | 974 |
| TESPA1 | TESPA1 | thymocyte expressed, positive selection associated 1 | 9840 |

  
  

Download Table

| **ID:GO:0002263     &nbspName:cell activation involved in immune response** | | | |
| --- | --- | --- | --- |
| C=217; O=33; E=3.95; R=8.35; PValue=0e+00; FDR=0e+00 | | | |
| userid | Gene Symbol | Gene Name | Entrez Gene |
| RASGRP1 | RASGRP1 | RAS guanyl releasing protein 1 | 10125 |
| CORO1A | CORO1A | coronin 1A | 11151 |
| NLRP3 | NLRP3 | NLR family pyrin domain containing 3 | 114548 |
| DOCK2 | DOCK2 | dedicator of cytokinesis 2 | 1794 |
| GPR183 | GPR183 | G protein-coupled receptor 183 | 1880 |
| PLCL2 | PLCL2 | phospholipase C like 2 | 23228 |
| LAT | LAT | linker for activation of T-cells | 27040 |
| HLA-DMB | HLA-DMB | major histocompatibility complex, class II, DM beta | 3109 |
| IFNG | IFNG | interferon gamma | 3458 |
| IRF4 | IRF4 | interferon regulatory factor 4 | 3662 |
| ITGAL | ITGAL | integrin subunit alpha L | 3683 |
| LCP1 | LCP1 | lymphocyte cytosolic protein 1 | 3936 |
| LY9 | LY9 | lymphocyte antigen 9 | 4063 |
| LYN | LYN | LYN proto-oncogene, Src family tyrosine kinase | 4067 |
| MFNG | MFNG | MFNG O-fucosylpeptide 3-beta-N-acetylglucosaminyltransferase | 4242 |
| PIK3CD | PIK3CD | phosphatidylinositol-4,5-bisphosphate 3-kinase catalytic subunit delta | 5293 |
| PIK3CG | PIK3CG | phosphatidylinositol-4,5-bisphosphate 3-kinase catalytic subunit gamma | 5294 |
| PLCG2 | PLCG2 | phospholipase C gamma 2 | 5336 |
| APBB1IP | APBB1IP | amyloid beta precursor protein binding family B member 1 interacting protein | 54518 |
| PTGER4 | PTGER4 | prostaglandin E receptor 4 | 5734 |
| RAC2 | RAC2 | ras-related C3 botulinum toxin substrate 2 (rho family, small GTP binding protein Rac2) | 5880 |
| SLAMF1 | SLAMF1 | signaling lymphocytic activation molecule family member 1 | 6504 |
| SYK | SYK | spleen associated tyrosine kinase | 6850 |
| BTK | BTK | Bruton tyrosine kinase | 695 |
| TLR4 | TLR4 | toll like receptor 4 | 7099 |
| CCR2 | CCR2 | C-C motif chemokine receptor 2 | 729230 |
| TYROBP | TYROBP | TYRO protein tyrosine kinase binding protein | 7305 |
| LAT2 | LAT2 | linker for activation of T-cells family member 2 | 7462 |
| ZAP70 | ZAP70 | zeta chain of T-cell receptor associated protein kinase 70 | 7535 |
| HAVCR2 | HAVCR2 | hepatitis A virus cellular receptor 2 | 84868 |
| TNFSF13 | TNFSF13 | tumor necrosis factor superfamily member 13 | 8741 |
| CD86 | CD86 | CD86 molecule | 942 |
| CD40LG | CD40LG | CD40 ligand | 959 |

  
  

Download Table

| **ID:GO:0002274     &nbspName:myeloid leukocyte activation** | | | |
| --- | --- | --- | --- |
| C=158; O=30; E=2.88; R=10.43; PValue=0e+00; FDR=0e+00 | | | |
| userid | Gene Symbol | Gene Name | Entrez Gene |
| RASGRP1 | RASGRP1 | RAS guanyl releasing protein 1 | 10125 |
| BATF2 | BATF2 | basic leucine zipper ATF-like transcription factor 2 | 116071 |
| DOCK2 | DOCK2 | dedicator of cytokinesis 2 | 1794 |
| AIF1 | AIF1 | allograft inflammatory factor 1 | 199 |
| FLT3LG | FLT3LG | fms related tyrosine kinase 3 ligand | 2323 |
| LAT | LAT | linker for activation of T-cells | 27040 |
| IRF4 | IRF4 | interferon regulatory factor 4 | 3662 |
| LCP2 | LCP2 | lymphocyte cytosolic protein 2 | 3937 |
| LYN | LYN | LYN proto-oncogene, Src family tyrosine kinase | 4067 |
| TLR7 | TLR7 | toll like receptor 7 | 51284 |
| TLR8 | TLR8 | toll like receptor 8 | 51311 |
| PIK3CD | PIK3CD | phosphatidylinositol-4,5-bisphosphate 3-kinase catalytic subunit delta | 5293 |
| PIK3CG | PIK3CG | phosphatidylinositol-4,5-bisphosphate 3-kinase catalytic subunit gamma | 5294 |
| PLSCR1 | PLSCR1 | phospholipid scramblase 1 | 5359 |
| PTPRE | PTPRE | protein tyrosine phosphatase, receptor type E | 5791 |
| RAC2 | RAC2 | ras-related C3 botulinum toxin substrate 2 (rho family, small GTP binding protein Rac2) | 5880 |
| CCL5 | CCL5 | C-C motif chemokine ligand 5 | 6352 |
| SLAMF1 | SLAMF1 | signaling lymphocytic activation molecule family member 1 | 6504 |
| SPI1 | SPI1 | Spi-1 proto-oncogene | 6688 |
| SYK | SYK | spleen associated tyrosine kinase | 6850 |
| BTK | BTK | Bruton tyrosine kinase | 695 |
| TLR4 | TLR4 | toll like receptor 4 | 7099 |
| CCR2 | CCR2 | C-C motif chemokine receptor 2 | 729230 |
| TYROBP | TYROBP | TYRO protein tyrosine kinase binding protein | 7305 |
| LAT2 | LAT2 | linker for activation of T-cells family member 2 | 7462 |
| ZAP70 | ZAP70 | zeta chain of T-cell receptor associated protein kinase 70 | 7535 |
| HAVCR2 | HAVCR2 | hepatitis A virus cellular receptor 2 | 84868 |
| CD2 | CD2 | CD2 molecule | 914 |
| CD86 | CD86 | CD86 molecule | 942 |
| CD74 | CD74 | CD74 molecule | 972 |

  
  

Download Table

| **ID:GO:0002366     &nbspName:leukocyte activation involved in immune response** | | | |
| --- | --- | --- | --- |
| C=215; O=33; E=3.91; R=8.43; PValue=0e+00; FDR=0e+00 | | | |
| userid | Gene Symbol | Gene Name | Entrez Gene |
| RASGRP1 | RASGRP1 | RAS guanyl releasing protein 1 | 10125 |
| CORO1A | CORO1A | coronin 1A | 11151 |
| NLRP3 | NLRP3 | NLR family pyrin domain containing 3 | 114548 |
| DOCK2 | DOCK2 | dedicator of cytokinesis 2 | 1794 |
| GPR183 | GPR183 | G protein-coupled receptor 183 | 1880 |
| PLCL2 | PLCL2 | phospholipase C like 2 | 23228 |
| LAT | LAT | linker for activation of T-cells | 27040 |
| HLA-DMB | HLA-DMB | major histocompatibility complex, class II, DM beta | 3109 |
| IFNG | IFNG | interferon gamma | 3458 |
| IRF4 | IRF4 | interferon regulatory factor 4 | 3662 |
| ITGAL | ITGAL | integrin subunit alpha L | 3683 |
| LCP1 | LCP1 | lymphocyte cytosolic protein 1 | 3936 |
| LY9 | LY9 | lymphocyte antigen 9 | 4063 |
| LYN | LYN | LYN proto-oncogene, Src family tyrosine kinase | 4067 |
| MFNG | MFNG | MFNG O-fucosylpeptide 3-beta-N-acetylglucosaminyltransferase | 4242 |
| PIK3CD | PIK3CD | phosphatidylinositol-4,5-bisphosphate 3-kinase catalytic subunit delta | 5293 |
| PIK3CG | PIK3CG | phosphatidylinositol-4,5-bisphosphate 3-kinase catalytic subunit gamma | 5294 |
| PLCG2 | PLCG2 | phospholipase C gamma 2 | 5336 |
| APBB1IP | APBB1IP | amyloid beta precursor protein binding family B member 1 interacting protein | 54518 |
| PTGER4 | PTGER4 | prostaglandin E receptor 4 | 5734 |
| RAC2 | RAC2 | ras-related C3 botulinum toxin substrate 2 (rho family, small GTP binding protein Rac2) | 5880 |
| SLAMF1 | SLAMF1 | signaling lymphocytic activation molecule family member 1 | 6504 |
| SYK | SYK | spleen associated tyrosine kinase | 6850 |
| BTK | BTK | Bruton tyrosine kinase | 695 |
| TLR4 | TLR4 | toll like receptor 4 | 7099 |
| CCR2 | CCR2 | C-C motif chemokine receptor 2 | 729230 |
| TYROBP | TYROBP | TYRO protein tyrosine kinase binding protein | 7305 |
| LAT2 | LAT2 | linker for activation of T-cells family member 2 | 7462 |
| ZAP70 | ZAP70 | zeta chain of T-cell receptor associated protein kinase 70 | 7535 |
| HAVCR2 | HAVCR2 | hepatitis A virus cellular receptor 2 | 84868 |
| TNFSF13 | TNFSF13 | tumor necrosis factor superfamily member 13 | 8741 |
| CD86 | CD86 | CD86 molecule | 942 |
| CD40LG | CD40LG | CD40 ligand | 959 |

  
  

Download Table

| **ID:GO:0002429     &nbspName:immune response-activating cell surface receptor signaling pathway** | | | |
| --- | --- | --- | --- |
| C=299; O=46; E=5.44; R=8.45; PValue=0e+00; FDR=0e+00 | | | |
| userid | Gene Symbol | Gene Name | Entrez Gene |
| BTN3A1 | BTN3A1 | butyrophilin subfamily 3 member A1 | 11119 |
| CCR7 | CCR7 | C-C motif chemokine receptor 7 | 1236 |
| FCN1 | FCN1 | ficolin 1 | 2219 |
| PLCL2 | PLCL2 | phospholipase C like 2 | 23228 |
| FYN | FYN | FYN proto-oncogene, Src family tyrosine kinase | 2534 |
| PTPN22 | PTPN22 | protein tyrosine phosphatase, non-receptor type 22 | 26191 |
| GBP1 | GBP1 | guanylate binding protein 1 | 2633 |
| LAT | LAT | linker for activation of T-cells | 27040 |
| HCK | HCK | HCK proto-oncogene, Src family tyrosine kinase | 3055 |
| NCKAP1L | NCKAP1L | NCK associated protein 1 like | 3071 |
| HLA-DPA1 | HLA-DPA1 | major histocompatibility complex, class II, DP alpha 1 | 3113 |
| HLA-DPB1 | HLA-DPB1 | major histocompatibility complex, class II, DP beta 1 | 3115 |
| HLA-DRA | HLA-DRA | major histocompatibility complex, class II, DR alpha | 3122 |
| ICAM2 | ICAM2 | intercellular adhesion molecule 2 | 3384 |
| IFNG | IFNG | interferon gamma | 3458 |
| ITK | ITK | IL2 inducible T-cell kinase | 3702 |
| THEMIS | THEMIS | thymocyte selection associated | 387357 |
| LCK | LCK | LCK proto-oncogene, Src family tyrosine kinase | 3932 |
| LCP2 | LCP2 | lymphocyte cytosolic protein 2 | 3937 |
| LYN | LYN | LYN proto-oncogene, Src family tyrosine kinase | 4067 |
| MEF2C | MEF2C | myocyte enhancer factor 2C | 4208 |
| TRAT1 | TRAT1 | T-cell receptor associated transmembrane adaptor 1 | 50852 |
| PIK3CD | PIK3CD | phosphatidylinositol-4,5-bisphosphate 3-kinase catalytic subunit delta | 5293 |
| PLCG2 | PLCG2 | phospholipase C gamma 2 | 5336 |
| PLSCR1 | PLSCR1 | phospholipid scramblase 1 | 5359 |
| LAX1 | LAX1 | lymphocyte transmembrane adaptor 1 | 54900 |
| PSMB8 | PSMB8 | proteasome subunit beta 8 | 5696 |
| PSMB9 | PSMB9 | proteasome subunit beta 9 | 5698 |
| PSMB10 | PSMB10 | proteasome subunit beta 10 | 5699 |
| PTPN6 | PTPN6 | protein tyrosine phosphatase, non-receptor type 6 | 5777 |
| SYK | SYK | spleen associated tyrosine kinase | 6850 |
| BTK | BTK | Bruton tyrosine kinase | 695 |
| C3AR1 | C3AR1 | complement C3a receptor 1 | 719 |
| VAV1 | VAV1 | vav guanine nucleotide exchange factor 1 | 7409 |
| WAS | WAS | Wiskott-Aldrich syndrome | 7454 |
| WIPF1 | WIPF1 | WAS/WASL interacting protein family member 1 | 7456 |
| LAT2 | LAT2 | linker for activation of T-cells family member 2 | 7462 |
| ZAP70 | ZAP70 | zeta chain of T-cell receptor associated protein kinase 70 | 7535 |
| SKAP1 | SKAP1 | src kinase associated phosphoprotein 1 | 8631 |
| CD3D | CD3D | CD3d molecule | 915 |
| CD3E | CD3E | CD3e molecule | 916 |
| CD247 | CD247 | CD247 molecule | 919 |
| CD4 | CD4 | CD4 molecule | 920 |
| CD38 | CD38 | CD38 molecule | 952 |
| CD79B | CD79B | CD79b molecule | 974 |
| TESPA1 | TESPA1 | thymocyte expressed, positive selection associated 1 | 9840 |

  
  

Download Table

| **ID:GO:0002443     &nbspName:leukocyte mediated immunity** | | | |
| --- | --- | --- | --- |
| C=280; O=41; E=5.1; R=8.04; PValue=0e+00; FDR=0e+00 | | | |
| userid | Gene Symbol | Gene Name | Entrez Gene |
| RASGRP1 | RASGRP1 | RAS guanyl releasing protein 1 | 10125 |
| CD96 | CD96 | CD96 molecule | 10225 |
| BTN3A3 | BTN3A3 | butyrophilin subfamily 3 member A3 | 10384 |
| CTSC | CTSC | cathepsin C | 1075 |
| CORO1A | CORO1A | coronin 1A | 11151 |
| NLRP3 | NLRP3 | NLR family pyrin domain containing 3 | 114548 |
| LAT | LAT | linker for activation of T-cells | 27040 |
| GZMB | GZMB | granzyme B | 3002 |
| HLA-E | HLA-E | major histocompatibility complex, class I, E | 3133 |
| IFNG | IFNG | interferon gamma | 3458 |
| IL7R | IL7R | interleukin 7 receptor | 3575 |
| IRF7 | IRF7 | interferon regulatory factor 7 | 3665 |
| LAG3 | LAG3 | lymphocyte activating 3 | 3902 |
| LYN | LYN | LYN proto-oncogene, Src family tyrosine kinase | 4067 |
| SH2D1A | SH2D1A | SH2 domain containing 1A | 4068 |
| TLR8 | TLR8 | toll like receptor 8 | 51311 |
| PIK3CD | PIK3CD | phosphatidylinositol-4,5-bisphosphate 3-kinase catalytic subunit delta | 5293 |
| PIK3CG | PIK3CG | phosphatidylinositol-4,5-bisphosphate 3-kinase catalytic subunit gamma | 5294 |
| SASH3 | SASH3 | SAM and SH3 domain containing 3 | 54440 |
| PTPN6 | PTPN6 | protein tyrosine phosphatase, non-receptor type 6 | 5777 |
| SLAMF7 | SLAMF7 | SLAM family member 7 | 57823 |
| RAC2 | RAC2 | ras-related C3 botulinum toxin substrate 2 (rho family, small GTP binding protein Rac2) | 5880 |
| SLAMF1 | SLAMF1 | signaling lymphocytic activation molecule family member 1 | 6504 |
| SYK | SYK | spleen associated tyrosine kinase | 6850 |
| BTK | BTK | Bruton tyrosine kinase | 695 |
| TLR4 | TLR4 | toll like receptor 4 | 7099 |
| SERPING1 | SERPING1 | serpin family G member 1 | 710 |
| C1QA | C1QA | complement C1q A chain | 712 |
| C1QB | C1QB | complement C1q B chain | 713 |
| C2 | C2 | complement C2 | 717 |
| CCR2 | CCR2 | C-C motif chemokine receptor 2 | 729230 |
| VAV1 | VAV1 | vav guanine nucleotide exchange factor 1 | 7409 |
| WAS | WAS | Wiskott-Aldrich syndrome | 7454 |
| LAT2 | LAT2 | linker for activation of T-cells family member 2 | 7462 |
| ZAP70 | ZAP70 | zeta chain of T-cell receptor associated protein kinase 70 | 7535 |
| HAVCR2 | HAVCR2 | hepatitis A virus cellular receptor 2 | 84868 |
| TNFSF13 | TNFSF13 | tumor necrosis factor superfamily member 13 | 8741 |
| RSAD2 | RSAD2 | radical S-adenosyl methionine domain containing 2 | 91543 |
| CD8A | CD8A | CD8a molecule | 925 |
| CD40LG | CD40LG | CD40 ligand | 959 |
| CD74 | CD74 | CD74 molecule | 972 |

  
  

Download Table

| **ID:GO:0002449     &nbspName:lymphocyte mediated immunity** | | | |
| --- | --- | --- | --- |
| C=210; O=31; E=3.82; R=8.11; PValue=0e+00; FDR=0e+00 | | | |
| userid | Gene Symbol | Gene Name | Entrez Gene |
| RASGRP1 | RASGRP1 | RAS guanyl releasing protein 1 | 10125 |
| CD96 | CD96 | CD96 molecule | 10225 |
| BTN3A3 | BTN3A3 | butyrophilin subfamily 3 member A3 | 10384 |
| CTSC | CTSC | cathepsin C | 1075 |
| CORO1A | CORO1A | coronin 1A | 11151 |
| NLRP3 | NLRP3 | NLR family pyrin domain containing 3 | 114548 |
| GZMB | GZMB | granzyme B | 3002 |
| HLA-E | HLA-E | major histocompatibility complex, class I, E | 3133 |
| IFNG | IFNG | interferon gamma | 3458 |
| IL7R | IL7R | interleukin 7 receptor | 3575 |
| IRF7 | IRF7 | interferon regulatory factor 7 | 3665 |
| LAG3 | LAG3 | lymphocyte activating 3 | 3902 |
| SH2D1A | SH2D1A | SH2 domain containing 1A | 4068 |
| TLR8 | TLR8 | toll like receptor 8 | 51311 |
| SASH3 | SASH3 | SAM and SH3 domain containing 3 | 54440 |
| PTPN6 | PTPN6 | protein tyrosine phosphatase, non-receptor type 6 | 5777 |
| SLAMF7 | SLAMF7 | SLAM family member 7 | 57823 |
| SLAMF1 | SLAMF1 | signaling lymphocytic activation molecule family member 1 | 6504 |
| BTK | BTK | Bruton tyrosine kinase | 695 |
| SERPING1 | SERPING1 | serpin family G member 1 | 710 |
| C1QA | C1QA | complement C1q A chain | 712 |
| C1QB | C1QB | complement C1q B chain | 713 |
| C2 | C2 | complement C2 | 717 |
| VAV1 | VAV1 | vav guanine nucleotide exchange factor 1 | 7409 |
| WAS | WAS | Wiskott-Aldrich syndrome | 7454 |
| HAVCR2 | HAVCR2 | hepatitis A virus cellular receptor 2 | 84868 |
| TNFSF13 | TNFSF13 | tumor necrosis factor superfamily member 13 | 8741 |
| RSAD2 | RSAD2 | radical S-adenosyl methionine domain containing 2 | 91543 |
| CD8A | CD8A | CD8a molecule | 925 |
| CD40LG | CD40LG | CD40 ligand | 959 |
| CD74 | CD74 | CD74 molecule | 972 |

  
  

Download Table

| **ID:GO:0002460     &nbspName:adaptive immune response based on somatic recombination of immune receptors built from immunoglobulin superfamily domains** | | | |
| --- | --- | --- | --- |
| C=212; O=33; E=3.86; R=8.55; PValue=0e+00; FDR=0e+00 | | | |
| userid | Gene Symbol | Gene Name | Entrez Gene |
| EBI3 | EBI3 | Epstein-Barr virus induced 3 | 10148 |
| BTN3A3 | BTN3A3 | butyrophilin subfamily 3 member A3 | 10384 |
| CXCL13 | CXCL13 | C-X-C motif chemokine ligand 13 | 10563 |
| TNFSF13B | TNFSF13B | tumor necrosis factor superfamily member 13b | 10673 |
| CTSC | CTSC | cathepsin C | 1075 |
| NLRP3 | NLRP3 | NLR family pyrin domain containing 3 | 114548 |
| HLA-E | HLA-E | major histocompatibility complex, class I, E | 3133 |
| IFNG | IFNG | interferon gamma | 3458 |
| IL7R | IL7R | interleukin 7 receptor | 3575 |
| IRF4 | IRF4 | interferon regulatory factor 4 | 3662 |
| IRF7 | IRF7 | interferon regulatory factor 7 | 3665 |
| LY9 | LY9 | lymphocyte antigen 9 | 4063 |
| MEF2C | MEF2C | myocyte enhancer factor 2C | 4208 |
| TLR8 | TLR8 | toll like receptor 8 | 51311 |
| SASH3 | SASH3 | SAM and SH3 domain containing 3 | 54440 |
| PTPN6 | PTPN6 | protein tyrosine phosphatase, non-receptor type 6 | 5777 |
| SLAMF1 | SLAMF1 | signaling lymphocytic activation molecule family member 1 | 6504 |
| BTK | BTK | Bruton tyrosine kinase | 695 |
| TLR4 | TLR4 | toll like receptor 4 | 7099 |
| SERPING1 | SERPING1 | serpin family G member 1 | 710 |
| C1QA | C1QA | complement C1q A chain | 712 |
| TNFAIP3 | TNFAIP3 | TNF alpha induced protein 3 | 7128 |
| C1QB | C1QB | complement C1q B chain | 713 |
| C2 | C2 | complement C2 | 717 |
| CCR2 | CCR2 | C-C motif chemokine receptor 2 | 729230 |
| WAS | WAS | Wiskott-Aldrich syndrome | 7454 |
| HAVCR2 | HAVCR2 | hepatitis A virus cellular receptor 2 | 84868 |
| TNFSF13 | TNFSF13 | tumor necrosis factor superfamily member 13 | 8741 |
| RSAD2 | RSAD2 | radical S-adenosyl methionine domain containing 2 | 91543 |
| CD4 | CD4 | CD4 molecule | 920 |
| CD8A | CD8A | CD8a molecule | 925 |
| CD40LG | CD40LG | CD40 ligand | 959 |
| CD74 | CD74 | CD74 molecule | 972 |

  
  

Download Table

| **ID:GO:0002520     &nbspName:immune system development** | | | |
| --- | --- | --- | --- |
| C=781; O=72; E=14.22; R=5.06; PValue=0e+00; FDR=0e+00 | | | |
| userid | Gene Symbol | Gene Name | Entrez Gene |
| RASGRP1 | RASGRP1 | RAS guanyl releasing protein 1 | 10125 |
| IKZF1 | IKZF1 | IKAROS family zinc finger 1 | 10320 |
| CXCL13 | CXCL13 | C-X-C motif chemokine ligand 13 | 10563 |
| LILRB4 | LILRB4 | leukocyte immunoglobulin like receptor B4 | 11006 |
| NLRP3 | NLRP3 | NLR family pyrin domain containing 3 | 114548 |
| BATF2 | BATF2 | basic leucine zipper ATF-like transcription factor 2 | 116071 |
| CCR1 | CCR1 | C-C motif chemokine receptor 1 | 1230 |
| CCR7 | CCR7 | C-C motif chemokine receptor 7 | 1236 |
| CSF1R | CSF1R | colony stimulating factor 1 receptor | 1436 |
| DOCK2 | DOCK2 | dedicator of cytokinesis 2 | 1794 |
| GPR183 | GPR183 | G protein-coupled receptor 183 | 1880 |
| ETS1 | ETS1 | ETS proto-oncogene 1, transcription factor | 2113 |
| PLCL2 | PLCL2 | phospholipase C like 2 | 23228 |
| FLT3LG | FLT3LG | fms related tyrosine kinase 3 ligand | 2323 |
| TNFRSF13B | TNFRSF13B | TNF receptor superfamily member 13B | 23495 |
| PTPN22 | PTPN22 | protein tyrosine phosphatase, non-receptor type 22 | 26191 |
| GPR18 | GPR18 | G protein-coupled receptor 18 | 2841 |
| HCLS1 | HCLS1 | hematopoietic cell-specific Lyn substrate 1 | 3059 |
| NCKAP1L | NCKAP1L | NCK associated protein 1 like | 3071 |
| HLA-G | HLA-G | major histocompatibility complex, class I, G | 3135 |
| IRF8 | IRF8 | interferon regulatory factor 8 | 3394 |
| IFI16 | IFI16 | interferon gamma inducible protein 16 | 3428 |
| IFNG | IFNG | interferon gamma | 3458 |
| IL2RA | IL2RA | interleukin 2 receptor subunit alpha | 3559 |
| IL7R | IL7R | interleukin 7 receptor | 3575 |
| IDO1 | IDO1 | indoleamine 2,3-dioxygenase 1 | 3620 |
| IRF1 | IRF1 | interferon regulatory factor 1 | 3659 |
| IRF4 | IRF4 | interferon regulatory factor 4 | 3662 |
| IRF7 | IRF7 | interferon regulatory factor 7 | 3665 |
| ITK | ITK | IL2 inducible T-cell kinase | 3702 |
| THEMIS | THEMIS | thymocyte selection associated | 387357 |
| LCK | LCK | LCK proto-oncogene, Src family tyrosine kinase | 3932 |
| LTB | LTB | lymphotoxin beta | 4050 |
| LY9 | LY9 | lymphocyte antigen 9 | 4063 |
| LYN | LYN | LYN proto-oncogene, Src family tyrosine kinase | 4067 |
| MEF2C | MEF2C | myocyte enhancer factor 2C | 4208 |
| MFNG | MFNG | MFNG O-fucosylpeptide 3-beta-N-acetylglucosaminyltransferase | 4242 |
| PIK3CD | PIK3CD | phosphatidylinositol-4,5-bisphosphate 3-kinase catalytic subunit delta | 5293 |
| PIP4K2A | PIP4K2A | phosphatidylinositol-5-phosphate 4-kinase type 2 alpha | 5305 |
| PLCG2 | PLCG2 | phospholipase C gamma 2 | 5336 |
| PLEK | PLEK | pleckstrin | 5341 |
| SASH3 | SASH3 | SAM and SH3 domain containing 3 | 54440 |
| HERC6 | HERC6 | HECT and RLD domain containing E3 ubiquitin protein ligase family member 6 | 55008 |
| PPP2R3C | PPP2R3C | protein phosphatase 2 regulatory subunit B''gamma | 55012 |
| TMEM176A | TMEM176A | transmembrane protein 176A | 55365 |
| PTGER4 | PTGER4 | prostaglandin E receptor 4 | 5734 |
| PTPN6 | PTPN6 | protein tyrosine phosphatase, non-receptor type 6 | 5777 |
| SLAMF1 | SLAMF1 | signaling lymphocytic activation molecule family member 1 | 6504 |
| SPI1 | SPI1 | Spi-1 proto-oncogene | 6688 |
| ZFP36L2 | ZFP36L2 | ZFP36 ring finger protein like 2 | 678 |
| SYK | SYK | spleen associated tyrosine kinase | 6850 |
| BTK | BTK | Bruton tyrosine kinase | 695 |
| TLR4 | TLR4 | toll like receptor 4 | 7099 |
| TNFAIP3 | TNFAIP3 | TNF alpha induced protein 3 | 7128 |
| TYROBP | TYROBP | TYRO protein tyrosine kinase binding protein | 7305 |
| VAV1 | VAV1 | vav guanine nucleotide exchange factor 1 | 7409 |
| ZAP70 | ZAP70 | zeta chain of T-cell receptor associated protein kinase 70 | 7535 |
| HAVCR2 | HAVCR2 | hepatitis A virus cellular receptor 2 | 84868 |
| TNFSF13 | TNFSF13 | tumor necrosis factor superfamily member 13 | 8741 |
| CD2 | CD2 | CD2 molecule | 914 |
| CD3D | CD3D | CD3d molecule | 915 |
| RSAD2 | RSAD2 | radical S-adenosyl methionine domain containing 2 | 91543 |
| CD3E | CD3E | CD3e molecule | 916 |
| CD4 | CD4 | CD4 molecule | 920 |
| CD8A | CD8A | CD8a molecule | 925 |
| CD83 | CD83 | CD83 molecule | 9308 |
| CD86 | CD86 | CD86 molecule | 942 |
| CD40LG | CD40LG | CD40 ligand | 959 |
| ISG15 | ISG15 | ISG15 ubiquitin-like modifier | 9636 |
| CD74 | CD74 | CD74 molecule | 972 |
| CD79B | CD79B | CD79b molecule | 974 |
| TESPA1 | TESPA1 | thymocyte expressed, positive selection associated 1 | 9840 |

  
  

Download Table

| **ID:GO:0002521     &nbspName:leukocyte differentiation** | | | |
| --- | --- | --- | --- |
| C=455; O=59; E=8.28; R=7.12; PValue=0e+00; FDR=0e+00 | | | |
| userid | Gene Symbol | Gene Name | Entrez Gene |
| RASGRP1 | RASGRP1 | RAS guanyl releasing protein 1 | 10125 |
| IKZF1 | IKZF1 | IKAROS family zinc finger 1 | 10320 |
| LILRB4 | LILRB4 | leukocyte immunoglobulin like receptor B4 | 11006 |
| NLRP3 | NLRP3 | NLR family pyrin domain containing 3 | 114548 |
| BATF2 | BATF2 | basic leucine zipper ATF-like transcription factor 2 | 116071 |
| CCR1 | CCR1 | C-C motif chemokine receptor 1 | 1230 |
| CCR7 | CCR7 | C-C motif chemokine receptor 7 | 1236 |
| CSF1R | CSF1R | colony stimulating factor 1 receptor | 1436 |
| DOCK2 | DOCK2 | dedicator of cytokinesis 2 | 1794 |
| GPR183 | GPR183 | G protein-coupled receptor 183 | 1880 |
| PLCL2 | PLCL2 | phospholipase C like 2 | 23228 |
| FLT3LG | FLT3LG | fms related tyrosine kinase 3 ligand | 2323 |
| PTPN22 | PTPN22 | protein tyrosine phosphatase, non-receptor type 22 | 26191 |
| GPR18 | GPR18 | G protein-coupled receptor 18 | 2841 |
| HCLS1 | HCLS1 | hematopoietic cell-specific Lyn substrate 1 | 3059 |
| NCKAP1L | NCKAP1L | NCK associated protein 1 like | 3071 |
| HLA-G | HLA-G | major histocompatibility complex, class I, G | 3135 |
| IFI16 | IFI16 | interferon gamma inducible protein 16 | 3428 |
| IFNG | IFNG | interferon gamma | 3458 |
| IL2RA | IL2RA | interleukin 2 receptor subunit alpha | 3559 |
| IL7R | IL7R | interleukin 7 receptor | 3575 |
| IRF1 | IRF1 | interferon regulatory factor 1 | 3659 |
| IRF4 | IRF4 | interferon regulatory factor 4 | 3662 |
| IRF7 | IRF7 | interferon regulatory factor 7 | 3665 |
| ITK | ITK | IL2 inducible T-cell kinase | 3702 |
| THEMIS | THEMIS | thymocyte selection associated | 387357 |
| LCK | LCK | LCK proto-oncogene, Src family tyrosine kinase | 3932 |
| LY9 | LY9 | lymphocyte antigen 9 | 4063 |
| LYN | LYN | LYN proto-oncogene, Src family tyrosine kinase | 4067 |
| MEF2C | MEF2C | myocyte enhancer factor 2C | 4208 |
| MFNG | MFNG | MFNG O-fucosylpeptide 3-beta-N-acetylglucosaminyltransferase | 4242 |
| PIK3CD | PIK3CD | phosphatidylinositol-4,5-bisphosphate 3-kinase catalytic subunit delta | 5293 |
| PLCG2 | PLCG2 | phospholipase C gamma 2 | 5336 |
| SASH3 | SASH3 | SAM and SH3 domain containing 3 | 54440 |
| PPP2R3C | PPP2R3C | protein phosphatase 2 regulatory subunit B''gamma | 55012 |
| TMEM176A | TMEM176A | transmembrane protein 176A | 55365 |
| PTGER4 | PTGER4 | prostaglandin E receptor 4 | 5734 |
| PTPN6 | PTPN6 | protein tyrosine phosphatase, non-receptor type 6 | 5777 |
| SLAMF1 | SLAMF1 | signaling lymphocytic activation molecule family member 1 | 6504 |
| SPI1 | SPI1 | Spi-1 proto-oncogene | 6688 |
| ZFP36L2 | ZFP36L2 | ZFP36 ring finger protein like 2 | 678 |
| SYK | SYK | spleen associated tyrosine kinase | 6850 |
| BTK | BTK | Bruton tyrosine kinase | 695 |
| TLR4 | TLR4 | toll like receptor 4 | 7099 |
| TYROBP | TYROBP | TYRO protein tyrosine kinase binding protein | 7305 |
| VAV1 | VAV1 | vav guanine nucleotide exchange factor 1 | 7409 |
| ZAP70 | ZAP70 | zeta chain of T-cell receptor associated protein kinase 70 | 7535 |
| CD2 | CD2 | CD2 molecule | 914 |
| CD3D | CD3D | CD3d molecule | 915 |
| RSAD2 | RSAD2 | radical S-adenosyl methionine domain containing 2 | 91543 |
| CD3E | CD3E | CD3e molecule | 916 |
| CD4 | CD4 | CD4 molecule | 920 |
| CD8A | CD8A | CD8a molecule | 925 |
| CD83 | CD83 | CD83 molecule | 9308 |
| CD86 | CD86 | CD86 molecule | 942 |
| CD40LG | CD40LG | CD40 ligand | 959 |
| CD74 | CD74 | CD74 molecule | 972 |
| CD79B | CD79B | CD79b molecule | 974 |
| TESPA1 | TESPA1 | thymocyte expressed, positive selection associated 1 | 9840 |

  
  

Download Table

| **ID:GO:0002682     &nbspName:regulation of immune system process** | | | |
| --- | --- | --- | --- |
| C=1265; O=145; E=23.03; R=6.3; PValue=0e+00; FDR=0e+00 | | | |
| userid | Gene Symbol | Gene Name | Entrez Gene |
| RASGRP1 | RASGRP1 | RAS guanyl releasing protein 1 | 10125 |
| EBI3 | EBI3 | Epstein-Barr virus induced 3 | 10148 |
| CD96 | CD96 | CD96 molecule | 10225 |
| CXCL13 | CXCL13 | C-X-C motif chemokine ligand 13 | 10563 |
| TNFSF13B | TNFSF13B | tumor necrosis factor superfamily member 13b | 10673 |
| HCST | HCST | hematopoietic cell signal transducer | 10870 |
| LILRB4 | LILRB4 | leukocyte immunoglobulin like receptor B4 | 11006 |
| BTN3A1 | BTN3A1 | butyrophilin subfamily 3 member A1 | 11119 |
| CORO1A | CORO1A | coronin 1A | 11151 |
| NLRP3 | NLRP3 | NLR family pyrin domain containing 3 | 114548 |
| CCR1 | CCR1 | C-C motif chemokine receptor 1 | 1230 |
| CCR7 | CCR7 | C-C motif chemokine receptor 7 | 1236 |
| OSCAR | OSCAR | osteoclast associated, immunoglobulin-like receptor | 126014 |
| CTSS | CTSS | cathepsin S | 1520 |
| DOCK2 | DOCK2 | dedicator of cytokinesis 2 | 1794 |
| GPR183 | GPR183 | G protein-coupled receptor 183 | 1880 |
| AIF1 | AIF1 | allograft inflammatory factor 1 | 199 |
| ETS1 | ETS1 | ETS proto-oncogene 1, transcription factor | 2113 |
| FCN1 | FCN1 | ficolin 1 | 2219 |
| PLCL2 | PLCL2 | phospholipase C like 2 | 23228 |
| FLT3LG | FLT3LG | fms related tyrosine kinase 3 ligand | 2323 |
| TNFRSF13B | TNFRSF13B | TNF receptor superfamily member 13B | 23495 |
| FYN | FYN | FYN proto-oncogene, Src family tyrosine kinase | 2534 |
| PTPN22 | PTPN22 | protein tyrosine phosphatase, non-receptor type 22 | 26191 |
| GBP1 | GBP1 | guanylate binding protein 1 | 2633 |
| LAT | LAT | linker for activation of T-cells | 27040 |
| CXCR3 | CXCR3 | C-X-C motif chemokine receptor 3 | 2833 |
| GPR18 | GPR18 | G protein-coupled receptor 18 | 2841 |
| HCK | HCK | HCK proto-oncogene, Src family tyrosine kinase | 3055 |
| HCLS1 | HCLS1 | hematopoietic cell-specific Lyn substrate 1 | 3059 |
| NCKAP1L | NCKAP1L | NCK associated protein 1 like | 3071 |
| HLA-DMB | HLA-DMB | major histocompatibility complex, class II, DM beta | 3109 |
| HLA-DOB | HLA-DOB | major histocompatibility complex, class II, DO beta | 3112 |
| HLA-DPA1 | HLA-DPA1 | major histocompatibility complex, class II, DP alpha 1 | 3113 |
| HLA-DPB1 | HLA-DPB1 | major histocompatibility complex, class II, DP beta 1 | 3115 |
| HLA-DRA | HLA-DRA | major histocompatibility complex, class II, DR alpha | 3122 |
| HLA-E | HLA-E | major histocompatibility complex, class I, E | 3133 |
| HLA-F | HLA-F | major histocompatibility complex, class I, F | 3134 |
| HLA-G | HLA-G | major histocompatibility complex, class I, G | 3135 |
| ICAM2 | ICAM2 | intercellular adhesion molecule 2 | 3384 |
| TMEM173 | TMEM173 | transmembrane protein 173 | 340061 |
| IFI16 | IFI16 | interferon gamma inducible protein 16 | 3428 |
| IFIT1 | IFIT1 | interferon induced protein with tetratricopeptide repeats 1 | 3434 |
| IFNG | IFNG | interferon gamma | 3458 |
| IL2RA | IL2RA | interleukin 2 receptor subunit alpha | 3559 |
| IL7R | IL7R | interleukin 7 receptor | 3575 |
| IDO1 | IDO1 | indoleamine 2,3-dioxygenase 1 | 3620 |
| CXCL10 | CXCL10 | C-X-C motif chemokine ligand 10 | 3627 |
| IRF1 | IRF1 | interferon regulatory factor 1 | 3659 |
| IRF4 | IRF4 | interferon regulatory factor 4 | 3662 |
| IRF7 | IRF7 | interferon regulatory factor 7 | 3665 |
| ITGAL | ITGAL | integrin subunit alpha L | 3683 |
| ITGB2 | ITGB2 | integrin subunit beta 2 | 3689 |
| ITGB7 | ITGB7 | integrin subunit beta 7 | 3695 |
| ITK | ITK | IL2 inducible T-cell kinase | 3702 |
| TBC1D10C | TBC1D10C | TBC1 domain family member 10C | 374403 |
| KIR2DL3 | KIR2DL3 | killer cell immunoglobulin like receptor, two Ig domains and long cytoplasmic tail 3 | 3804 |
| KLRB1 | KLRB1 | killer cell lectin like receptor B1 | 3820 |
| THEMIS | THEMIS | thymocyte selection associated | 387357 |
| LAG3 | LAG3 | lymphocyte activating 3 | 3902 |
| LCK | LCK | LCK proto-oncogene, Src family tyrosine kinase | 3932 |
| LCP2 | LCP2 | lymphocyte cytosolic protein 2 | 3937 |
| LYN | LYN | LYN proto-oncogene, Src family tyrosine kinase | 4067 |
| SH2D1A | SH2D1A | SH2 domain containing 1A | 4068 |
| MEF2C | MEF2C | myocyte enhancer factor 2C | 4208 |
| CXCL9 | CXCL9 | C-X-C motif chemokine ligand 9 | 4283 |
| MPP1 | MPP1 | membrane palmitoylated protein 1 | 4354 |
| TRAT1 | TRAT1 | T-cell receptor associated transmembrane adaptor 1 | 50852 |
| HERC5 | HERC5 | HECT and RLD domain containing E3 ubiquitin protein ligase 5 | 51191 |
| TLR7 | TLR7 | toll like receptor 7 | 51284 |
| TLR8 | TLR8 | toll like receptor 8 | 51311 |
| PIK3CD | PIK3CD | phosphatidylinositol-4,5-bisphosphate 3-kinase catalytic subunit delta | 5293 |
| PLCG2 | PLCG2 | phospholipase C gamma 2 | 5336 |
| PLSCR1 | PLSCR1 | phospholipid scramblase 1 | 5359 |
| SASH3 | SASH3 | SAM and SH3 domain containing 3 | 54440 |
| LAX1 | LAX1 | lymphocyte transmembrane adaptor 1 | 54900 |
| PPP2R3C | PPP2R3C | protein phosphatase 2 regulatory subunit B''gamma | 55012 |
| BANK1 | BANK1 | B-cell scaffold protein with ankyrin repeats 1 | 55024 |
| TAPBPL | TAPBPL | TAP binding protein like | 55080 |
| TMEM176A | TMEM176A | transmembrane protein 176A | 55365 |
| SIRPG | SIRPG | signal regulatory protein gamma | 55423 |
| DDX60 | DDX60 | DExD/H-box helicase 60 | 55601 |
| PSMB8 | PSMB8 | proteasome subunit beta 8 | 5696 |
| PSMB9 | PSMB9 | proteasome subunit beta 9 | 5698 |
| PSMB10 | PSMB10 | proteasome subunit beta 10 | 5699 |
| PTGER4 | PTGER4 | prostaglandin E receptor 4 | 5734 |
| PTPN6 | PTPN6 | protein tyrosine phosphatase, non-receptor type 6 | 5777 |
| SLAMF7 | SLAMF7 | SLAM family member 7 | 57823 |
| PTPRE | PTPRE | protein tyrosine phosphatase, receptor type E | 5791 |
| RAC2 | RAC2 | ras-related C3 botulinum toxin substrate 2 (rho family, small GTP binding protein Rac2) | 5880 |
| CCL2 | CCL2 | C-C motif chemokine ligand 2 | 6347 |
| CCL5 | CCL5 | C-C motif chemokine ligand 5 | 6352 |
| GPSM3 | GPSM3 | G-protein signaling modulator 3 | 63940 |
| SELL | SELL | selectin L | 6402 |
| SAMSN1 | SAMSN1 | SAM domain, SH3 domain and nuclear localization signals 1 | 64092 |
| SLAMF1 | SLAMF1 | signaling lymphocytic activation molecule family member 1 | 6504 |
| SPI1 | SPI1 | Spi-1 proto-oncogene | 6688 |
| STAT1 | STAT1 | signal transducer and activator of transcription 1 | 6772 |
| ZFP36L2 | ZFP36L2 | ZFP36 ring finger protein like 2 | 678 |
| SYK | SYK | spleen associated tyrosine kinase | 6850 |
| TAP1 | TAP1 | transporter 1, ATP binding cassette subfamily B member | 6890 |
| TAP2 | TAP2 | transporter 2, ATP binding cassette subfamily B member | 6891 |
| BTK | BTK | Bruton tyrosine kinase | 695 |
| TLR4 | TLR4 | toll like receptor 4 | 7099 |
| SERPING1 | SERPING1 | serpin family G member 1 | 710 |
| C1QA | C1QA | complement C1q A chain | 712 |
| TNFAIP3 | TNFAIP3 | TNF alpha induced protein 3 | 7128 |
| C1QB | C1QB | complement C1q B chain | 713 |
| C2 | C2 | complement C2 | 717 |
| C3AR1 | C3AR1 | complement C3a receptor 1 | 719 |
| CCR2 | CCR2 | C-C motif chemokine receptor 2 | 729230 |
| TNFRSF4 | TNFRSF4 | TNF receptor superfamily member 4 | 7293 |
| TYROBP | TYROBP | TYRO protein tyrosine kinase binding protein | 7305 |
| VAV1 | VAV1 | vav guanine nucleotide exchange factor 1 | 7409 |
| WAS | WAS | Wiskott-Aldrich syndrome | 7454 |
| WIPF1 | WIPF1 | WAS/WASL interacting protein family member 1 | 7456 |
| LAT2 | LAT2 | linker for activation of T-cells family member 2 | 7462 |
| ZAP70 | ZAP70 | zeta chain of T-cell receptor associated protein kinase 70 | 7535 |
| LST1 | LST1 | leukocyte specific transcript 1 | 7940 |
| PLVAP | PLVAP | plasmalemma vesicle associated protein | 83483 |
| HAVCR2 | HAVCR2 | hepatitis A virus cellular receptor 2 | 84868 |
| IFITM1 | IFITM1 | interferon induced transmembrane protein 1 | 8519 |
| SKAP1 | SKAP1 | src kinase associated phosphoprotein 1 | 8631 |
| TNFSF13 | TNFSF13 | tumor necrosis factor superfamily member 13 | 8741 |
| TNFRSF14 | TNFRSF14 | TNF receptor superfamily member 14 | 8764 |
| AP1S2 | AP1S2 | adaptor related protein complex 1 sigma 2 subunit | 8905 |
| CD2 | CD2 | CD2 molecule | 914 |
| CD3D | CD3D | CD3d molecule | 915 |
| RSAD2 | RSAD2 | radical S-adenosyl methionine domain containing 2 | 91543 |
| CD3E | CD3E | CD3e molecule | 916 |
| CD247 | CD247 | CD247 molecule | 919 |
| CD4 | CD4 | CD4 molecule | 920 |
| CD6 | CD6 | CD6 molecule | 923 |
| CD8A | CD8A | CD8a molecule | 925 |
| CD14 | CD14 | CD14 molecule | 929 |
| CD83 | CD83 | CD83 molecule | 9308 |
| CD86 | CD86 | CD86 molecule | 942 |
| AIM2 | AIM2 | absent in melanoma 2 | 9447 |
| CD38 | CD38 | CD38 molecule | 952 |
| CD40LG | CD40LG | CD40 ligand | 959 |
| CD48 | CD48 | CD48 molecule | 962 |
| ISG15 | ISG15 | ISG15 ubiquitin-like modifier | 9636 |
| CD74 | CD74 | CD74 molecule | 972 |
| CD79B | CD79B | CD79b molecule | 974 |
| TESPA1 | TESPA1 | thymocyte expressed, positive selection associated 1 | 9840 |

  
  

Download Table

| **ID:GO:0002683     &nbspName:negative regulation of immune system process** | | | |
| --- | --- | --- | --- |
| C=352; O=40; E=6.41; R=6.24; PValue=0e+00; FDR=0e+00 | | | |
| userid | Gene Symbol | Gene Name | Entrez Gene |
| CD96 | CD96 | CD96 molecule | 10225 |
| LILRB4 | LILRB4 | leukocyte immunoglobulin like receptor B4 | 11006 |
| PLCL2 | PLCL2 | phospholipase C like 2 | 23228 |
| TNFRSF13B | TNFRSF13B | TNF receptor superfamily member 13B | 23495 |
| PTPN22 | PTPN22 | protein tyrosine phosphatase, non-receptor type 22 | 26191 |
| GBP1 | GBP1 | guanylate binding protein 1 | 2633 |
| GPR18 | GPR18 | G protein-coupled receptor 18 | 2841 |
| HLA-DOB | HLA-DOB | major histocompatibility complex, class II, DO beta | 3112 |
| HLA-E | HLA-E | major histocompatibility complex, class I, E | 3133 |
| HLA-G | HLA-G | major histocompatibility complex, class I, G | 3135 |
| IFI16 | IFI16 | interferon gamma inducible protein 16 | 3428 |
| IFIT1 | IFIT1 | interferon induced protein with tetratricopeptide repeats 1 | 3434 |
| IL2RA | IL2RA | interleukin 2 receptor subunit alpha | 3559 |
| IL7R | IL7R | interleukin 7 receptor | 3575 |
| IDO1 | IDO1 | indoleamine 2,3-dioxygenase 1 | 3620 |
| IRF1 | IRF1 | interferon regulatory factor 1 | 3659 |
| IRF4 | IRF4 | interferon regulatory factor 4 | 3662 |
| TBC1D10C | TBC1D10C | TBC1 domain family member 10C | 374403 |
| LAG3 | LAG3 | lymphocyte activating 3 | 3902 |
| LYN | LYN | LYN proto-oncogene, Src family tyrosine kinase | 4067 |
| LAX1 | LAX1 | lymphocyte transmembrane adaptor 1 | 54900 |
| BANK1 | BANK1 | B-cell scaffold protein with ankyrin repeats 1 | 55024 |
| TAPBPL | TAPBPL | TAP binding protein like | 55080 |
| TMEM176A | TMEM176A | transmembrane protein 176A | 55365 |
| PTGER4 | PTGER4 | prostaglandin E receptor 4 | 5734 |
| PTPN6 | PTPN6 | protein tyrosine phosphatase, non-receptor type 6 | 5777 |
| CCL2 | CCL2 | C-C motif chemokine ligand 2 | 6347 |
| SAMSN1 | SAMSN1 | SAM domain, SH3 domain and nuclear localization signals 1 | 64092 |
| SLAMF1 | SLAMF1 | signaling lymphocytic activation molecule family member 1 | 6504 |
| BTK | BTK | Bruton tyrosine kinase | 695 |
| TLR4 | TLR4 | toll like receptor 4 | 7099 |
| SERPING1 | SERPING1 | serpin family G member 1 | 710 |
| TNFAIP3 | TNFAIP3 | TNF alpha induced protein 3 | 7128 |
| CCR2 | CCR2 | C-C motif chemokine receptor 2 | 729230 |
| LST1 | LST1 | leukocyte specific transcript 1 | 7940 |
| HAVCR2 | HAVCR2 | hepatitis A virus cellular receptor 2 | 84868 |
| TNFRSF14 | TNFRSF14 | TNF receptor superfamily member 14 | 8764 |
| CD14 | CD14 | CD14 molecule | 929 |
| CD86 | CD86 | CD86 molecule | 942 |
| CD74 | CD74 | CD74 molecule | 972 |

  
  

Download Table

| **ID:GO:0002684     &nbspName:positive regulation of immune system process** | | | |
| --- | --- | --- | --- |
| C=879; O=110; E=16; R=6.87; PValue=0e+00; FDR=0e+00 | | | |
| userid | Gene Symbol | Gene Name | Entrez Gene |
| RASGRP1 | RASGRP1 | RAS guanyl releasing protein 1 | 10125 |
| EBI3 | EBI3 | Epstein-Barr virus induced 3 | 10148 |
| CXCL13 | CXCL13 | C-X-C motif chemokine ligand 13 | 10563 |
| TNFSF13B | TNFSF13B | tumor necrosis factor superfamily member 13b | 10673 |
| BTN3A1 | BTN3A1 | butyrophilin subfamily 3 member A1 | 11119 |
| CORO1A | CORO1A | coronin 1A | 11151 |
| NLRP3 | NLRP3 | NLR family pyrin domain containing 3 | 114548 |
| CCR1 | CCR1 | C-C motif chemokine receptor 1 | 1230 |
| CCR7 | CCR7 | C-C motif chemokine receptor 7 | 1236 |
| CTSS | CTSS | cathepsin S | 1520 |
| GPR183 | GPR183 | G protein-coupled receptor 183 | 1880 |
| AIF1 | AIF1 | allograft inflammatory factor 1 | 199 |
| ETS1 | ETS1 | ETS proto-oncogene 1, transcription factor | 2113 |
| FCN1 | FCN1 | ficolin 1 | 2219 |
| PLCL2 | PLCL2 | phospholipase C like 2 | 23228 |
| FLT3LG | FLT3LG | fms related tyrosine kinase 3 ligand | 2323 |
| FYN | FYN | FYN proto-oncogene, Src family tyrosine kinase | 2534 |
| PTPN22 | PTPN22 | protein tyrosine phosphatase, non-receptor type 22 | 26191 |
| GBP1 | GBP1 | guanylate binding protein 1 | 2633 |
| LAT | LAT | linker for activation of T-cells | 27040 |
| HCK | HCK | HCK proto-oncogene, Src family tyrosine kinase | 3055 |
| HCLS1 | HCLS1 | hematopoietic cell-specific Lyn substrate 1 | 3059 |
| NCKAP1L | NCKAP1L | NCK associated protein 1 like | 3071 |
| HLA-DMB | HLA-DMB | major histocompatibility complex, class II, DM beta | 3109 |
| HLA-DPA1 | HLA-DPA1 | major histocompatibility complex, class II, DP alpha 1 | 3113 |
| HLA-DPB1 | HLA-DPB1 | major histocompatibility complex, class II, DP beta 1 | 3115 |
| HLA-DRA | HLA-DRA | major histocompatibility complex, class II, DR alpha | 3122 |
| HLA-E | HLA-E | major histocompatibility complex, class I, E | 3133 |
| HLA-G | HLA-G | major histocompatibility complex, class I, G | 3135 |
| ICAM2 | ICAM2 | intercellular adhesion molecule 2 | 3384 |
| TMEM173 | TMEM173 | transmembrane protein 173 | 340061 |
| IFI16 | IFI16 | interferon gamma inducible protein 16 | 3428 |
| IFNG | IFNG | interferon gamma | 3458 |
| IL2RA | IL2RA | interleukin 2 receptor subunit alpha | 3559 |
| IL7R | IL7R | interleukin 7 receptor | 3575 |
| IDO1 | IDO1 | indoleamine 2,3-dioxygenase 1 | 3620 |
| CXCL10 | CXCL10 | C-X-C motif chemokine ligand 10 | 3627 |
| IRF1 | IRF1 | interferon regulatory factor 1 | 3659 |
| IRF4 | IRF4 | interferon regulatory factor 4 | 3662 |
| IRF7 | IRF7 | interferon regulatory factor 7 | 3665 |
| ITGB2 | ITGB2 | integrin subunit beta 2 | 3689 |
| ITK | ITK | IL2 inducible T-cell kinase | 3702 |
| THEMIS | THEMIS | thymocyte selection associated | 387357 |
| LAG3 | LAG3 | lymphocyte activating 3 | 3902 |
| LCK | LCK | LCK proto-oncogene, Src family tyrosine kinase | 3932 |
| LCP2 | LCP2 | lymphocyte cytosolic protein 2 | 3937 |
| LYN | LYN | LYN proto-oncogene, Src family tyrosine kinase | 4067 |
| SH2D1A | SH2D1A | SH2 domain containing 1A | 4068 |
| MEF2C | MEF2C | myocyte enhancer factor 2C | 4208 |
| CXCL9 | CXCL9 | C-X-C motif chemokine ligand 9 | 4283 |
| TRAT1 | TRAT1 | T-cell receptor associated transmembrane adaptor 1 | 50852 |
| TLR7 | TLR7 | toll like receptor 7 | 51284 |
| TLR8 | TLR8 | toll like receptor 8 | 51311 |
| PIK3CD | PIK3CD | phosphatidylinositol-4,5-bisphosphate 3-kinase catalytic subunit delta | 5293 |
| PLCG2 | PLCG2 | phospholipase C gamma 2 | 5336 |
| PLSCR1 | PLSCR1 | phospholipid scramblase 1 | 5359 |
| SASH3 | SASH3 | SAM and SH3 domain containing 3 | 54440 |
| LAX1 | LAX1 | lymphocyte transmembrane adaptor 1 | 54900 |
| PPP2R3C | PPP2R3C | protein phosphatase 2 regulatory subunit B''gamma | 55012 |
| SIRPG | SIRPG | signal regulatory protein gamma | 55423 |
| DDX60 | DDX60 | DExD/H-box helicase 60 | 55601 |
| PSMB8 | PSMB8 | proteasome subunit beta 8 | 5696 |
| PSMB9 | PSMB9 | proteasome subunit beta 9 | 5698 |
| PSMB10 | PSMB10 | proteasome subunit beta 10 | 5699 |
| PTPN6 | PTPN6 | protein tyrosine phosphatase, non-receptor type 6 | 5777 |
| RAC2 | RAC2 | ras-related C3 botulinum toxin substrate 2 (rho family, small GTP binding protein Rac2) | 5880 |
| CCL2 | CCL2 | C-C motif chemokine ligand 2 | 6347 |
| CCL5 | CCL5 | C-C motif chemokine ligand 5 | 6352 |
| GPSM3 | GPSM3 | G-protein signaling modulator 3 | 63940 |
| SLAMF1 | SLAMF1 | signaling lymphocytic activation molecule family member 1 | 6504 |
| SYK | SYK | spleen associated tyrosine kinase | 6850 |
| TAP1 | TAP1 | transporter 1, ATP binding cassette subfamily B member | 6890 |
| TAP2 | TAP2 | transporter 2, ATP binding cassette subfamily B member | 6891 |
| BTK | BTK | Bruton tyrosine kinase | 695 |
| TLR4 | TLR4 | toll like receptor 4 | 7099 |
| SERPING1 | SERPING1 | serpin family G member 1 | 710 |
| C1QA | C1QA | complement C1q A chain | 712 |
| TNFAIP3 | TNFAIP3 | TNF alpha induced protein 3 | 7128 |
| C1QB | C1QB | complement C1q B chain | 713 |
| C2 | C2 | complement C2 | 717 |
| C3AR1 | C3AR1 | complement C3a receptor 1 | 719 |
| CCR2 | CCR2 | C-C motif chemokine receptor 2 | 729230 |
| TNFRSF4 | TNFRSF4 | TNF receptor superfamily member 4 | 7293 |
| VAV1 | VAV1 | vav guanine nucleotide exchange factor 1 | 7409 |
| WAS | WAS | Wiskott-Aldrich syndrome | 7454 |
| WIPF1 | WIPF1 | WAS/WASL interacting protein family member 1 | 7456 |
| LAT2 | LAT2 | linker for activation of T-cells family member 2 | 7462 |
| ZAP70 | ZAP70 | zeta chain of T-cell receptor associated protein kinase 70 | 7535 |
| PLVAP | PLVAP | plasmalemma vesicle associated protein | 83483 |
| HAVCR2 | HAVCR2 | hepatitis A virus cellular receptor 2 | 84868 |
| SKAP1 | SKAP1 | src kinase associated phosphoprotein 1 | 8631 |
| TNFSF13 | TNFSF13 | tumor necrosis factor superfamily member 13 | 8741 |
| TNFRSF14 | TNFRSF14 | TNF receptor superfamily member 14 | 8764 |
| CD2 | CD2 | CD2 molecule | 914 |
| CD3D | CD3D | CD3d molecule | 915 |
| RSAD2 | RSAD2 | radical S-adenosyl methionine domain containing 2 | 91543 |
| CD3E | CD3E | CD3e molecule | 916 |
| CD247 | CD247 | CD247 molecule | 919 |
| CD4 | CD4 | CD4 molecule | 920 |
| CD6 | CD6 | CD6 molecule | 923 |
| CD14 | CD14 | CD14 molecule | 929 |
| CD83 | CD83 | CD83 molecule | 9308 |
| CD86 | CD86 | CD86 molecule | 942 |
| AIM2 | AIM2 | absent in melanoma 2 | 9447 |
| CD38 | CD38 | CD38 molecule | 952 |
| CD40LG | CD40LG | CD40 ligand | 959 |
| ISG15 | ISG15 | ISG15 ubiquitin-like modifier | 9636 |
| CD74 | CD74 | CD74 molecule | 972 |
| CD79B | CD79B | CD79b molecule | 974 |
| TESPA1 | TESPA1 | thymocyte expressed, positive selection associated 1 | 9840 |

  
  

Download Table

| **ID:GO:0002694     &nbspName:regulation of leukocyte activation** | | | |
| --- | --- | --- | --- |
| C=439; O=69; E=7.99; R=8.63; PValue=0e+00; FDR=0e+00 | | | |
| userid | Gene Symbol | Gene Name | Entrez Gene |
| RASGRP1 | RASGRP1 | RAS guanyl releasing protein 1 | 10125 |
| EBI3 | EBI3 | Epstein-Barr virus induced 3 | 10148 |
| TNFSF13B | TNFSF13B | tumor necrosis factor superfamily member 13b | 10673 |
| CORO1A | CORO1A | coronin 1A | 11151 |
| NLRP3 | NLRP3 | NLR family pyrin domain containing 3 | 114548 |
| CCR7 | CCR7 | C-C motif chemokine receptor 7 | 1236 |
| GPR183 | GPR183 | G protein-coupled receptor 183 | 1880 |
| AIF1 | AIF1 | allograft inflammatory factor 1 | 199 |
| FLT3LG | FLT3LG | fms related tyrosine kinase 3 ligand | 2323 |
| TNFRSF13B | TNFRSF13B | TNF receptor superfamily member 13B | 23495 |
| FYN | FYN | FYN proto-oncogene, Src family tyrosine kinase | 2534 |
| PTPN22 | PTPN22 | protein tyrosine phosphatase, non-receptor type 22 | 26191 |
| LAT | LAT | linker for activation of T-cells | 27040 |
| NCKAP1L | NCKAP1L | NCK associated protein 1 like | 3071 |
| HLA-DMB | HLA-DMB | major histocompatibility complex, class II, DM beta | 3109 |
| HLA-DPA1 | HLA-DPA1 | major histocompatibility complex, class II, DP alpha 1 | 3113 |
| HLA-DPB1 | HLA-DPB1 | major histocompatibility complex, class II, DP beta 1 | 3115 |
| HLA-DRA | HLA-DRA | major histocompatibility complex, class II, DR alpha | 3122 |
| HLA-E | HLA-E | major histocompatibility complex, class I, E | 3133 |
| HLA-G | HLA-G | major histocompatibility complex, class I, G | 3135 |
| IFNG | IFNG | interferon gamma | 3458 |
| IL2RA | IL2RA | interleukin 2 receptor subunit alpha | 3559 |
| IL7R | IL7R | interleukin 7 receptor | 3575 |
| IDO1 | IDO1 | indoleamine 2,3-dioxygenase 1 | 3620 |
| IRF1 | IRF1 | interferon regulatory factor 1 | 3659 |
| IRF4 | IRF4 | interferon regulatory factor 4 | 3662 |
| TBC1D10C | TBC1D10C | TBC1 domain family member 10C | 374403 |
| LAG3 | LAG3 | lymphocyte activating 3 | 3902 |
| LCK | LCK | LCK proto-oncogene, Src family tyrosine kinase | 3932 |
| LYN | LYN | LYN proto-oncogene, Src family tyrosine kinase | 4067 |
| MEF2C | MEF2C | myocyte enhancer factor 2C | 4208 |
| PLSCR1 | PLSCR1 | phospholipid scramblase 1 | 5359 |
| SASH3 | SASH3 | SAM and SH3 domain containing 3 | 54440 |
| LAX1 | LAX1 | lymphocyte transmembrane adaptor 1 | 54900 |
| PPP2R3C | PPP2R3C | protein phosphatase 2 regulatory subunit B''gamma | 55012 |
| BANK1 | BANK1 | B-cell scaffold protein with ankyrin repeats 1 | 55024 |
| SIRPG | SIRPG | signal regulatory protein gamma | 55423 |
| PTPN6 | PTPN6 | protein tyrosine phosphatase, non-receptor type 6 | 5777 |
| PTPRE | PTPRE | protein tyrosine phosphatase, receptor type E | 5791 |
| RAC2 | RAC2 | ras-related C3 botulinum toxin substrate 2 (rho family, small GTP binding protein Rac2) | 5880 |
| CCL2 | CCL2 | C-C motif chemokine ligand 2 | 6347 |
| CCL5 | CCL5 | C-C motif chemokine ligand 5 | 6352 |
| SAMSN1 | SAMSN1 | SAM domain, SH3 domain and nuclear localization signals 1 | 64092 |
| SLAMF1 | SLAMF1 | signaling lymphocytic activation molecule family member 1 | 6504 |
| ZFP36L2 | ZFP36L2 | ZFP36 ring finger protein like 2 | 678 |
| SYK | SYK | spleen associated tyrosine kinase | 6850 |
| BTK | BTK | Bruton tyrosine kinase | 695 |
| TLR4 | TLR4 | toll like receptor 4 | 7099 |
| TNFAIP3 | TNFAIP3 | TNF alpha induced protein 3 | 7128 |
| CCR2 | CCR2 | C-C motif chemokine receptor 2 | 729230 |
| TNFRSF4 | TNFRSF4 | TNF receptor superfamily member 4 | 7293 |
| VAV1 | VAV1 | vav guanine nucleotide exchange factor 1 | 7409 |
| ZAP70 | ZAP70 | zeta chain of T-cell receptor associated protein kinase 70 | 7535 |
| LST1 | LST1 | leukocyte specific transcript 1 | 7940 |
| HAVCR2 | HAVCR2 | hepatitis A virus cellular receptor 2 | 84868 |
| TNFSF13 | TNFSF13 | tumor necrosis factor superfamily member 13 | 8741 |
| TNFRSF14 | TNFRSF14 | TNF receptor superfamily member 14 | 8764 |
| CD2 | CD2 | CD2 molecule | 914 |
| CD3D | CD3D | CD3d molecule | 915 |
| CD3E | CD3E | CD3e molecule | 916 |
| CD247 | CD247 | CD247 molecule | 919 |
| CD4 | CD4 | CD4 molecule | 920 |
| CD6 | CD6 | CD6 molecule | 923 |
| CD83 | CD83 | CD83 molecule | 9308 |
| CD86 | CD86 | CD86 molecule | 942 |
| CD38 | CD38 | CD38 molecule | 952 |
| CD40LG | CD40LG | CD40 ligand | 959 |
| CD74 | CD74 | CD74 molecule | 972 |
| TESPA1 | TESPA1 | thymocyte expressed, positive selection associated 1 | 9840 |

  
  

Download Table

| **ID:GO:0002696     &nbspName:positive regulation of leukocyte activation** | | | |
| --- | --- | --- | --- |
| C=279; O=52; E=5.08; R=10.24; PValue=0e+00; FDR=0e+00 | | | |
| userid | Gene Symbol | Gene Name | Entrez Gene |
| RASGRP1 | RASGRP1 | RAS guanyl releasing protein 1 | 10125 |
| EBI3 | EBI3 | Epstein-Barr virus induced 3 | 10148 |
| TNFSF13B | TNFSF13B | tumor necrosis factor superfamily member 13b | 10673 |
| CORO1A | CORO1A | coronin 1A | 11151 |
| NLRP3 | NLRP3 | NLR family pyrin domain containing 3 | 114548 |
| CCR7 | CCR7 | C-C motif chemokine receptor 7 | 1236 |
| GPR183 | GPR183 | G protein-coupled receptor 183 | 1880 |
| AIF1 | AIF1 | allograft inflammatory factor 1 | 199 |
| FLT3LG | FLT3LG | fms related tyrosine kinase 3 ligand | 2323 |
| FYN | FYN | FYN proto-oncogene, Src family tyrosine kinase | 2534 |
| NCKAP1L | NCKAP1L | NCK associated protein 1 like | 3071 |
| HLA-DMB | HLA-DMB | major histocompatibility complex, class II, DM beta | 3109 |
| HLA-DPA1 | HLA-DPA1 | major histocompatibility complex, class II, DP alpha 1 | 3113 |
| HLA-DPB1 | HLA-DPB1 | major histocompatibility complex, class II, DP beta 1 | 3115 |
| HLA-DRA | HLA-DRA | major histocompatibility complex, class II, DR alpha | 3122 |
| HLA-E | HLA-E | major histocompatibility complex, class I, E | 3133 |
| HLA-G | HLA-G | major histocompatibility complex, class I, G | 3135 |
| IFNG | IFNG | interferon gamma | 3458 |
| IL2RA | IL2RA | interleukin 2 receptor subunit alpha | 3559 |
| IL7R | IL7R | interleukin 7 receptor | 3575 |
| LCK | LCK | LCK proto-oncogene, Src family tyrosine kinase | 3932 |
| LYN | LYN | LYN proto-oncogene, Src family tyrosine kinase | 4067 |
| MEF2C | MEF2C | myocyte enhancer factor 2C | 4208 |
| SASH3 | SASH3 | SAM and SH3 domain containing 3 | 54440 |
| PPP2R3C | PPP2R3C | protein phosphatase 2 regulatory subunit B''gamma | 55012 |
| SIRPG | SIRPG | signal regulatory protein gamma | 55423 |
| PTPN6 | PTPN6 | protein tyrosine phosphatase, non-receptor type 6 | 5777 |
| CCL2 | CCL2 | C-C motif chemokine ligand 2 | 6347 |
| CCL5 | CCL5 | C-C motif chemokine ligand 5 | 6352 |
| SLAMF1 | SLAMF1 | signaling lymphocytic activation molecule family member 1 | 6504 |
| SYK | SYK | spleen associated tyrosine kinase | 6850 |
| BTK | BTK | Bruton tyrosine kinase | 695 |
| TLR4 | TLR4 | toll like receptor 4 | 7099 |
| CCR2 | CCR2 | C-C motif chemokine receptor 2 | 729230 |
| TNFRSF4 | TNFRSF4 | TNF receptor superfamily member 4 | 7293 |
| VAV1 | VAV1 | vav guanine nucleotide exchange factor 1 | 7409 |
| ZAP70 | ZAP70 | zeta chain of T-cell receptor associated protein kinase 70 | 7535 |
| HAVCR2 | HAVCR2 | hepatitis A virus cellular receptor 2 | 84868 |
| TNFSF13 | TNFSF13 | tumor necrosis factor superfamily member 13 | 8741 |
| TNFRSF14 | TNFRSF14 | TNF receptor superfamily member 14 | 8764 |
| CD2 | CD2 | CD2 molecule | 914 |
| CD3D | CD3D | CD3d molecule | 915 |
| CD3E | CD3E | CD3e molecule | 916 |
| CD247 | CD247 | CD247 molecule | 919 |
| CD4 | CD4 | CD4 molecule | 920 |
| CD6 | CD6 | CD6 molecule | 923 |
| CD83 | CD83 | CD83 molecule | 9308 |
| CD86 | CD86 | CD86 molecule | 942 |
| CD38 | CD38 | CD38 molecule | 952 |
| CD40LG | CD40LG | CD40 ligand | 959 |
| CD74 | CD74 | CD74 molecule | 972 |
| TESPA1 | TESPA1 | thymocyte expressed, positive selection associated 1 | 9840 |

  
  

Download Table

| **ID:GO:0002697     &nbspName:regulation of immune effector process** | | | |
| --- | --- | --- | --- |
| C=314; O=47; E=5.72; R=8.22; PValue=0e+00; FDR=0e+00 | | | |
| userid | Gene Symbol | Gene Name | Entrez Gene |
| RASGRP1 | RASGRP1 | RAS guanyl releasing protein 1 | 10125 |
| CD96 | CD96 | CD96 molecule | 10225 |
| NLRP3 | NLRP3 | NLR family pyrin domain containing 3 | 114548 |
| DOCK2 | DOCK2 | dedicator of cytokinesis 2 | 1794 |
| FYN | FYN | FYN proto-oncogene, Src family tyrosine kinase | 2534 |
| HCK | HCK | HCK proto-oncogene, Src family tyrosine kinase | 3055 |
| HLA-DMB | HLA-DMB | major histocompatibility complex, class II, DM beta | 3109 |
| HLA-E | HLA-E | major histocompatibility complex, class I, E | 3133 |
| TMEM173 | TMEM173 | transmembrane protein 173 | 340061 |
| IFIT1 | IFIT1 | interferon induced protein with tetratricopeptide repeats 1 | 3434 |
| IFNG | IFNG | interferon gamma | 3458 |
| IL2RA | IL2RA | interleukin 2 receptor subunit alpha | 3559 |
| IL7R | IL7R | interleukin 7 receptor | 3575 |
| IRF4 | IRF4 | interferon regulatory factor 4 | 3662 |
| LAG3 | LAG3 | lymphocyte activating 3 | 3902 |
| LCK | LCK | LCK proto-oncogene, Src family tyrosine kinase | 3932 |
| LYN | LYN | LYN proto-oncogene, Src family tyrosine kinase | 4067 |
| SH2D1A | SH2D1A | SH2 domain containing 1A | 4068 |
| HERC5 | HERC5 | HECT and RLD domain containing E3 ubiquitin protein ligase 5 | 51191 |
| SASH3 | SASH3 | SAM and SH3 domain containing 3 | 54440 |
| DDX60 | DDX60 | DExD/H-box helicase 60 | 55601 |
| PTPN6 | PTPN6 | protein tyrosine phosphatase, non-receptor type 6 | 5777 |
| RAC2 | RAC2 | ras-related C3 botulinum toxin substrate 2 (rho family, small GTP binding protein Rac2) | 5880 |
| SLAMF1 | SLAMF1 | signaling lymphocytic activation molecule family member 1 | 6504 |
| SYK | SYK | spleen associated tyrosine kinase | 6850 |
| BTK | BTK | Bruton tyrosine kinase | 695 |
| TLR4 | TLR4 | toll like receptor 4 | 7099 |
| SERPING1 | SERPING1 | serpin family G member 1 | 710 |
| TNFAIP3 | TNFAIP3 | TNF alpha induced protein 3 | 7128 |
| C2 | C2 | complement C2 | 717 |
| C3AR1 | C3AR1 | complement C3a receptor 1 | 719 |
| CCR2 | CCR2 | C-C motif chemokine receptor 2 | 729230 |
| TNFRSF4 | TNFRSF4 | TNF receptor superfamily member 4 | 7293 |
| VAV1 | VAV1 | vav guanine nucleotide exchange factor 1 | 7409 |
| WAS | WAS | Wiskott-Aldrich syndrome | 7454 |
| ZAP70 | ZAP70 | zeta chain of T-cell receptor associated protein kinase 70 | 7535 |
| HAVCR2 | HAVCR2 | hepatitis A virus cellular receptor 2 | 84868 |
| TNFSF13 | TNFSF13 | tumor necrosis factor superfamily member 13 | 8741 |
| TNFRSF14 | TNFRSF14 | TNF receptor superfamily member 14 | 8764 |
| AP1S2 | AP1S2 | adaptor related protein complex 1 sigma 2 subunit | 8905 |
| RSAD2 | RSAD2 | radical S-adenosyl methionine domain containing 2 | 91543 |
| CD247 | CD247 | CD247 molecule | 919 |
| CD4 | CD4 | CD4 molecule | 920 |
| CD86 | CD86 | CD86 molecule | 942 |
| AIM2 | AIM2 | absent in melanoma 2 | 9447 |
| CD40LG | CD40LG | CD40 ligand | 959 |
| CD74 | CD74 | CD74 molecule | 972 |

  
  

Download Table

| **ID:GO:0002757     &nbspName:immune response-activating signal transduction** | | | |
| --- | --- | --- | --- |
| C=428; O=60; E=7.79; R=7.7; PValue=0e+00; FDR=0e+00 | | | |
| userid | Gene Symbol | Gene Name | Entrez Gene |
| BTN3A1 | BTN3A1 | butyrophilin subfamily 3 member A1 | 11119 |
| CCR7 | CCR7 | C-C motif chemokine receptor 7 | 1236 |
| CTSS | CTSS | cathepsin S | 1520 |
| FCN1 | FCN1 | ficolin 1 | 2219 |
| PLCL2 | PLCL2 | phospholipase C like 2 | 23228 |
| FYN | FYN | FYN proto-oncogene, Src family tyrosine kinase | 2534 |
| PTPN22 | PTPN22 | protein tyrosine phosphatase, non-receptor type 22 | 26191 |
| GBP1 | GBP1 | guanylate binding protein 1 | 2633 |
| LAT | LAT | linker for activation of T-cells | 27040 |
| HCK | HCK | HCK proto-oncogene, Src family tyrosine kinase | 3055 |
| NCKAP1L | NCKAP1L | NCK associated protein 1 like | 3071 |
| HLA-DPA1 | HLA-DPA1 | major histocompatibility complex, class II, DP alpha 1 | 3113 |
| HLA-DPB1 | HLA-DPB1 | major histocompatibility complex, class II, DP beta 1 | 3115 |
| HLA-DRA | HLA-DRA | major histocompatibility complex, class II, DR alpha | 3122 |
| ICAM2 | ICAM2 | intercellular adhesion molecule 2 | 3384 |
| IFNG | IFNG | interferon gamma | 3458 |
| IRF1 | IRF1 | interferon regulatory factor 1 | 3659 |
| IRF4 | IRF4 | interferon regulatory factor 4 | 3662 |
| IRF7 | IRF7 | interferon regulatory factor 7 | 3665 |
| ITGB2 | ITGB2 | integrin subunit beta 2 | 3689 |
| ITK | ITK | IL2 inducible T-cell kinase | 3702 |
| THEMIS | THEMIS | thymocyte selection associated | 387357 |
| LCK | LCK | LCK proto-oncogene, Src family tyrosine kinase | 3932 |
| LCP2 | LCP2 | lymphocyte cytosolic protein 2 | 3937 |
| LYN | LYN | LYN proto-oncogene, Src family tyrosine kinase | 4067 |
| MEF2C | MEF2C | myocyte enhancer factor 2C | 4208 |
| TRAT1 | TRAT1 | T-cell receptor associated transmembrane adaptor 1 | 50852 |
| TLR7 | TLR7 | toll like receptor 7 | 51284 |
| TLR8 | TLR8 | toll like receptor 8 | 51311 |
| PIK3CD | PIK3CD | phosphatidylinositol-4,5-bisphosphate 3-kinase catalytic subunit delta | 5293 |
| PLCG2 | PLCG2 | phospholipase C gamma 2 | 5336 |
| PLSCR1 | PLSCR1 | phospholipid scramblase 1 | 5359 |
| LAX1 | LAX1 | lymphocyte transmembrane adaptor 1 | 54900 |
| DDX60 | DDX60 | DExD/H-box helicase 60 | 55601 |
| PSMB8 | PSMB8 | proteasome subunit beta 8 | 5696 |
| PSMB9 | PSMB9 | proteasome subunit beta 9 | 5698 |
| PSMB10 | PSMB10 | proteasome subunit beta 10 | 5699 |
| PTPN6 | PTPN6 | protein tyrosine phosphatase, non-receptor type 6 | 5777 |
| SYK | SYK | spleen associated tyrosine kinase | 6850 |
| BTK | BTK | Bruton tyrosine kinase | 695 |
| TLR4 | TLR4 | toll like receptor 4 | 7099 |
| TNFAIP3 | TNFAIP3 | TNF alpha induced protein 3 | 7128 |
| C3AR1 | C3AR1 | complement C3a receptor 1 | 719 |
| VAV1 | VAV1 | vav guanine nucleotide exchange factor 1 | 7409 |
| WAS | WAS | Wiskott-Aldrich syndrome | 7454 |
| WIPF1 | WIPF1 | WAS/WASL interacting protein family member 1 | 7456 |
| LAT2 | LAT2 | linker for activation of T-cells family member 2 | 7462 |
| ZAP70 | ZAP70 | zeta chain of T-cell receptor associated protein kinase 70 | 7535 |
| HAVCR2 | HAVCR2 | hepatitis A virus cellular receptor 2 | 84868 |
| SKAP1 | SKAP1 | src kinase associated phosphoprotein 1 | 8631 |
| CD3D | CD3D | CD3d molecule | 915 |
| RSAD2 | RSAD2 | radical S-adenosyl methionine domain containing 2 | 91543 |
| CD3E | CD3E | CD3e molecule | 916 |
| CD247 | CD247 | CD247 molecule | 919 |
| CD4 | CD4 | CD4 molecule | 920 |
| CD14 | CD14 | CD14 molecule | 929 |
| CD86 | CD86 | CD86 molecule | 942 |
| CD38 | CD38 | CD38 molecule | 952 |
| CD79B | CD79B | CD79b molecule | 974 |
| TESPA1 | TESPA1 | thymocyte expressed, positive selection associated 1 | 9840 |

  
  

Download Table

| **ID:GO:0002764     &nbspName:immune response-regulating signaling pathway** | | | |
| --- | --- | --- | --- |
| C=458; O=61; E=8.34; R=7.32; PValue=0e+00; FDR=0e+00 | | | |
| userid | Gene Symbol | Gene Name | Entrez Gene |
| BTN3A1 | BTN3A1 | butyrophilin subfamily 3 member A1 | 11119 |
| CCR7 | CCR7 | C-C motif chemokine receptor 7 | 1236 |
| CTSS | CTSS | cathepsin S | 1520 |
| FCN1 | FCN1 | ficolin 1 | 2219 |
| PLCL2 | PLCL2 | phospholipase C like 2 | 23228 |
| FYN | FYN | FYN proto-oncogene, Src family tyrosine kinase | 2534 |
| PTPN22 | PTPN22 | protein tyrosine phosphatase, non-receptor type 22 | 26191 |
| GBP1 | GBP1 | guanylate binding protein 1 | 2633 |
| LAT | LAT | linker for activation of T-cells | 27040 |
| HCK | HCK | HCK proto-oncogene, Src family tyrosine kinase | 3055 |
| NCKAP1L | NCKAP1L | NCK associated protein 1 like | 3071 |
| HLA-DPA1 | HLA-DPA1 | major histocompatibility complex, class II, DP alpha 1 | 3113 |
| HLA-DPB1 | HLA-DPB1 | major histocompatibility complex, class II, DP beta 1 | 3115 |
| HLA-DRA | HLA-DRA | major histocompatibility complex, class II, DR alpha | 3122 |
| HLA-G | HLA-G | major histocompatibility complex, class I, G | 3135 |
| ICAM2 | ICAM2 | intercellular adhesion molecule 2 | 3384 |
| IFNG | IFNG | interferon gamma | 3458 |
| IRF1 | IRF1 | interferon regulatory factor 1 | 3659 |
| IRF4 | IRF4 | interferon regulatory factor 4 | 3662 |
| IRF7 | IRF7 | interferon regulatory factor 7 | 3665 |
| ITGB2 | ITGB2 | integrin subunit beta 2 | 3689 |
| ITK | ITK | IL2 inducible T-cell kinase | 3702 |
| THEMIS | THEMIS | thymocyte selection associated | 387357 |
| LCK | LCK | LCK proto-oncogene, Src family tyrosine kinase | 3932 |
| LCP2 | LCP2 | lymphocyte cytosolic protein 2 | 3937 |
| LYN | LYN | LYN proto-oncogene, Src family tyrosine kinase | 4067 |
| MEF2C | MEF2C | myocyte enhancer factor 2C | 4208 |
| TRAT1 | TRAT1 | T-cell receptor associated transmembrane adaptor 1 | 50852 |
| TLR7 | TLR7 | toll like receptor 7 | 51284 |
| TLR8 | TLR8 | toll like receptor 8 | 51311 |
| PIK3CD | PIK3CD | phosphatidylinositol-4,5-bisphosphate 3-kinase catalytic subunit delta | 5293 |
| PLCG2 | PLCG2 | phospholipase C gamma 2 | 5336 |
| PLSCR1 | PLSCR1 | phospholipid scramblase 1 | 5359 |
| LAX1 | LAX1 | lymphocyte transmembrane adaptor 1 | 54900 |
| DDX60 | DDX60 | DExD/H-box helicase 60 | 55601 |
| PSMB8 | PSMB8 | proteasome subunit beta 8 | 5696 |
| PSMB9 | PSMB9 | proteasome subunit beta 9 | 5698 |
| PSMB10 | PSMB10 | proteasome subunit beta 10 | 5699 |
| PTPN6 | PTPN6 | protein tyrosine phosphatase, non-receptor type 6 | 5777 |
| SYK | SYK | spleen associated tyrosine kinase | 6850 |
| BTK | BTK | Bruton tyrosine kinase | 695 |
| TLR4 | TLR4 | toll like receptor 4 | 7099 |
| TNFAIP3 | TNFAIP3 | TNF alpha induced protein 3 | 7128 |
| C3AR1 | C3AR1 | complement C3a receptor 1 | 719 |
| VAV1 | VAV1 | vav guanine nucleotide exchange factor 1 | 7409 |
| WAS | WAS | Wiskott-Aldrich syndrome | 7454 |
| WIPF1 | WIPF1 | WAS/WASL interacting protein family member 1 | 7456 |
| LAT2 | LAT2 | linker for activation of T-cells family member 2 | 7462 |
| ZAP70 | ZAP70 | zeta chain of T-cell receptor associated protein kinase 70 | 7535 |
| HAVCR2 | HAVCR2 | hepatitis A virus cellular receptor 2 | 84868 |
| SKAP1 | SKAP1 | src kinase associated phosphoprotein 1 | 8631 |
| CD3D | CD3D | CD3d molecule | 915 |
| RSAD2 | RSAD2 | radical S-adenosyl methionine domain containing 2 | 91543 |
| CD3E | CD3E | CD3e molecule | 916 |
| CD247 | CD247 | CD247 molecule | 919 |
| CD4 | CD4 | CD4 molecule | 920 |
| CD14 | CD14 | CD14 molecule | 929 |
| CD86 | CD86 | CD86 molecule | 942 |
| CD38 | CD38 | CD38 molecule | 952 |
| CD79B | CD79B | CD79b molecule | 974 |
| TESPA1 | TESPA1 | thymocyte expressed, positive selection associated 1 | 9840 |

  
  

Download Table

| **ID:GO:0002768     &nbspName:immune response-regulating cell surface receptor signaling pathway** | | | |
| --- | --- | --- | --- |
| C=328; O=47; E=5.97; R=7.87; PValue=0e+00; FDR=0e+00 | | | |
| userid | Gene Symbol | Gene Name | Entrez Gene |
| BTN3A1 | BTN3A1 | butyrophilin subfamily 3 member A1 | 11119 |
| CCR7 | CCR7 | C-C motif chemokine receptor 7 | 1236 |
| FCN1 | FCN1 | ficolin 1 | 2219 |
| PLCL2 | PLCL2 | phospholipase C like 2 | 23228 |
| FYN | FYN | FYN proto-oncogene, Src family tyrosine kinase | 2534 |
| PTPN22 | PTPN22 | protein tyrosine phosphatase, non-receptor type 22 | 26191 |
| GBP1 | GBP1 | guanylate binding protein 1 | 2633 |
| LAT | LAT | linker for activation of T-cells | 27040 |
| HCK | HCK | HCK proto-oncogene, Src family tyrosine kinase | 3055 |
| NCKAP1L | NCKAP1L | NCK associated protein 1 like | 3071 |
| HLA-DPA1 | HLA-DPA1 | major histocompatibility complex, class II, DP alpha 1 | 3113 |
| HLA-DPB1 | HLA-DPB1 | major histocompatibility complex, class II, DP beta 1 | 3115 |
| HLA-DRA | HLA-DRA | major histocompatibility complex, class II, DR alpha | 3122 |
| HLA-G | HLA-G | major histocompatibility complex, class I, G | 3135 |
| ICAM2 | ICAM2 | intercellular adhesion molecule 2 | 3384 |
| IFNG | IFNG | interferon gamma | 3458 |
| ITK | ITK | IL2 inducible T-cell kinase | 3702 |
| THEMIS | THEMIS | thymocyte selection associated | 387357 |
| LCK | LCK | LCK proto-oncogene, Src family tyrosine kinase | 3932 |
| LCP2 | LCP2 | lymphocyte cytosolic protein 2 | 3937 |
| LYN | LYN | LYN proto-oncogene, Src family tyrosine kinase | 4067 |
| MEF2C | MEF2C | myocyte enhancer factor 2C | 4208 |
| TRAT1 | TRAT1 | T-cell receptor associated transmembrane adaptor 1 | 50852 |
| PIK3CD | PIK3CD | phosphatidylinositol-4,5-bisphosphate 3-kinase catalytic subunit delta | 5293 |
| PLCG2 | PLCG2 | phospholipase C gamma 2 | 5336 |
| PLSCR1 | PLSCR1 | phospholipid scramblase 1 | 5359 |
| LAX1 | LAX1 | lymphocyte transmembrane adaptor 1 | 54900 |
| PSMB8 | PSMB8 | proteasome subunit beta 8 | 5696 |
| PSMB9 | PSMB9 | proteasome subunit beta 9 | 5698 |
| PSMB10 | PSMB10 | proteasome subunit beta 10 | 5699 |
| PTPN6 | PTPN6 | protein tyrosine phosphatase, non-receptor type 6 | 5777 |
| SYK | SYK | spleen associated tyrosine kinase | 6850 |
| BTK | BTK | Bruton tyrosine kinase | 695 |
| C3AR1 | C3AR1 | complement C3a receptor 1 | 719 |
| VAV1 | VAV1 | vav guanine nucleotide exchange factor 1 | 7409 |
| WAS | WAS | Wiskott-Aldrich syndrome | 7454 |
| WIPF1 | WIPF1 | WAS/WASL interacting protein family member 1 | 7456 |
| LAT2 | LAT2 | linker for activation of T-cells family member 2 | 7462 |
| ZAP70 | ZAP70 | zeta chain of T-cell receptor associated protein kinase 70 | 7535 |
| SKAP1 | SKAP1 | src kinase associated phosphoprotein 1 | 8631 |
| CD3D | CD3D | CD3d molecule | 915 |
| CD3E | CD3E | CD3e molecule | 916 |
| CD247 | CD247 | CD247 molecule | 919 |
| CD4 | CD4 | CD4 molecule | 920 |
| CD38 | CD38 | CD38 molecule | 952 |
| CD79B | CD79B | CD79b molecule | 974 |
| TESPA1 | TESPA1 | thymocyte expressed, positive selection associated 1 | 9840 |

  
  

Download Table

| **ID:GO:0006952     &nbspName:defense response** | | | |
| --- | --- | --- | --- |
| C=1424; O=135; E=25.93; R=5.21; PValue=0e+00; FDR=0e+00 | | | |
| userid | Gene Symbol | Gene Name | Entrez Gene |
| RASGRP1 | RASGRP1 | RAS guanyl releasing protein 1 | 10125 |
| KLRG1 | KLRG1 | killer cell lectin like receptor G1 | 10219 |
| CD96 | CD96 | CD96 molecule | 10225 |
| TRIM22 | TRIM22 | tripartite motif containing 22 | 10346 |
| IFITM3 | IFITM3 | interferon induced transmembrane protein 3 | 10410 |
| CLEC10A | CLEC10A | C-type lectin domain family 10 member A | 10462 |
| CXCL13 | CXCL13 | C-X-C motif chemokine ligand 13 | 10563 |
| GNLY | GNLY | granulysin | 10578 |
| IFITM2 | IFITM2 | interferon induced transmembrane protein 2 | 10581 |
| CXCR6 | CXCR6 | C-X-C motif chemokine receptor 6 | 10663 |
| IFI44L | IFI44L | interferon induced protein 44 like | 10964 |
| CORO1A | CORO1A | coronin 1A | 11151 |
| SP140 | SP140 | SP140 nuclear body protein | 11262 |
| NLRP3 | NLRP3 | NLR family pyrin domain containing 3 | 114548 |
| BATF2 | BATF2 | basic leucine zipper ATF-like transcription factor 2 | 116071 |
| TAGAP | TAGAP | T-cell activation RhoGTPase activating protein | 117289 |
| CCR1 | CCR1 | C-C motif chemokine receptor 1 | 1230 |
| CCR7 | CCR7 | C-C motif chemokine receptor 7 | 1236 |
| CSF1R | CSF1R | colony stimulating factor 1 receptor | 1436 |
| CTSS | CTSS | cathepsin S | 1520 |
| DOCK2 | DOCK2 | dedicator of cytokinesis 2 | 1794 |
| AIF1 | AIF1 | allograft inflammatory factor 1 | 199 |
| ETS1 | ETS1 | ETS proto-oncogene 1, transcription factor | 2113 |
| FCN1 | FCN1 | ficolin 1 | 2219 |
| SYT11 | SYT11 | synaptotagmin 11 | 23208 |
| FYN | FYN | FYN proto-oncogene, Src family tyrosine kinase | 2534 |
| PTPN22 | PTPN22 | protein tyrosine phosphatase, non-receptor type 22 | 26191 |
| GBP1 | GBP1 | guanylate binding protein 1 | 2633 |
| GBP2 | GBP2 | guanylate binding protein 2 | 2634 |
| LAT | LAT | linker for activation of T-cells | 27040 |
| XCR1 | XCR1 | X-C motif chemokine receptor 1 | 2829 |
| CXCR3 | CXCR3 | C-X-C motif chemokine receptor 3 | 2833 |
| GZMB | GZMB | granzyme B | 3002 |
| HCK | HCK | HCK proto-oncogene, Src family tyrosine kinase | 3055 |
| HLA-DPA1 | HLA-DPA1 | major histocompatibility complex, class II, DP alpha 1 | 3113 |
| HLA-DPB1 | HLA-DPB1 | major histocompatibility complex, class II, DP beta 1 | 3115 |
| HLA-DRA | HLA-DRA | major histocompatibility complex, class II, DR alpha | 3122 |
| AOAH | AOAH | acyloxyacyl hydrolase | 313 |
| HLA-E | HLA-E | major histocompatibility complex, class I, E | 3133 |
| HLA-F | HLA-F | major histocompatibility complex, class I, F | 3134 |
| HLA-G | HLA-G | major histocompatibility complex, class I, G | 3135 |
| ICAM2 | ICAM2 | intercellular adhesion molecule 2 | 3384 |
| IRF8 | IRF8 | interferon regulatory factor 8 | 3394 |
| TMEM173 | TMEM173 | transmembrane protein 173 | 340061 |
| IFI16 | IFI16 | interferon gamma inducible protein 16 | 3428 |
| IFI27 | IFI27 | interferon alpha inducible protein 27 | 3429 |
| IFI35 | IFI35 | interferon induced protein 35 | 3430 |
| IFIT1 | IFIT1 | interferon induced protein with tetratricopeptide repeats 1 | 3434 |
| IFIT3 | IFIT3 | interferon induced protein with tetratricopeptide repeats 3 | 3437 |
| IFNG | IFNG | interferon gamma | 3458 |
| APOE | APOE | apolipoprotein E | 348 |
| IL2RA | IL2RA | interleukin 2 receptor subunit alpha | 3559 |
| IDO1 | IDO1 | indoleamine 2,3-dioxygenase 1 | 3620 |
| CXCL10 | CXCL10 | C-X-C motif chemokine ligand 10 | 3627 |
| IRF1 | IRF1 | interferon regulatory factor 1 | 3659 |
| IRF4 | IRF4 | interferon regulatory factor 4 | 3662 |
| IRF7 | IRF7 | interferon regulatory factor 7 | 3665 |
| ITGAL | ITGAL | integrin subunit alpha L | 3683 |
| ITGB2 | ITGB2 | integrin subunit beta 2 | 3689 |
| ITK | ITK | IL2 inducible T-cell kinase | 3702 |
| LAG3 | LAG3 | lymphocyte activating 3 | 3902 |
| LCK | LCK | LCK proto-oncogene, Src family tyrosine kinase | 3932 |
| LY9 | LY9 | lymphocyte antigen 9 | 4063 |
| LYN | LYN | LYN proto-oncogene, Src family tyrosine kinase | 4067 |
| SH2D1A | SH2D1A | SH2 domain containing 1A | 4068 |
| LYZ | LYZ | lysozyme | 4069 |
| MEF2C | MEF2C | myocyte enhancer factor 2C | 4208 |
| CIITA | CIITA | class II major histocompatibility complex transactivator | 4261 |
| CXCL9 | CXCL9 | C-X-C motif chemokine ligand 9 | 4283 |
| MX1 | MX1 | MX dynamin like GTPase 1 | 4599 |
| MX2 | MX2 | MX dynamin like GTPase 2 | 4600 |
| OAS1 | OAS1 | 2'-5'-oligoadenylate synthetase 1 | 4938 |
| OAS2 | OAS2 | 2'-5'-oligoadenylate synthetase 2 | 4939 |
| OAS3 | OAS3 | 2'-5'-oligoadenylate synthetase 3 | 4940 |
| TRAT1 | TRAT1 | T-cell receptor associated transmembrane adaptor 1 | 50852 |
| HERC5 | HERC5 | HECT and RLD domain containing E3 ubiquitin protein ligase 5 | 51191 |
| CLEC1A | CLEC1A | C-type lectin domain family 1 member A | 51267 |
| TLR7 | TLR7 | toll like receptor 7 | 51284 |
| TLR8 | TLR8 | toll like receptor 8 | 51311 |
| PLAC8 | PLAC8 | placenta specific 8 | 51316 |
| PIK3CD | PIK3CD | phosphatidylinositol-4,5-bisphosphate 3-kinase catalytic subunit delta | 5293 |
| PIK3CG | PIK3CG | phosphatidylinositol-4,5-bisphosphate 3-kinase catalytic subunit gamma | 5294 |
| PLCG2 | PLCG2 | phospholipase C gamma 2 | 5336 |
| PLSCR1 | PLSCR1 | phospholipid scramblase 1 | 5359 |
| PRF1 | PRF1 | perforin 1 | 5551 |
| DDX60 | DDX60 | DExD/H-box helicase 60 | 55601 |
| PSMB8 | PSMB8 | proteasome subunit beta 8 | 5696 |
| PSMB9 | PSMB9 | proteasome subunit beta 9 | 5698 |
| PSMB10 | PSMB10 | proteasome subunit beta 10 | 5699 |
| PTGER4 | PTGER4 | prostaglandin E receptor 4 | 5734 |
| PTPN6 | PTPN6 | protein tyrosine phosphatase, non-receptor type 6 | 5777 |
| SLAMF7 | SLAMF7 | SLAM family member 7 | 57823 |
| RNASE6 | RNASE6 | ribonuclease A family member k6 | 6039 |
| CCL2 | CCL2 | C-C motif chemokine ligand 2 | 6347 |
| CCL5 | CCL5 | C-C motif chemokine ligand 5 | 6352 |
| GPSM3 | GPSM3 | G-protein signaling modulator 3 | 63940 |
| SLAMF1 | SLAMF1 | signaling lymphocytic activation molecule family member 1 | 6504 |
| STAT1 | STAT1 | signal transducer and activator of transcription 1 | 6772 |
| SYK | SYK | spleen associated tyrosine kinase | 6850 |
| TAP1 | TAP1 | transporter 1, ATP binding cassette subfamily B member | 6890 |
| BTK | BTK | Bruton tyrosine kinase | 695 |
| TLR4 | TLR4 | toll like receptor 4 | 7099 |
| SERPING1 | SERPING1 | serpin family G member 1 | 710 |
| C1QA | C1QA | complement C1q A chain | 712 |
| TNFAIP3 | TNFAIP3 | TNF alpha induced protein 3 | 7128 |
| C1QB | C1QB | complement C1q B chain | 713 |
| TNFRSF1B | TNFRSF1B | TNF receptor superfamily member 1B | 7133 |
| C2 | C2 | complement C2 | 717 |
| C3AR1 | C3AR1 | complement C3a receptor 1 | 719 |
| CCR2 | CCR2 | C-C motif chemokine receptor 2 | 729230 |
| TNFRSF4 | TNFRSF4 | TNF receptor superfamily member 4 | 7293 |
| TYROBP | TYROBP | TYRO protein tyrosine kinase binding protein | 7305 |
| VAV1 | VAV1 | vav guanine nucleotide exchange factor 1 | 7409 |
| WAS | WAS | Wiskott-Aldrich syndrome | 7454 |
| ZAP70 | ZAP70 | zeta chain of T-cell receptor associated protein kinase 70 | 7535 |
| HAVCR2 | HAVCR2 | hepatitis A virus cellular receptor 2 | 84868 |
| IFITM1 | IFITM1 | interferon induced transmembrane protein 1 | 8519 |
| OASL | OASL | 2'-5'-oligoadenylate synthetase like | 8638 |
| TNFRSF14 | TNFRSF14 | TNF receptor superfamily member 14 | 8764 |
| AP1S2 | AP1S2 | adaptor related protein complex 1 sigma 2 subunit | 8905 |
| PSTPIP1 | PSTPIP1 | proline-serine-threonine phosphatase interacting protein 1 | 9051 |
| RSAD2 | RSAD2 | radical S-adenosyl methionine domain containing 2 | 91543 |
| CD247 | CD247 | CD247 molecule | 919 |
| CD4 | CD4 | CD4 molecule | 920 |
| CD6 | CD6 | CD6 molecule | 923 |
| IL32 | IL32 | interleukin 32 | 9235 |
| CD8A | CD8A | CD8a molecule | 925 |
| CD14 | CD14 | CD14 molecule | 929 |
| CD83 | CD83 | CD83 molecule | 9308 |
| CD86 | CD86 | CD86 molecule | 942 |
| AIM2 | AIM2 | absent in melanoma 2 | 9447 |
| CD40LG | CD40LG | CD40 ligand | 959 |
| CD48 | CD48 | CD48 molecule | 962 |
| ISG15 | ISG15 | ISG15 ubiquitin-like modifier | 9636 |
| CD74 | CD74 | CD74 molecule | 972 |

  
  

Download Table

| **ID:GO:0006954     &nbspName:inflammatory response** | | | |
| --- | --- | --- | --- |
| C=648; O=54; E=11.8; R=4.58; PValue=0e+00; FDR=0e+00 | | | |
| userid | Gene Symbol | Gene Name | Entrez Gene |
| RASGRP1 | RASGRP1 | RAS guanyl releasing protein 1 | 10125 |
| KLRG1 | KLRG1 | killer cell lectin like receptor G1 | 10219 |
| CD96 | CD96 | CD96 molecule | 10225 |
| CXCL13 | CXCL13 | C-X-C motif chemokine ligand 13 | 10563 |
| CXCR6 | CXCR6 | C-X-C motif chemokine receptor 6 | 10663 |
| NLRP3 | NLRP3 | NLR family pyrin domain containing 3 | 114548 |
| CCR1 | CCR1 | C-C motif chemokine receptor 1 | 1230 |
| CCR7 | CCR7 | C-C motif chemokine receptor 7 | 1236 |
| CSF1R | CSF1R | colony stimulating factor 1 receptor | 1436 |
| AIF1 | AIF1 | allograft inflammatory factor 1 | 199 |
| ETS1 | ETS1 | ETS proto-oncogene 1, transcription factor | 2113 |
| LAT | LAT | linker for activation of T-cells | 27040 |
| XCR1 | XCR1 | X-C motif chemokine receptor 1 | 2829 |
| CXCR3 | CXCR3 | C-X-C motif chemokine receptor 3 | 2833 |
| HCK | HCK | HCK proto-oncogene, Src family tyrosine kinase | 3055 |
| AOAH | AOAH | acyloxyacyl hydrolase | 313 |
| IFI16 | IFI16 | interferon gamma inducible protein 16 | 3428 |
| APOE | APOE | apolipoprotein E | 348 |
| IL2RA | IL2RA | interleukin 2 receptor subunit alpha | 3559 |
| IDO1 | IDO1 | indoleamine 2,3-dioxygenase 1 | 3620 |
| CXCL10 | CXCL10 | C-X-C motif chemokine ligand 10 | 3627 |
| ITGAL | ITGAL | integrin subunit alpha L | 3683 |
| ITGB2 | ITGB2 | integrin subunit beta 2 | 3689 |
| LYN | LYN | LYN proto-oncogene, Src family tyrosine kinase | 4067 |
| LYZ | LYZ | lysozyme | 4069 |
| CXCL9 | CXCL9 | C-X-C motif chemokine ligand 9 | 4283 |
| TLR7 | TLR7 | toll like receptor 7 | 51284 |
| TLR8 | TLR8 | toll like receptor 8 | 51311 |
| PIK3CD | PIK3CD | phosphatidylinositol-4,5-bisphosphate 3-kinase catalytic subunit delta | 5293 |
| PIK3CG | PIK3CG | phosphatidylinositol-4,5-bisphosphate 3-kinase catalytic subunit gamma | 5294 |
| PLSCR1 | PLSCR1 | phospholipid scramblase 1 | 5359 |
| PTGER4 | PTGER4 | prostaglandin E receptor 4 | 5734 |
| CCL2 | CCL2 | C-C motif chemokine ligand 2 | 6347 |
| CCL5 | CCL5 | C-C motif chemokine ligand 5 | 6352 |
| GPSM3 | GPSM3 | G-protein signaling modulator 3 | 63940 |
| SLAMF1 | SLAMF1 | signaling lymphocytic activation molecule family member 1 | 6504 |
| SYK | SYK | spleen associated tyrosine kinase | 6850 |
| BTK | BTK | Bruton tyrosine kinase | 695 |
| TLR4 | TLR4 | toll like receptor 4 | 7099 |
| SERPING1 | SERPING1 | serpin family G member 1 | 710 |
| TNFAIP3 | TNFAIP3 | TNF alpha induced protein 3 | 7128 |
| TNFRSF1B | TNFRSF1B | TNF receptor superfamily member 1B | 7133 |
| C2 | C2 | complement C2 | 717 |
| C3AR1 | C3AR1 | complement C3a receptor 1 | 719 |
| CCR2 | CCR2 | C-C motif chemokine receptor 2 | 729230 |
| TNFRSF4 | TNFRSF4 | TNF receptor superfamily member 4 | 7293 |
| ZAP70 | ZAP70 | zeta chain of T-cell receptor associated protein kinase 70 | 7535 |
| HAVCR2 | HAVCR2 | hepatitis A virus cellular receptor 2 | 84868 |
| TNFRSF14 | TNFRSF14 | TNF receptor superfamily member 14 | 8764 |
| PSTPIP1 | PSTPIP1 | proline-serine-threonine phosphatase interacting protein 1 | 9051 |
| CD6 | CD6 | CD6 molecule | 923 |
| CD14 | CD14 | CD14 molecule | 929 |
| AIM2 | AIM2 | absent in melanoma 2 | 9447 |
| CD40LG | CD40LG | CD40 ligand | 959 |

  
  

Download Table

| **ID:GO:0006955     &nbspName:immune response** | | | |
| --- | --- | --- | --- |
| C=1450; O=177; E=26.4; R=6.7; PValue=0e+00; FDR=0e+00 | | | |
| userid | Gene Symbol | Gene Name | Entrez Gene |
| RASGRP1 | RASGRP1 | RAS guanyl releasing protein 1 | 10125 |
| EBI3 | EBI3 | Epstein-Barr virus induced 3 | 10148 |
| KLRG1 | KLRG1 | killer cell lectin like receptor G1 | 10219 |
| CD96 | CD96 | CD96 molecule | 10225 |
| IGSF6 | IGSF6 | immunoglobulin superfamily member 6 | 10261 |
| TRIM22 | TRIM22 | tripartite motif containing 22 | 10346 |
| BTN3A3 | BTN3A3 | butyrophilin subfamily 3 member A3 | 10384 |
| IFITM3 | IFITM3 | interferon induced transmembrane protein 3 | 10410 |
| CLEC10A | CLEC10A | C-type lectin domain family 10 member A | 10462 |
| SEMA4D | SEMA4D | semaphorin 4D | 10507 |
| CXCL13 | CXCL13 | C-X-C motif chemokine ligand 13 | 10563 |
| IFITM2 | IFITM2 | interferon induced transmembrane protein 2 | 10581 |
| TNFSF13B | TNFSF13B | tumor necrosis factor superfamily member 13b | 10673 |
| CTSC | CTSC | cathepsin C | 1075 |
| HCST | HCST | hematopoietic cell signal transducer | 10870 |
| LILRB4 | LILRB4 | leukocyte immunoglobulin like receptor B4 | 11006 |
| BTN3A1 | BTN3A1 | butyrophilin subfamily 3 member A1 | 11119 |
| CORO1A | CORO1A | coronin 1A | 11151 |
| NLRP3 | NLRP3 | NLR family pyrin domain containing 3 | 114548 |
| CCR1 | CCR1 | C-C motif chemokine receptor 1 | 1230 |
| CCR7 | CCR7 | C-C motif chemokine receptor 7 | 1236 |
| OSCAR | OSCAR | osteoclast associated, immunoglobulin-like receptor | 126014 |
| CSF1R | CSF1R | colony stimulating factor 1 receptor | 1436 |
| CTSS | CTSS | cathepsin S | 1520 |
| CTSW | CTSW | cathepsin W | 1521 |
| DOCK2 | DOCK2 | dedicator of cytokinesis 2 | 1794 |
| GPR183 | GPR183 | G protein-coupled receptor 183 | 1880 |
| AIF1 | AIF1 | allograft inflammatory factor 1 | 199 |
| ETS1 | ETS1 | ETS proto-oncogene 1, transcription factor | 2113 |
| FCN1 | FCN1 | ficolin 1 | 2219 |
| PLCL2 | PLCL2 | phospholipase C like 2 | 23228 |
| TNFRSF13B | TNFRSF13B | TNF receptor superfamily member 13B | 23495 |
| FYN | FYN | FYN proto-oncogene, Src family tyrosine kinase | 2534 |
| PTPN22 | PTPN22 | protein tyrosine phosphatase, non-receptor type 22 | 26191 |
| GBP1 | GBP1 | guanylate binding protein 1 | 2633 |
| GBP2 | GBP2 | guanylate binding protein 2 | 2634 |
| LAT | LAT | linker for activation of T-cells | 27040 |
| LAMP3 | LAMP3 | lysosomal associated membrane protein 3 | 27074 |
| ADAMDEC1 | ADAMDEC1 | ADAM like decysin 1 | 27299 |
| VPREB3 | VPREB3 | pre-B lymphocyte 3 | 29802 |
| GZMH | GZMH | granzyme H | 2999 |
| GZMA | GZMA | granzyme A | 3001 |
| GZMB | GZMB | granzyme B | 3002 |
| HCK | HCK | HCK proto-oncogene, Src family tyrosine kinase | 3055 |
| NCKAP1L | NCKAP1L | NCK associated protein 1 like | 3071 |
| HLA-DMB | HLA-DMB | major histocompatibility complex, class II, DM beta | 3109 |
| HLA-DOB | HLA-DOB | major histocompatibility complex, class II, DO beta | 3112 |
| HLA-DPA1 | HLA-DPA1 | major histocompatibility complex, class II, DP alpha 1 | 3113 |
| HLA-DPB1 | HLA-DPB1 | major histocompatibility complex, class II, DP beta 1 | 3115 |
| HLA-DRA | HLA-DRA | major histocompatibility complex, class II, DR alpha | 3122 |
| HLA-E | HLA-E | major histocompatibility complex, class I, E | 3133 |
| HLA-F | HLA-F | major histocompatibility complex, class I, F | 3134 |
| HLA-G | HLA-G | major histocompatibility complex, class I, G | 3135 |
| ICAM2 | ICAM2 | intercellular adhesion molecule 2 | 3384 |
| IRF8 | IRF8 | interferon regulatory factor 8 | 3394 |
| TMEM173 | TMEM173 | transmembrane protein 173 | 340061 |
| IFI16 | IFI16 | interferon gamma inducible protein 16 | 3428 |
| IFI27 | IFI27 | interferon alpha inducible protein 27 | 3429 |
| IFI35 | IFI35 | interferon induced protein 35 | 3430 |
| IFIT1 | IFIT1 | interferon induced protein with tetratricopeptide repeats 1 | 3434 |
| IFIT3 | IFIT3 | interferon induced protein with tetratricopeptide repeats 3 | 3437 |
| IFNG | IFNG | interferon gamma | 3458 |
| IL2RA | IL2RA | interleukin 2 receptor subunit alpha | 3559 |
| FASLG | FASLG | Fas ligand | 356 |
| IL2RG | IL2RG | interleukin 2 receptor subunit gamma | 3561 |
| IL7R | IL7R | interleukin 7 receptor | 3575 |
| IDO1 | IDO1 | indoleamine 2,3-dioxygenase 1 | 3620 |
| CXCL10 | CXCL10 | C-X-C motif chemokine ligand 10 | 3627 |
| IRF1 | IRF1 | interferon regulatory factor 1 | 3659 |
| IRF4 | IRF4 | interferon regulatory factor 4 | 3662 |
| IRF7 | IRF7 | interferon regulatory factor 7 | 3665 |
| ITGAL | ITGAL | integrin subunit alpha L | 3683 |
| ITGB2 | ITGB2 | integrin subunit beta 2 | 3689 |
| ITGB7 | ITGB7 | integrin subunit beta 7 | 3695 |
| ITK | ITK | IL2 inducible T-cell kinase | 3702 |
| KIR2DL3 | KIR2DL3 | killer cell immunoglobulin like receptor, two Ig domains and long cytoplasmic tail 3 | 3804 |
| KLRB1 | KLRB1 | killer cell lectin like receptor B1 | 3820 |
| THEMIS | THEMIS | thymocyte selection associated | 387357 |
| LAG3 | LAG3 | lymphocyte activating 3 | 3902 |
| LCK | LCK | LCK proto-oncogene, Src family tyrosine kinase | 3932 |
| LCP1 | LCP1 | lymphocyte cytosolic protein 1 | 3936 |
| LCP2 | LCP2 | lymphocyte cytosolic protein 2 | 3937 |
| LTB | LTB | lymphotoxin beta | 4050 |
| LY9 | LY9 | lymphocyte antigen 9 | 4063 |
| LYN | LYN | LYN proto-oncogene, Src family tyrosine kinase | 4067 |
| SH2D1A | SH2D1A | SH2 domain containing 1A | 4068 |
| MBP | MBP | myelin basic protein | 4155 |
| MEF2C | MEF2C | myocyte enhancer factor 2C | 4208 |
| MFNG | MFNG | MFNG O-fucosylpeptide 3-beta-N-acetylglucosaminyltransferase | 4242 |
| CIITA | CIITA | class II major histocompatibility complex transactivator | 4261 |
| CXCL9 | CXCL9 | C-X-C motif chemokine ligand 9 | 4283 |
| MX1 | MX1 | MX dynamin like GTPase 1 | 4599 |
| MX2 | MX2 | MX dynamin like GTPase 2 | 4600 |
| OAS1 | OAS1 | 2'-5'-oligoadenylate synthetase 1 | 4938 |
| OAS2 | OAS2 | 2'-5'-oligoadenylate synthetase 2 | 4939 |
| OAS3 | OAS3 | 2'-5'-oligoadenylate synthetase 3 | 4940 |
| TRAT1 | TRAT1 | T-cell receptor associated transmembrane adaptor 1 | 50852 |
| HERC5 | HERC5 | HECT and RLD domain containing E3 ubiquitin protein ligase 5 | 51191 |
| TLR7 | TLR7 | toll like receptor 7 | 51284 |
| TLR8 | TLR8 | toll like receptor 8 | 51311 |
| ENPP2 | ENPP2 | ectonucleotide pyrophosphatase/phosphodiesterase 2 | 5168 |
| PIK3CD | PIK3CD | phosphatidylinositol-4,5-bisphosphate 3-kinase catalytic subunit delta | 5293 |
| PIK3CG | PIK3CG | phosphatidylinositol-4,5-bisphosphate 3-kinase catalytic subunit gamma | 5294 |
| PLCG2 | PLCG2 | phospholipase C gamma 2 | 5336 |
| PLSCR1 | PLSCR1 | phospholipid scramblase 1 | 5359 |
| SASH3 | SASH3 | SAM and SH3 domain containing 3 | 54440 |
| APBB1IP | APBB1IP | amyloid beta precursor protein binding family B member 1 interacting protein | 54518 |
| LAX1 | LAX1 | lymphocyte transmembrane adaptor 1 | 54900 |
| PPP2R3C | PPP2R3C | protein phosphatase 2 regulatory subunit B''gamma | 55012 |
| MARCH1 | MARCH1 | membrane associated ring-CH-type finger 1 | 55016 |
| PRF1 | PRF1 | perforin 1 | 5551 |
| DDX60 | DDX60 | DExD/H-box helicase 60 | 55601 |
| PSMB8 | PSMB8 | proteasome subunit beta 8 | 5696 |
| PSMB9 | PSMB9 | proteasome subunit beta 9 | 5698 |
| PSMB10 | PSMB10 | proteasome subunit beta 10 | 5699 |
| PTGER4 | PTGER4 | prostaglandin E receptor 4 | 5734 |
| PTPN6 | PTPN6 | protein tyrosine phosphatase, non-receptor type 6 | 5777 |
| SLAMF7 | SLAMF7 | SLAM family member 7 | 57823 |
| RAC2 | RAC2 | ras-related C3 botulinum toxin substrate 2 (rho family, small GTP binding protein Rac2) | 5880 |
| TNFRSF17 | TNFRSF17 | TNF receptor superfamily member 17 | 608 |
| CCL2 | CCL2 | C-C motif chemokine ligand 2 | 6347 |
| CCL5 | CCL5 | C-C motif chemokine ligand 5 | 6352 |
| SELL | SELL | selectin L | 6402 |
| SAMSN1 | SAMSN1 | SAM domain, SH3 domain and nuclear localization signals 1 | 64092 |
| SLAMF1 | SLAMF1 | signaling lymphocytic activation molecule family member 1 | 6504 |
| STAT1 | STAT1 | signal transducer and activator of transcription 1 | 6772 |
| SYK | SYK | spleen associated tyrosine kinase | 6850 |
| TAP1 | TAP1 | transporter 1, ATP binding cassette subfamily B member | 6890 |
| TAP2 | TAP2 | transporter 2, ATP binding cassette subfamily B member | 6891 |
| TAPBP | TAPBP | TAP binding protein | 6892 |
| BTK | BTK | Bruton tyrosine kinase | 695 |
| TLR4 | TLR4 | toll like receptor 4 | 7099 |
| SERPING1 | SERPING1 | serpin family G member 1 | 710 |
| C1QA | C1QA | complement C1q A chain | 712 |
| TNFAIP3 | TNFAIP3 | TNF alpha induced protein 3 | 7128 |
| C1QB | C1QB | complement C1q B chain | 713 |
| TNFRSF1B | TNFRSF1B | TNF receptor superfamily member 1B | 7133 |
| C2 | C2 | complement C2 | 717 |
| C3AR1 | C3AR1 | complement C3a receptor 1 | 719 |
| CCR2 | CCR2 | C-C motif chemokine receptor 2 | 729230 |
| TNFRSF4 | TNFRSF4 | TNF receptor superfamily member 4 | 7293 |
| TYROBP | TYROBP | TYRO protein tyrosine kinase binding protein | 7305 |
| VAV1 | VAV1 | vav guanine nucleotide exchange factor 1 | 7409 |
| WAS | WAS | Wiskott-Aldrich syndrome | 7454 |
| WIPF1 | WIPF1 | WAS/WASL interacting protein family member 1 | 7456 |
| LAT2 | LAT2 | linker for activation of T-cells family member 2 | 7462 |
| ZAP70 | ZAP70 | zeta chain of T-cell receptor associated protein kinase 70 | 7535 |
| LST1 | LST1 | leukocyte specific transcript 1 | 7940 |
| HAVCR2 | HAVCR2 | hepatitis A virus cellular receptor 2 | 84868 |
| IFITM1 | IFITM1 | interferon induced transmembrane protein 1 | 8519 |
| CST7 | CST7 | cystatin F | 8530 |
| SKAP1 | SKAP1 | src kinase associated phosphoprotein 1 | 8631 |
| OASL | OASL | 2'-5'-oligoadenylate synthetase like | 8638 |
| S1PR4 | S1PR4 | sphingosine-1-phosphate receptor 4 | 8698 |
| TNFSF13 | TNFSF13 | tumor necrosis factor superfamily member 13 | 8741 |
| TNFRSF14 | TNFRSF14 | TNF receptor superfamily member 14 | 8764 |
| PSTPIP1 | PSTPIP1 | proline-serine-threonine phosphatase interacting protein 1 | 9051 |
| CD3D | CD3D | CD3d molecule | 915 |
| RSAD2 | RSAD2 | radical S-adenosyl methionine domain containing 2 | 91543 |
| CD3E | CD3E | CD3e molecule | 916 |
| CD247 | CD247 | CD247 molecule | 919 |
| CD4 | CD4 | CD4 molecule | 920 |
| CD6 | CD6 | CD6 molecule | 923 |
| IL32 | IL32 | interleukin 32 | 9235 |
| CD7 | CD7 | CD7 molecule | 924 |
| CD8A | CD8A | CD8a molecule | 925 |
| CD14 | CD14 | CD14 molecule | 929 |
| CD83 | CD83 | CD83 molecule | 9308 |
| CD86 | CD86 | CD86 molecule | 942 |
| AIM2 | AIM2 | absent in melanoma 2 | 9447 |
| CD38 | CD38 | CD38 molecule | 952 |
| CD40LG | CD40LG | CD40 ligand | 959 |
| CD48 | CD48 | CD48 molecule | 962 |
| ISG15 | ISG15 | ISG15 ubiquitin-like modifier | 9636 |
| CD74 | CD74 | CD74 molecule | 972 |
| CD79B | CD79B | CD79b molecule | 974 |
| TESPA1 | TESPA1 | thymocyte expressed, positive selection associated 1 | 9840 |

  
  

Download Table

| **ID:GO:0007155     &nbspName:cell adhesion** | | | |
| --- | --- | --- | --- |
| C=1667; O=98; E=30.35; R=3.23; PValue=0e+00; FDR=0e+00 | | | |
| userid | Gene Symbol | Gene Name | Entrez Gene |
| RASGRP1 | RASGRP1 | RAS guanyl releasing protein 1 | 10125 |
| EBI3 | EBI3 | Epstein-Barr virus induced 3 | 10148 |
| CD96 | CD96 | CD96 molecule | 10225 |
| SEMA4D | SEMA4D | semaphorin 4D | 10507 |
| CXCL13 | CXCL13 | C-X-C motif chemokine ligand 13 | 10563 |
| TNFSF13B | TNFSF13B | tumor necrosis factor superfamily member 13b | 10673 |
| BTN3A1 | BTN3A1 | butyrophilin subfamily 3 member A1 | 11119 |
| CORO1A | CORO1A | coronin 1A | 11151 |
| NLRP3 | NLRP3 | NLR family pyrin domain containing 3 | 114548 |
| CCR1 | CCR1 | C-C motif chemokine receptor 1 | 1230 |
| CCR7 | CCR7 | C-C motif chemokine receptor 7 | 1236 |
| SIRPA | SIRPA | signal regulatory protein alpha | 140885 |
| HAPLN3 | HAPLN3 | hyaluronan and proteoglycan link protein 3 | 145864 |
| DOCK2 | DOCK2 | dedicator of cytokinesis 2 | 1794 |
| GPR183 | GPR183 | G protein-coupled receptor 183 | 1880 |
| S1PR1 | S1PR1 | sphingosine-1-phosphate receptor 1 | 1901 |
| AIF1 | AIF1 | allograft inflammatory factor 1 | 199 |
| ETS1 | ETS1 | ETS proto-oncogene 1, transcription factor | 2113 |
| FYN | FYN | FYN proto-oncogene, Src family tyrosine kinase | 2534 |
| ABI3BP | ABI3BP | ABI family member 3 binding protein | 25890 |
| PTPN22 | PTPN22 | protein tyrosine phosphatase, non-receptor type 22 | 26191 |
| GBP1 | GBP1 | guanylate binding protein 1 | 2633 |
| LAT | LAT | linker for activation of T-cells | 27040 |
| ADAMDEC1 | ADAMDEC1 | ADAM like decysin 1 | 27299 |
| CXCR3 | CXCR3 | C-X-C motif chemokine receptor 3 | 2833 |
| GPR18 | GPR18 | G protein-coupled receptor 18 | 2841 |
| HCK | HCK | HCK proto-oncogene, Src family tyrosine kinase | 3055 |
| NCKAP1L | NCKAP1L | NCK associated protein 1 like | 3071 |
| HLA-DMB | HLA-DMB | major histocompatibility complex, class II, DM beta | 3109 |
| HLA-DPA1 | HLA-DPA1 | major histocompatibility complex, class II, DP alpha 1 | 3113 |
| HLA-DPB1 | HLA-DPB1 | major histocompatibility complex, class II, DP beta 1 | 3115 |
| HLA-DRA | HLA-DRA | major histocompatibility complex, class II, DR alpha | 3122 |
| HLA-E | HLA-E | major histocompatibility complex, class I, E | 3133 |
| HLA-G | HLA-G | major histocompatibility complex, class I, G | 3135 |
| ICAM2 | ICAM2 | intercellular adhesion molecule 2 | 3384 |
| IFNG | IFNG | interferon gamma | 3458 |
| IL2RA | IL2RA | interleukin 2 receptor subunit alpha | 3559 |
| IL7R | IL7R | interleukin 7 receptor | 3575 |
| IDO1 | IDO1 | indoleamine 2,3-dioxygenase 1 | 3620 |
| IRF1 | IRF1 | interferon regulatory factor 1 | 3659 |
| IRF4 | IRF4 | interferon regulatory factor 4 | 3662 |
| ITGAL | ITGAL | integrin subunit alpha L | 3683 |
| ITGAX | ITGAX | integrin subunit alpha X | 3687 |
| ITGB2 | ITGB2 | integrin subunit beta 2 | 3689 |
| ITGB7 | ITGB7 | integrin subunit beta 7 | 3695 |
| ITK | ITK | IL2 inducible T-cell kinase | 3702 |
| THEMIS | THEMIS | thymocyte selection associated | 387357 |
| LAG3 | LAG3 | lymphocyte activating 3 | 3902 |
| LCK | LCK | LCK proto-oncogene, Src family tyrosine kinase | 3932 |
| LCP1 | LCP1 | lymphocyte cytosolic protein 1 | 3936 |
| LY9 | LY9 | lymphocyte antigen 9 | 4063 |
| LYN | LYN | LYN proto-oncogene, Src family tyrosine kinase | 4067 |
| PIK3CD | PIK3CD | phosphatidylinositol-4,5-bisphosphate 3-kinase catalytic subunit delta | 5293 |
| PIK3CG | PIK3CG | phosphatidylinositol-4,5-bisphosphate 3-kinase catalytic subunit gamma | 5294 |
| PLEK | PLEK | pleckstrin | 5341 |
| SASH3 | SASH3 | SAM and SH3 domain containing 3 | 54440 |
| APBB1IP | APBB1IP | amyloid beta precursor protein binding family B member 1 interacting protein | 54518 |
| LAX1 | LAX1 | lymphocyte transmembrane adaptor 1 | 54900 |
| SIRPG | SIRPG | signal regulatory protein gamma | 55423 |
| PTGER4 | PTGER4 | prostaglandin E receptor 4 | 5734 |
| PTPN6 | PTPN6 | protein tyrosine phosphatase, non-receptor type 6 | 5777 |
| SLAMF7 | SLAMF7 | SLAM family member 7 | 57823 |
| RAC2 | RAC2 | ras-related C3 botulinum toxin substrate 2 (rho family, small GTP binding protein Rac2) | 5880 |
| CCL2 | CCL2 | C-C motif chemokine ligand 2 | 6347 |
| CCL5 | CCL5 | C-C motif chemokine ligand 5 | 6352 |
| SELL | SELL | selectin L | 6402 |
| PARVG | PARVG | parvin gamma | 64098 |
| SLAMF1 | SLAMF1 | signaling lymphocytic activation molecule family member 1 | 6504 |
| STAT1 | STAT1 | signal transducer and activator of transcription 1 | 6772 |
| ZFP36L2 | ZFP36L2 | ZFP36 ring finger protein like 2 | 678 |
| SYK | SYK | spleen associated tyrosine kinase | 6850 |
| CCR2 | CCR2 | C-C motif chemokine receptor 2 | 729230 |
| TNFRSF4 | TNFRSF4 | TNF receptor superfamily member 4 | 7293 |
| VAV1 | VAV1 | vav guanine nucleotide exchange factor 1 | 7409 |
| WAS | WAS | Wiskott-Aldrich syndrome | 7454 |
| ZAP70 | ZAP70 | zeta chain of T-cell receptor associated protein kinase 70 | 7535 |
| FERMT3 | FERMT3 | fermitin family member 3 | 83706 |
| HAVCR2 | HAVCR2 | hepatitis A virus cellular receptor 2 | 84868 |
| SKAP1 | SKAP1 | src kinase associated phosphoprotein 1 | 8631 |
| TNFRSF14 | TNFRSF14 | TNF receptor superfamily member 14 | 8764 |
| SIGLEC10 | SIGLEC10 | sialic acid binding Ig like lectin 10 | 89790 |
| PSTPIP1 | PSTPIP1 | proline-serine-threonine phosphatase interacting protein 1 | 9051 |
| CD2 | CD2 | CD2 molecule | 914 |
| CD3D | CD3D | CD3d molecule | 915 |
| RSAD2 | RSAD2 | radical S-adenosyl methionine domain containing 2 | 91543 |
| CD3E | CD3E | CD3e molecule | 916 |
| CD247 | CD247 | CD247 molecule | 919 |
| CD4 | CD4 | CD4 molecule | 920 |
| CD6 | CD6 | CD6 molecule | 923 |
| IL32 | IL32 | interleukin 32 | 9235 |
| CD7 | CD7 | CD7 molecule | 924 |
| CD8A | CD8A | CD8a molecule | 925 |
| CD83 | CD83 | CD83 molecule | 9308 |
| CD86 | CD86 | CD86 molecule | 942 |
| CD40LG | CD40LG | CD40 ligand | 959 |
| CD74 | CD74 | CD74 molecule | 972 |
| SPOCK2 | SPOCK2 | SPARC/osteonectin, cwcv and kazal like domains proteoglycan 2 | 9806 |
| TESPA1 | TESPA1 | thymocyte expressed, positive selection associated 1 | 9840 |

  
  

Download Table

| **ID:GO:0007159     &nbspName:leukocyte cell-cell adhesion** | | | |
| --- | --- | --- | --- |
| C=475; O=75; E=8.65; R=8.67; PValue=0e+00; FDR=0e+00 | | | |
| userid | Gene Symbol | Gene Name | Entrez Gene |
| RASGRP1 | RASGRP1 | RAS guanyl releasing protein 1 | 10125 |
| EBI3 | EBI3 | Epstein-Barr virus induced 3 | 10148 |
| SEMA4D | SEMA4D | semaphorin 4D | 10507 |
| TNFSF13B | TNFSF13B | tumor necrosis factor superfamily member 13b | 10673 |
| BTN3A1 | BTN3A1 | butyrophilin subfamily 3 member A1 | 11119 |
| CORO1A | CORO1A | coronin 1A | 11151 |
| NLRP3 | NLRP3 | NLR family pyrin domain containing 3 | 114548 |
| CCR7 | CCR7 | C-C motif chemokine receptor 7 | 1236 |
| DOCK2 | DOCK2 | dedicator of cytokinesis 2 | 1794 |
| GPR183 | GPR183 | G protein-coupled receptor 183 | 1880 |
| AIF1 | AIF1 | allograft inflammatory factor 1 | 199 |
| ETS1 | ETS1 | ETS proto-oncogene 1, transcription factor | 2113 |
| FYN | FYN | FYN proto-oncogene, Src family tyrosine kinase | 2534 |
| PTPN22 | PTPN22 | protein tyrosine phosphatase, non-receptor type 22 | 26191 |
| LAT | LAT | linker for activation of T-cells | 27040 |
| GPR18 | GPR18 | G protein-coupled receptor 18 | 2841 |
| NCKAP1L | NCKAP1L | NCK associated protein 1 like | 3071 |
| HLA-DMB | HLA-DMB | major histocompatibility complex, class II, DM beta | 3109 |
| HLA-DPA1 | HLA-DPA1 | major histocompatibility complex, class II, DP alpha 1 | 3113 |
| HLA-DPB1 | HLA-DPB1 | major histocompatibility complex, class II, DP beta 1 | 3115 |
| HLA-DRA | HLA-DRA | major histocompatibility complex, class II, DR alpha | 3122 |
| HLA-E | HLA-E | major histocompatibility complex, class I, E | 3133 |
| HLA-G | HLA-G | major histocompatibility complex, class I, G | 3135 |
| IFNG | IFNG | interferon gamma | 3458 |
| IL2RA | IL2RA | interleukin 2 receptor subunit alpha | 3559 |
| IL7R | IL7R | interleukin 7 receptor | 3575 |
| IDO1 | IDO1 | indoleamine 2,3-dioxygenase 1 | 3620 |
| IRF1 | IRF1 | interferon regulatory factor 1 | 3659 |
| IRF4 | IRF4 | interferon regulatory factor 4 | 3662 |
| ITGAL | ITGAL | integrin subunit alpha L | 3683 |
| ITGB2 | ITGB2 | integrin subunit beta 2 | 3689 |
| ITGB7 | ITGB7 | integrin subunit beta 7 | 3695 |
| ITK | ITK | IL2 inducible T-cell kinase | 3702 |
| THEMIS | THEMIS | thymocyte selection associated | 387357 |
| LAG3 | LAG3 | lymphocyte activating 3 | 3902 |
| LCK | LCK | LCK proto-oncogene, Src family tyrosine kinase | 3932 |
| LCP1 | LCP1 | lymphocyte cytosolic protein 1 | 3936 |
| LY9 | LY9 | lymphocyte antigen 9 | 4063 |
| LYN | LYN | LYN proto-oncogene, Src family tyrosine kinase | 4067 |
| PIK3CD | PIK3CD | phosphatidylinositol-4,5-bisphosphate 3-kinase catalytic subunit delta | 5293 |
| PIK3CG | PIK3CG | phosphatidylinositol-4,5-bisphosphate 3-kinase catalytic subunit gamma | 5294 |
| SASH3 | SASH3 | SAM and SH3 domain containing 3 | 54440 |
| APBB1IP | APBB1IP | amyloid beta precursor protein binding family B member 1 interacting protein | 54518 |
| LAX1 | LAX1 | lymphocyte transmembrane adaptor 1 | 54900 |
| SIRPG | SIRPG | signal regulatory protein gamma | 55423 |
| PTGER4 | PTGER4 | prostaglandin E receptor 4 | 5734 |
| PTPN6 | PTPN6 | protein tyrosine phosphatase, non-receptor type 6 | 5777 |
| RAC2 | RAC2 | ras-related C3 botulinum toxin substrate 2 (rho family, small GTP binding protein Rac2) | 5880 |
| CCL2 | CCL2 | C-C motif chemokine ligand 2 | 6347 |
| CCL5 | CCL5 | C-C motif chemokine ligand 5 | 6352 |
| SLAMF1 | SLAMF1 | signaling lymphocytic activation molecule family member 1 | 6504 |
| ZFP36L2 | ZFP36L2 | ZFP36 ring finger protein like 2 | 678 |
| SYK | SYK | spleen associated tyrosine kinase | 6850 |
| CCR2 | CCR2 | C-C motif chemokine receptor 2 | 729230 |
| TNFRSF4 | TNFRSF4 | TNF receptor superfamily member 4 | 7293 |
| VAV1 | VAV1 | vav guanine nucleotide exchange factor 1 | 7409 |
| WAS | WAS | Wiskott-Aldrich syndrome | 7454 |
| ZAP70 | ZAP70 | zeta chain of T-cell receptor associated protein kinase 70 | 7535 |
| FERMT3 | FERMT3 | fermitin family member 3 | 83706 |
| HAVCR2 | HAVCR2 | hepatitis A virus cellular receptor 2 | 84868 |
| TNFRSF14 | TNFRSF14 | TNF receptor superfamily member 14 | 8764 |
| CD2 | CD2 | CD2 molecule | 914 |
| CD3D | CD3D | CD3d molecule | 915 |
| RSAD2 | RSAD2 | radical S-adenosyl methionine domain containing 2 | 91543 |
| CD3E | CD3E | CD3e molecule | 916 |
| CD247 | CD247 | CD247 molecule | 919 |
| CD4 | CD4 | CD4 molecule | 920 |
| CD6 | CD6 | CD6 molecule | 923 |
| CD7 | CD7 | CD7 molecule | 924 |
| CD8A | CD8A | CD8a molecule | 925 |
| CD83 | CD83 | CD83 molecule | 9308 |
| CD86 | CD86 | CD86 molecule | 942 |
| CD40LG | CD40LG | CD40 ligand | 959 |
| CD74 | CD74 | CD74 molecule | 972 |
| TESPA1 | TESPA1 | thymocyte expressed, positive selection associated 1 | 9840 |

  
  

Download Table

| **ID:GO:0009605     &nbspName:response to external stimulus** | | | |
| --- | --- | --- | --- |
| C=1982; O=111; E=36.09; R=3.08; PValue=0e+00; FDR=0e+00 | | | |
| userid | Gene Symbol | Gene Name | Entrez Gene |
| CD96 | CD96 | CD96 molecule | 10225 |
| TRIM22 | TRIM22 | tripartite motif containing 22 | 10346 |
| IFITM3 | IFITM3 | interferon induced transmembrane protein 3 | 10410 |
| SEMA4D | SEMA4D | semaphorin 4D | 10507 |
| IFI44 | IFI44 | interferon induced protein 44 | 10561 |
| CXCL13 | CXCL13 | C-X-C motif chemokine ligand 13 | 10563 |
| GNLY | GNLY | granulysin | 10578 |
| IFITM2 | IFITM2 | interferon induced transmembrane protein 2 | 10581 |
| CXCR6 | CXCR6 | C-X-C motif chemokine receptor 6 | 10663 |
| IFI44L | IFI44L | interferon induced protein 44 like | 10964 |
| PIM2 | PIM2 | Pim-2 proto-oncogene, serine/threonine kinase | 11040 |
| CORO1A | CORO1A | coronin 1A | 11151 |
| NLRP3 | NLRP3 | NLR family pyrin domain containing 3 | 114548 |
| BATF2 | BATF2 | basic leucine zipper ATF-like transcription factor 2 | 116071 |
| TAGAP | TAGAP | T-cell activation RhoGTPase activating protein | 117289 |
| CCR1 | CCR1 | C-C motif chemokine receptor 1 | 1230 |
| CCR7 | CCR7 | C-C motif chemokine receptor 7 | 1236 |
| CSF1R | CSF1R | colony stimulating factor 1 receptor | 1436 |
| DOCK2 | DOCK2 | dedicator of cytokinesis 2 | 1794 |
| GPR183 | GPR183 | G protein-coupled receptor 183 | 1880 |
| S1PR1 | S1PR1 | sphingosine-1-phosphate receptor 1 | 1901 |
| AIF1 | AIF1 | allograft inflammatory factor 1 | 199 |
| ETS1 | ETS1 | ETS proto-oncogene 1, transcription factor | 2113 |
| ACSL4 | ACSL4 | acyl-CoA synthetase long-chain family member 4 | 2182 |
| SYT11 | SYT11 | synaptotagmin 11 | 23208 |
| FYN | FYN | FYN proto-oncogene, Src family tyrosine kinase | 2534 |
| PTPN22 | PTPN22 | protein tyrosine phosphatase, non-receptor type 22 | 26191 |
| GBP1 | GBP1 | guanylate binding protein 1 | 2633 |
| XCR1 | XCR1 | X-C motif chemokine receptor 1 | 2829 |
| CXCR3 | CXCR3 | C-X-C motif chemokine receptor 3 | 2833 |
| GPR18 | GPR18 | G protein-coupled receptor 18 | 2841 |
| HCK | HCK | HCK proto-oncogene, Src family tyrosine kinase | 3055 |
| NCKAP1L | NCKAP1L | NCK associated protein 1 like | 3071 |
| HLA-E | HLA-E | major histocompatibility complex, class I, E | 3133 |
| IRF8 | IRF8 | interferon regulatory factor 8 | 3394 |
| TMEM173 | TMEM173 | transmembrane protein 173 | 340061 |
| IFI16 | IFI16 | interferon gamma inducible protein 16 | 3428 |
| IFIT1 | IFIT1 | interferon induced protein with tetratricopeptide repeats 1 | 3434 |
| IFIT3 | IFIT3 | interferon induced protein with tetratricopeptide repeats 3 | 3437 |
| IFNG | IFNG | interferon gamma | 3458 |
| APOE | APOE | apolipoprotein E | 348 |
| IL2RA | IL2RA | interleukin 2 receptor subunit alpha | 3559 |
| FASLG | FASLG | Fas ligand | 356 |
| IL10RA | IL10RA | interleukin 10 receptor subunit alpha | 3587 |
| IDO1 | IDO1 | indoleamine 2,3-dioxygenase 1 | 3620 |
| CXCL10 | CXCL10 | C-X-C motif chemokine ligand 10 | 3627 |
| IRF1 | IRF1 | interferon regulatory factor 1 | 3659 |
| IRF4 | IRF4 | interferon regulatory factor 4 | 3662 |
| IRF7 | IRF7 | interferon regulatory factor 7 | 3665 |
| ITGB2 | ITGB2 | integrin subunit beta 2 | 3689 |
| LCK | LCK | LCK proto-oncogene, Src family tyrosine kinase | 3932 |
| LYN | LYN | LYN proto-oncogene, Src family tyrosine kinase | 4067 |
| LYZ | LYZ | lysozyme | 4069 |
| MEF2C | MEF2C | myocyte enhancer factor 2C | 4208 |
| CXCL9 | CXCL9 | C-X-C motif chemokine ligand 9 | 4283 |
| MPP1 | MPP1 | membrane palmitoylated protein 1 | 4354 |
| MX1 | MX1 | MX dynamin like GTPase 1 | 4599 |
| MX2 | MX2 | MX dynamin like GTPase 2 | 4600 |
| OAS1 | OAS1 | 2'-5'-oligoadenylate synthetase 1 | 4938 |
| OAS2 | OAS2 | 2'-5'-oligoadenylate synthetase 2 | 4939 |
| OAS3 | OAS3 | 2'-5'-oligoadenylate synthetase 3 | 4940 |
| HERC5 | HERC5 | HECT and RLD domain containing E3 ubiquitin protein ligase 5 | 51191 |
| TLR7 | TLR7 | toll like receptor 7 | 51284 |
| TLR8 | TLR8 | toll like receptor 8 | 51311 |
| PLAC8 | PLAC8 | placenta specific 8 | 51316 |
| ENPP2 | ENPP2 | ectonucleotide pyrophosphatase/phosphodiesterase 2 | 5168 |
| PIK3CD | PIK3CD | phosphatidylinositol-4,5-bisphosphate 3-kinase catalytic subunit delta | 5293 |
| PIK3CG | PIK3CG | phosphatidylinositol-4,5-bisphosphate 3-kinase catalytic subunit gamma | 5294 |
| PLCG2 | PLCG2 | phospholipase C gamma 2 | 5336 |
| PLEK | PLEK | pleckstrin | 5341 |
| PLSCR1 | PLSCR1 | phospholipid scramblase 1 | 5359 |
| PPP2R3C | PPP2R3C | protein phosphatase 2 regulatory subunit B''gamma | 55012 |
| PRF1 | PRF1 | perforin 1 | 5551 |
| DDX60 | DDX60 | DExD/H-box helicase 60 | 55601 |
| PTGER4 | PTGER4 | prostaglandin E receptor 4 | 5734 |
| RAC2 | RAC2 | ras-related C3 botulinum toxin substrate 2 (rho family, small GTP binding protein Rac2) | 5880 |
| CCL2 | CCL2 | C-C motif chemokine ligand 2 | 6347 |
| CCL5 | CCL5 | C-C motif chemokine ligand 5 | 6352 |
| GPSM3 | GPSM3 | G-protein signaling modulator 3 | 63940 |
| SLAMF1 | SLAMF1 | signaling lymphocytic activation molecule family member 1 | 6504 |
| STAT1 | STAT1 | signal transducer and activator of transcription 1 | 6772 |
| SYK | SYK | spleen associated tyrosine kinase | 6850 |
| BTK | BTK | Bruton tyrosine kinase | 695 |
| TLR4 | TLR4 | toll like receptor 4 | 7099 |
| SERPING1 | SERPING1 | serpin family G member 1 | 710 |
| TNFAIP3 | TNFAIP3 | TNF alpha induced protein 3 | 7128 |
| TNFRSF1B | TNFRSF1B | TNF receptor superfamily member 1B | 7133 |
| C2 | C2 | complement C2 | 717 |
| C3AR1 | C3AR1 | complement C3a receptor 1 | 719 |
| CCR2 | CCR2 | C-C motif chemokine receptor 2 | 729230 |
| TNFRSF4 | TNFRSF4 | TNF receptor superfamily member 4 | 7293 |
| VAV1 | VAV1 | vav guanine nucleotide exchange factor 1 | 7409 |
| WIPF1 | WIPF1 | WAS/WASL interacting protein family member 1 | 7456 |
| ZAP70 | ZAP70 | zeta chain of T-cell receptor associated protein kinase 70 | 7535 |
| HAVCR2 | HAVCR2 | hepatitis A virus cellular receptor 2 | 84868 |
| IFITM1 | IFITM1 | interferon induced transmembrane protein 1 | 8519 |
| SHANK3 | SHANK3 | SH3 and multiple ankyrin repeat domains 3 | 85358 |
| OASL | OASL | 2'-5'-oligoadenylate synthetase like | 8638 |
| TNFRSF14 | TNFRSF14 | TNF receptor superfamily member 14 | 8764 |
| AP1S2 | AP1S2 | adaptor related protein complex 1 sigma 2 subunit | 8905 |
| RSAD2 | RSAD2 | radical S-adenosyl methionine domain containing 2 | 91543 |
| CD3E | CD3E | CD3e molecule | 916 |
| CD247 | CD247 | CD247 molecule | 919 |
| CD4 | CD4 | CD4 molecule | 920 |
| CD6 | CD6 | CD6 molecule | 923 |
| CD8A | CD8A | CD8a molecule | 925 |
| CD14 | CD14 | CD14 molecule | 929 |
| CD86 | CD86 | CD86 molecule | 942 |
| AIM2 | AIM2 | absent in melanoma 2 | 9447 |
| ISG15 | ISG15 | ISG15 ubiquitin-like modifier | 9636 |
| CD74 | CD74 | CD74 molecule | 972 |

  
  

Download Table

| **ID:GO:0009607     &nbspName:response to biotic stimulus** | | | |
| --- | --- | --- | --- |
| C=809; O=78; E=14.73; R=5.3; PValue=0e+00; FDR=0e+00 | | | |
| userid | Gene Symbol | Gene Name | Entrez Gene |
| CD96 | CD96 | CD96 molecule | 10225 |
| TRIM22 | TRIM22 | tripartite motif containing 22 | 10346 |
| IFITM3 | IFITM3 | interferon induced transmembrane protein 3 | 10410 |
| IFI44 | IFI44 | interferon induced protein 44 | 10561 |
| CXCL13 | CXCL13 | C-X-C motif chemokine ligand 13 | 10563 |
| GNLY | GNLY | granulysin | 10578 |
| IFITM2 | IFITM2 | interferon induced transmembrane protein 2 | 10581 |
| IFI44L | IFI44L | interferon induced protein 44 like | 10964 |
| PIM2 | PIM2 | Pim-2 proto-oncogene, serine/threonine kinase | 11040 |
| NLRP3 | NLRP3 | NLR family pyrin domain containing 3 | 114548 |
| BATF2 | BATF2 | basic leucine zipper ATF-like transcription factor 2 | 116071 |
| TAGAP | TAGAP | T-cell activation RhoGTPase activating protein | 117289 |
| CCR7 | CCR7 | C-C motif chemokine receptor 7 | 1236 |
| DOCK2 | DOCK2 | dedicator of cytokinesis 2 | 1794 |
| SYT11 | SYT11 | synaptotagmin 11 | 23208 |
| FYN | FYN | FYN proto-oncogene, Src family tyrosine kinase | 2534 |
| PTPN22 | PTPN22 | protein tyrosine phosphatase, non-receptor type 22 | 26191 |
| GBP1 | GBP1 | guanylate binding protein 1 | 2633 |
| HCK | HCK | HCK proto-oncogene, Src family tyrosine kinase | 3055 |
| HLA-E | HLA-E | major histocompatibility complex, class I, E | 3133 |
| IRF8 | IRF8 | interferon regulatory factor 8 | 3394 |
| TMEM173 | TMEM173 | transmembrane protein 173 | 340061 |
| IFI16 | IFI16 | interferon gamma inducible protein 16 | 3428 |
| IFIT1 | IFIT1 | interferon induced protein with tetratricopeptide repeats 1 | 3434 |
| IFIT3 | IFIT3 | interferon induced protein with tetratricopeptide repeats 3 | 3437 |
| IFNG | IFNG | interferon gamma | 3458 |
| IL2RA | IL2RA | interleukin 2 receptor subunit alpha | 3559 |
| FASLG | FASLG | Fas ligand | 356 |
| IL10RA | IL10RA | interleukin 10 receptor subunit alpha | 3587 |
| IDO1 | IDO1 | indoleamine 2,3-dioxygenase 1 | 3620 |
| CXCL10 | CXCL10 | C-X-C motif chemokine ligand 10 | 3627 |
| IRF1 | IRF1 | interferon regulatory factor 1 | 3659 |
| IRF4 | IRF4 | interferon regulatory factor 4 | 3662 |
| IRF7 | IRF7 | interferon regulatory factor 7 | 3665 |
| LCK | LCK | LCK proto-oncogene, Src family tyrosine kinase | 3932 |
| LYN | LYN | LYN proto-oncogene, Src family tyrosine kinase | 4067 |
| LYZ | LYZ | lysozyme | 4069 |
| MEF2C | MEF2C | myocyte enhancer factor 2C | 4208 |
| CXCL9 | CXCL9 | C-X-C motif chemokine ligand 9 | 4283 |
| MX1 | MX1 | MX dynamin like GTPase 1 | 4599 |
| MX2 | MX2 | MX dynamin like GTPase 2 | 4600 |
| OAS1 | OAS1 | 2'-5'-oligoadenylate synthetase 1 | 4938 |
| OAS2 | OAS2 | 2'-5'-oligoadenylate synthetase 2 | 4939 |
| OAS3 | OAS3 | 2'-5'-oligoadenylate synthetase 3 | 4940 |
| HERC5 | HERC5 | HECT and RLD domain containing E3 ubiquitin protein ligase 5 | 51191 |
| TLR7 | TLR7 | toll like receptor 7 | 51284 |
| TLR8 | TLR8 | toll like receptor 8 | 51311 |
| PLAC8 | PLAC8 | placenta specific 8 | 51316 |
| PLCG2 | PLCG2 | phospholipase C gamma 2 | 5336 |
| PLSCR1 | PLSCR1 | phospholipid scramblase 1 | 5359 |
| PPP2R3C | PPP2R3C | protein phosphatase 2 regulatory subunit B''gamma | 55012 |
| PRF1 | PRF1 | perforin 1 | 5551 |
| DDX60 | DDX60 | DExD/H-box helicase 60 | 55601 |
| PTGER4 | PTGER4 | prostaglandin E receptor 4 | 5734 |
| CCL2 | CCL2 | C-C motif chemokine ligand 2 | 6347 |
| CCL5 | CCL5 | C-C motif chemokine ligand 5 | 6352 |
| STAT1 | STAT1 | signal transducer and activator of transcription 1 | 6772 |
| SYK | SYK | spleen associated tyrosine kinase | 6850 |
| BTK | BTK | Bruton tyrosine kinase | 695 |
| TLR4 | TLR4 | toll like receptor 4 | 7099 |
| TNFAIP3 | TNFAIP3 | TNF alpha induced protein 3 | 7128 |
| TNFRSF1B | TNFRSF1B | TNF receptor superfamily member 1B | 7133 |
| TNFRSF4 | TNFRSF4 | TNF receptor superfamily member 4 | 7293 |
| WIPF1 | WIPF1 | WAS/WASL interacting protein family member 1 | 7456 |
| HAVCR2 | HAVCR2 | hepatitis A virus cellular receptor 2 | 84868 |
| IFITM1 | IFITM1 | interferon induced transmembrane protein 1 | 8519 |
| OASL | OASL | 2'-5'-oligoadenylate synthetase like | 8638 |
| TNFRSF14 | TNFRSF14 | TNF receptor superfamily member 14 | 8764 |
| AP1S2 | AP1S2 | adaptor related protein complex 1 sigma 2 subunit | 8905 |
| RSAD2 | RSAD2 | radical S-adenosyl methionine domain containing 2 | 91543 |
| CD247 | CD247 | CD247 molecule | 919 |
| CD4 | CD4 | CD4 molecule | 920 |
| CD6 | CD6 | CD6 molecule | 923 |
| CD8A | CD8A | CD8a molecule | 925 |
| CD14 | CD14 | CD14 molecule | 929 |
| CD86 | CD86 | CD86 molecule | 942 |
| AIM2 | AIM2 | absent in melanoma 2 | 9447 |
| ISG15 | ISG15 | ISG15 ubiquitin-like modifier | 9636 |

  
  

Download Table

| **ID:GO:0009615     &nbspName:response to virus** | | | |
| --- | --- | --- | --- |
| C=304; O=48; E=5.53; R=8.67; PValue=0e+00; FDR=0e+00 | | | |
| userid | Gene Symbol | Gene Name | Entrez Gene |
| TRIM22 | TRIM22 | tripartite motif containing 22 | 10346 |
| IFITM3 | IFITM3 | interferon induced transmembrane protein 3 | 10410 |
| IFI44 | IFI44 | interferon induced protein 44 | 10561 |
| IFITM2 | IFITM2 | interferon induced transmembrane protein 2 | 10581 |
| IFI44L | IFI44L | interferon induced protein 44 like | 10964 |
| PIM2 | PIM2 | Pim-2 proto-oncogene, serine/threonine kinase | 11040 |
| NLRP3 | NLRP3 | NLR family pyrin domain containing 3 | 114548 |
| TAGAP | TAGAP | T-cell activation RhoGTPase activating protein | 117289 |
| DOCK2 | DOCK2 | dedicator of cytokinesis 2 | 1794 |
| FYN | FYN | FYN proto-oncogene, Src family tyrosine kinase | 2534 |
| GBP1 | GBP1 | guanylate binding protein 1 | 2633 |
| HCK | HCK | HCK proto-oncogene, Src family tyrosine kinase | 3055 |
| TMEM173 | TMEM173 | transmembrane protein 173 | 340061 |
| IFI16 | IFI16 | interferon gamma inducible protein 16 | 3428 |
| IFIT1 | IFIT1 | interferon induced protein with tetratricopeptide repeats 1 | 3434 |
| IFIT3 | IFIT3 | interferon induced protein with tetratricopeptide repeats 3 | 3437 |
| IFNG | IFNG | interferon gamma | 3458 |
| IL2RA | IL2RA | interleukin 2 receptor subunit alpha | 3559 |
| CXCL10 | CXCL10 | C-X-C motif chemokine ligand 10 | 3627 |
| IRF1 | IRF1 | interferon regulatory factor 1 | 3659 |
| IRF7 | IRF7 | interferon regulatory factor 7 | 3665 |
| LCK | LCK | LCK proto-oncogene, Src family tyrosine kinase | 3932 |
| MEF2C | MEF2C | myocyte enhancer factor 2C | 4208 |
| CXCL9 | CXCL9 | C-X-C motif chemokine ligand 9 | 4283 |
| MX1 | MX1 | MX dynamin like GTPase 1 | 4599 |
| MX2 | MX2 | MX dynamin like GTPase 2 | 4600 |
| OAS1 | OAS1 | 2'-5'-oligoadenylate synthetase 1 | 4938 |
| OAS2 | OAS2 | 2'-5'-oligoadenylate synthetase 2 | 4939 |
| OAS3 | OAS3 | 2'-5'-oligoadenylate synthetase 3 | 4940 |
| HERC5 | HERC5 | HECT and RLD domain containing E3 ubiquitin protein ligase 5 | 51191 |
| TLR7 | TLR7 | toll like receptor 7 | 51284 |
| TLR8 | TLR8 | toll like receptor 8 | 51311 |
| PLSCR1 | PLSCR1 | phospholipid scramblase 1 | 5359 |
| PRF1 | PRF1 | perforin 1 | 5551 |
| DDX60 | DDX60 | DExD/H-box helicase 60 | 55601 |
| CCL5 | CCL5 | C-C motif chemokine ligand 5 | 6352 |
| STAT1 | STAT1 | signal transducer and activator of transcription 1 | 6772 |
| TNFAIP3 | TNFAIP3 | TNF alpha induced protein 3 | 7128 |
| IFITM1 | IFITM1 | interferon induced transmembrane protein 1 | 8519 |
| OASL | OASL | 2'-5'-oligoadenylate synthetase like | 8638 |
| AP1S2 | AP1S2 | adaptor related protein complex 1 sigma 2 subunit | 8905 |
| RSAD2 | RSAD2 | radical S-adenosyl methionine domain containing 2 | 91543 |
| CD247 | CD247 | CD247 molecule | 919 |
| CD4 | CD4 | CD4 molecule | 920 |
| CD8A | CD8A | CD8a molecule | 925 |
| CD86 | CD86 | CD86 molecule | 942 |
| AIM2 | AIM2 | absent in melanoma 2 | 9447 |
| ISG15 | ISG15 | ISG15 ubiquitin-like modifier | 9636 |

  
  

Download Table

| **ID:GO:0016337     &nbspName:single organismal cell-cell adhesion** | | | |
| --- | --- | --- | --- |
| C=711; O=79; E=12.94; R=6.1; PValue=0e+00; FDR=0e+00 | | | |
| userid | Gene Symbol | Gene Name | Entrez Gene |
| RASGRP1 | RASGRP1 | RAS guanyl releasing protein 1 | 10125 |
| EBI3 | EBI3 | Epstein-Barr virus induced 3 | 10148 |
| SEMA4D | SEMA4D | semaphorin 4D | 10507 |
| CXCL13 | CXCL13 | C-X-C motif chemokine ligand 13 | 10563 |
| TNFSF13B | TNFSF13B | tumor necrosis factor superfamily member 13b | 10673 |
| BTN3A1 | BTN3A1 | butyrophilin subfamily 3 member A1 | 11119 |
| CORO1A | CORO1A | coronin 1A | 11151 |
| NLRP3 | NLRP3 | NLR family pyrin domain containing 3 | 114548 |
| CCR7 | CCR7 | C-C motif chemokine receptor 7 | 1236 |
| DOCK2 | DOCK2 | dedicator of cytokinesis 2 | 1794 |
| GPR183 | GPR183 | G protein-coupled receptor 183 | 1880 |
| AIF1 | AIF1 | allograft inflammatory factor 1 | 199 |
| ETS1 | ETS1 | ETS proto-oncogene 1, transcription factor | 2113 |
| FYN | FYN | FYN proto-oncogene, Src family tyrosine kinase | 2534 |
| PTPN22 | PTPN22 | protein tyrosine phosphatase, non-receptor type 22 | 26191 |
| LAT | LAT | linker for activation of T-cells | 27040 |
| GPR18 | GPR18 | G protein-coupled receptor 18 | 2841 |
| NCKAP1L | NCKAP1L | NCK associated protein 1 like | 3071 |
| HLA-DMB | HLA-DMB | major histocompatibility complex, class II, DM beta | 3109 |
| HLA-DPA1 | HLA-DPA1 | major histocompatibility complex, class II, DP alpha 1 | 3113 |
| HLA-DPB1 | HLA-DPB1 | major histocompatibility complex, class II, DP beta 1 | 3115 |
| HLA-DRA | HLA-DRA | major histocompatibility complex, class II, DR alpha | 3122 |
| HLA-E | HLA-E | major histocompatibility complex, class I, E | 3133 |
| HLA-G | HLA-G | major histocompatibility complex, class I, G | 3135 |
| ICAM2 | ICAM2 | intercellular adhesion molecule 2 | 3384 |
| IFNG | IFNG | interferon gamma | 3458 |
| IL2RA | IL2RA | interleukin 2 receptor subunit alpha | 3559 |
| IL7R | IL7R | interleukin 7 receptor | 3575 |
| IDO1 | IDO1 | indoleamine 2,3-dioxygenase 1 | 3620 |
| IRF1 | IRF1 | interferon regulatory factor 1 | 3659 |
| IRF4 | IRF4 | interferon regulatory factor 4 | 3662 |
| ITGAL | ITGAL | integrin subunit alpha L | 3683 |
| ITGAX | ITGAX | integrin subunit alpha X | 3687 |
| ITGB2 | ITGB2 | integrin subunit beta 2 | 3689 |
| ITGB7 | ITGB7 | integrin subunit beta 7 | 3695 |
| ITK | ITK | IL2 inducible T-cell kinase | 3702 |
| THEMIS | THEMIS | thymocyte selection associated | 387357 |
| LAG3 | LAG3 | lymphocyte activating 3 | 3902 |
| LCK | LCK | LCK proto-oncogene, Src family tyrosine kinase | 3932 |
| LCP1 | LCP1 | lymphocyte cytosolic protein 1 | 3936 |
| LY9 | LY9 | lymphocyte antigen 9 | 4063 |
| LYN | LYN | LYN proto-oncogene, Src family tyrosine kinase | 4067 |
| PIK3CD | PIK3CD | phosphatidylinositol-4,5-bisphosphate 3-kinase catalytic subunit delta | 5293 |
| PIK3CG | PIK3CG | phosphatidylinositol-4,5-bisphosphate 3-kinase catalytic subunit gamma | 5294 |
| PLEK | PLEK | pleckstrin | 5341 |
| SASH3 | SASH3 | SAM and SH3 domain containing 3 | 54440 |
| APBB1IP | APBB1IP | amyloid beta precursor protein binding family B member 1 interacting protein | 54518 |
| LAX1 | LAX1 | lymphocyte transmembrane adaptor 1 | 54900 |
| SIRPG | SIRPG | signal regulatory protein gamma | 55423 |
| PTGER4 | PTGER4 | prostaglandin E receptor 4 | 5734 |
| PTPN6 | PTPN6 | protein tyrosine phosphatase, non-receptor type 6 | 5777 |
| RAC2 | RAC2 | ras-related C3 botulinum toxin substrate 2 (rho family, small GTP binding protein Rac2) | 5880 |
| CCL2 | CCL2 | C-C motif chemokine ligand 2 | 6347 |
| CCL5 | CCL5 | C-C motif chemokine ligand 5 | 6352 |
| SLAMF1 | SLAMF1 | signaling lymphocytic activation molecule family member 1 | 6504 |
| ZFP36L2 | ZFP36L2 | ZFP36 ring finger protein like 2 | 678 |
| SYK | SYK | spleen associated tyrosine kinase | 6850 |
| CCR2 | CCR2 | C-C motif chemokine receptor 2 | 729230 |
| TNFRSF4 | TNFRSF4 | TNF receptor superfamily member 4 | 7293 |
| VAV1 | VAV1 | vav guanine nucleotide exchange factor 1 | 7409 |
| WAS | WAS | Wiskott-Aldrich syndrome | 7454 |
| ZAP70 | ZAP70 | zeta chain of T-cell receptor associated protein kinase 70 | 7535 |
| FERMT3 | FERMT3 | fermitin family member 3 | 83706 |
| HAVCR2 | HAVCR2 | hepatitis A virus cellular receptor 2 | 84868 |
| TNFRSF14 | TNFRSF14 | TNF receptor superfamily member 14 | 8764 |
| CD2 | CD2 | CD2 molecule | 914 |
| CD3D | CD3D | CD3d molecule | 915 |
| RSAD2 | RSAD2 | radical S-adenosyl methionine domain containing 2 | 91543 |
| CD3E | CD3E | CD3e molecule | 916 |
| CD247 | CD247 | CD247 molecule | 919 |
| CD4 | CD4 | CD4 molecule | 920 |
| CD6 | CD6 | CD6 molecule | 923 |
| CD7 | CD7 | CD7 molecule | 924 |
| CD8A | CD8A | CD8a molecule | 925 |
| CD83 | CD83 | CD83 molecule | 9308 |
| CD86 | CD86 | CD86 molecule | 942 |
| CD40LG | CD40LG | CD40 ligand | 959 |
| CD74 | CD74 | CD74 molecule | 972 |
| TESPA1 | TESPA1 | thymocyte expressed, positive selection associated 1 | 9840 |

  
  

Download Table

| **ID:GO:0019221     &nbspName:cytokine-mediated signaling pathway** | | | |
| --- | --- | --- | --- |
| C=537; O=70; E=9.78; R=7.16; PValue=0e+00; FDR=0e+00 | | | |
| userid | Gene Symbol | Gene Name | Entrez Gene |
| EBI3 | EBI3 | Epstein-Barr virus induced 3 | 10148 |
| TRIM22 | TRIM22 | tripartite motif containing 22 | 10346 |
| IFITM3 | IFITM3 | interferon induced transmembrane protein 3 | 10410 |
| CXCL13 | CXCL13 | C-X-C motif chemokine ligand 13 | 10563 |
| IFITM2 | IFITM2 | interferon induced transmembrane protein 2 | 10581 |
| CXCR6 | CXCR6 | C-X-C motif chemokine receptor 6 | 10663 |
| TNFSF13B | TNFSF13B | tumor necrosis factor superfamily member 13b | 10673 |
| CCR1 | CCR1 | C-C motif chemokine receptor 1 | 1230 |
| CCR7 | CCR7 | C-C motif chemokine receptor 7 | 1236 |
| CSF1R | CSF1R | colony stimulating factor 1 receptor | 1436 |
| TNFRSF13B | TNFRSF13B | TNF receptor superfamily member 13B | 23495 |
| GBP1 | GBP1 | guanylate binding protein 1 | 2633 |
| GBP2 | GBP2 | guanylate binding protein 2 | 2634 |
| XCR1 | XCR1 | X-C motif chemokine receptor 1 | 2829 |
| CXCR3 | CXCR3 | C-X-C motif chemokine receptor 3 | 2833 |
| HCK | HCK | HCK proto-oncogene, Src family tyrosine kinase | 3055 |
| HLA-DPA1 | HLA-DPA1 | major histocompatibility complex, class II, DP alpha 1 | 3113 |
| HLA-DPB1 | HLA-DPB1 | major histocompatibility complex, class II, DP beta 1 | 3115 |
| HLA-DRA | HLA-DRA | major histocompatibility complex, class II, DR alpha | 3122 |
| HLA-E | HLA-E | major histocompatibility complex, class I, E | 3133 |
| HLA-F | HLA-F | major histocompatibility complex, class I, F | 3134 |
| HLA-G | HLA-G | major histocompatibility complex, class I, G | 3135 |
| IRF8 | IRF8 | interferon regulatory factor 8 | 3394 |
| IFI27 | IFI27 | interferon alpha inducible protein 27 | 3429 |
| IFI35 | IFI35 | interferon induced protein 35 | 3430 |
| IFIT1 | IFIT1 | interferon induced protein with tetratricopeptide repeats 1 | 3434 |
| IFIT3 | IFIT3 | interferon induced protein with tetratricopeptide repeats 3 | 3437 |
| IFNG | IFNG | interferon gamma | 3458 |
| IL2RA | IL2RA | interleukin 2 receptor subunit alpha | 3559 |
| IL2RB | IL2RB | interleukin 2 receptor subunit beta | 3560 |
| IL2RG | IL2RG | interleukin 2 receptor subunit gamma | 3561 |
| IL7R | IL7R | interleukin 7 receptor | 3575 |
| IL10RA | IL10RA | interleukin 10 receptor subunit alpha | 3587 |
| CXCL10 | CXCL10 | C-X-C motif chemokine ligand 10 | 3627 |
| IRF1 | IRF1 | interferon regulatory factor 1 | 3659 |
| IRF4 | IRF4 | interferon regulatory factor 4 | 3662 |
| IRF7 | IRF7 | interferon regulatory factor 7 | 3665 |
| LTB | LTB | lymphotoxin beta | 4050 |
| CIITA | CIITA | class II major histocompatibility complex transactivator | 4261 |
| CXCL9 | CXCL9 | C-X-C motif chemokine ligand 9 | 4283 |
| MX1 | MX1 | MX dynamin like GTPase 1 | 4599 |
| MX2 | MX2 | MX dynamin like GTPase 2 | 4600 |
| OAS1 | OAS1 | 2'-5'-oligoadenylate synthetase 1 | 4938 |
| OAS2 | OAS2 | 2'-5'-oligoadenylate synthetase 2 | 4939 |
| OAS3 | OAS3 | 2'-5'-oligoadenylate synthetase 3 | 4940 |
| IL21R | IL21R | interleukin 21 receptor | 50615 |
| PSMB8 | PSMB8 | proteasome subunit beta 8 | 5696 |
| PSMB9 | PSMB9 | proteasome subunit beta 9 | 5698 |
| PSMB10 | PSMB10 | proteasome subunit beta 10 | 5699 |
| PTPN6 | PTPN6 | protein tyrosine phosphatase, non-receptor type 6 | 5777 |
| TNFRSF17 | TNFRSF17 | TNF receptor superfamily member 17 | 608 |
| CCL2 | CCL2 | C-C motif chemokine ligand 2 | 6347 |
| CCL5 | CCL5 | C-C motif chemokine ligand 5 | 6352 |
| STAT1 | STAT1 | signal transducer and activator of transcription 1 | 6772 |
| SYK | SYK | spleen associated tyrosine kinase | 6850 |
| TNFAIP3 | TNFAIP3 | TNF alpha induced protein 3 | 7128 |
| TNFRSF1B | TNFRSF1B | TNF receptor superfamily member 1B | 7133 |
| TRAF1 | TRAF1 | TNF receptor associated factor 1 | 7185 |
| CCR2 | CCR2 | C-C motif chemokine receptor 2 | 729230 |
| TNFRSF4 | TNFRSF4 | TNF receptor superfamily member 4 | 7293 |
| PLVAP | PLVAP | plasmalemma vesicle associated protein | 83483 |
| IFITM1 | IFITM1 | interferon induced transmembrane protein 1 | 8519 |
| OASL | OASL | 2'-5'-oligoadenylate synthetase like | 8638 |
| TNFSF13 | TNFSF13 | tumor necrosis factor superfamily member 13 | 8741 |
| TNFRSF14 | TNFRSF14 | TNF receptor superfamily member 14 | 8764 |
| RSAD2 | RSAD2 | radical S-adenosyl methionine domain containing 2 | 91543 |
| AIM2 | AIM2 | absent in melanoma 2 | 9447 |
| CD40LG | CD40LG | CD40 ligand | 959 |
| ISG15 | ISG15 | ISG15 ubiquitin-like modifier | 9636 |
| CD74 | CD74 | CD74 molecule | 972 |

  
  

Download Table

| **ID:GO:0022407     &nbspName:regulation of cell-cell adhesion** | | | |
| --- | --- | --- | --- |
| C=379; O=54; E=6.9; R=7.83; PValue=0e+00; FDR=0e+00 | | | |
| userid | Gene Symbol | Gene Name | Entrez Gene |
| RASGRP1 | RASGRP1 | RAS guanyl releasing protein 1 | 10125 |
| EBI3 | EBI3 | Epstein-Barr virus induced 3 | 10148 |
| CXCL13 | CXCL13 | C-X-C motif chemokine ligand 13 | 10563 |
| TNFSF13B | TNFSF13B | tumor necrosis factor superfamily member 13b | 10673 |
| CORO1A | CORO1A | coronin 1A | 11151 |
| NLRP3 | NLRP3 | NLR family pyrin domain containing 3 | 114548 |
| CCR7 | CCR7 | C-C motif chemokine receptor 7 | 1236 |
| AIF1 | AIF1 | allograft inflammatory factor 1 | 199 |
| ETS1 | ETS1 | ETS proto-oncogene 1, transcription factor | 2113 |
| FYN | FYN | FYN proto-oncogene, Src family tyrosine kinase | 2534 |
| PTPN22 | PTPN22 | protein tyrosine phosphatase, non-receptor type 22 | 26191 |
| LAT | LAT | linker for activation of T-cells | 27040 |
| NCKAP1L | NCKAP1L | NCK associated protein 1 like | 3071 |
| HLA-DMB | HLA-DMB | major histocompatibility complex, class II, DM beta | 3109 |
| HLA-DPA1 | HLA-DPA1 | major histocompatibility complex, class II, DP alpha 1 | 3113 |
| HLA-DPB1 | HLA-DPB1 | major histocompatibility complex, class II, DP beta 1 | 3115 |
| HLA-DRA | HLA-DRA | major histocompatibility complex, class II, DR alpha | 3122 |
| HLA-E | HLA-E | major histocompatibility complex, class I, E | 3133 |
| HLA-G | HLA-G | major histocompatibility complex, class I, G | 3135 |
| IFNG | IFNG | interferon gamma | 3458 |
| IL2RA | IL2RA | interleukin 2 receptor subunit alpha | 3559 |
| IL7R | IL7R | interleukin 7 receptor | 3575 |
| IDO1 | IDO1 | indoleamine 2,3-dioxygenase 1 | 3620 |
| IRF1 | IRF1 | interferon regulatory factor 1 | 3659 |
| IRF4 | IRF4 | interferon regulatory factor 4 | 3662 |
| LAG3 | LAG3 | lymphocyte activating 3 | 3902 |
| LCK | LCK | LCK proto-oncogene, Src family tyrosine kinase | 3932 |
| LYN | LYN | LYN proto-oncogene, Src family tyrosine kinase | 4067 |
| SASH3 | SASH3 | SAM and SH3 domain containing 3 | 54440 |
| LAX1 | LAX1 | lymphocyte transmembrane adaptor 1 | 54900 |
| SIRPG | SIRPG | signal regulatory protein gamma | 55423 |
| PTPN6 | PTPN6 | protein tyrosine phosphatase, non-receptor type 6 | 5777 |
| RAC2 | RAC2 | ras-related C3 botulinum toxin substrate 2 (rho family, small GTP binding protein Rac2) | 5880 |
| CCL2 | CCL2 | C-C motif chemokine ligand 2 | 6347 |
| CCL5 | CCL5 | C-C motif chemokine ligand 5 | 6352 |
| SLAMF1 | SLAMF1 | signaling lymphocytic activation molecule family member 1 | 6504 |
| SYK | SYK | spleen associated tyrosine kinase | 6850 |
| CCR2 | CCR2 | C-C motif chemokine receptor 2 | 729230 |
| VAV1 | VAV1 | vav guanine nucleotide exchange factor 1 | 7409 |
| ZAP70 | ZAP70 | zeta chain of T-cell receptor associated protein kinase 70 | 7535 |
| FERMT3 | FERMT3 | fermitin family member 3 | 83706 |
| HAVCR2 | HAVCR2 | hepatitis A virus cellular receptor 2 | 84868 |
| TNFRSF14 | TNFRSF14 | TNF receptor superfamily member 14 | 8764 |
| CD2 | CD2 | CD2 molecule | 914 |
| CD3D | CD3D | CD3d molecule | 915 |
| CD3E | CD3E | CD3e molecule | 916 |
| CD247 | CD247 | CD247 molecule | 919 |
| CD4 | CD4 | CD4 molecule | 920 |
| CD6 | CD6 | CD6 molecule | 923 |
| CD83 | CD83 | CD83 molecule | 9308 |
| CD86 | CD86 | CD86 molecule | 942 |
| CD40LG | CD40LG | CD40 ligand | 959 |
| CD74 | CD74 | CD74 molecule | 972 |
| TESPA1 | TESPA1 | thymocyte expressed, positive selection associated 1 | 9840 |

  
  

Download Table

| **ID:GO:0022409     &nbspName:positive regulation of cell-cell adhesion** | | | |
| --- | --- | --- | --- |
| C=238; O=44; E=4.33; R=10.15; PValue=0e+00; FDR=0e+00 | | | |
| userid | Gene Symbol | Gene Name | Entrez Gene |
| RASGRP1 | RASGRP1 | RAS guanyl releasing protein 1 | 10125 |
| EBI3 | EBI3 | Epstein-Barr virus induced 3 | 10148 |
| CXCL13 | CXCL13 | C-X-C motif chemokine ligand 13 | 10563 |
| TNFSF13B | TNFSF13B | tumor necrosis factor superfamily member 13b | 10673 |
| CORO1A | CORO1A | coronin 1A | 11151 |
| NLRP3 | NLRP3 | NLR family pyrin domain containing 3 | 114548 |
| CCR7 | CCR7 | C-C motif chemokine receptor 7 | 1236 |
| AIF1 | AIF1 | allograft inflammatory factor 1 | 199 |
| ETS1 | ETS1 | ETS proto-oncogene 1, transcription factor | 2113 |
| FYN | FYN | FYN proto-oncogene, Src family tyrosine kinase | 2534 |
| NCKAP1L | NCKAP1L | NCK associated protein 1 like | 3071 |
| HLA-DMB | HLA-DMB | major histocompatibility complex, class II, DM beta | 3109 |
| HLA-DPA1 | HLA-DPA1 | major histocompatibility complex, class II, DP alpha 1 | 3113 |
| HLA-DPB1 | HLA-DPB1 | major histocompatibility complex, class II, DP beta 1 | 3115 |
| HLA-DRA | HLA-DRA | major histocompatibility complex, class II, DR alpha | 3122 |
| HLA-E | HLA-E | major histocompatibility complex, class I, E | 3133 |
| HLA-G | HLA-G | major histocompatibility complex, class I, G | 3135 |
| IFNG | IFNG | interferon gamma | 3458 |
| IL2RA | IL2RA | interleukin 2 receptor subunit alpha | 3559 |
| IL7R | IL7R | interleukin 7 receptor | 3575 |
| LCK | LCK | LCK proto-oncogene, Src family tyrosine kinase | 3932 |
| LYN | LYN | LYN proto-oncogene, Src family tyrosine kinase | 4067 |
| SASH3 | SASH3 | SAM and SH3 domain containing 3 | 54440 |
| SIRPG | SIRPG | signal regulatory protein gamma | 55423 |
| PTPN6 | PTPN6 | protein tyrosine phosphatase, non-receptor type 6 | 5777 |
| CCL2 | CCL2 | C-C motif chemokine ligand 2 | 6347 |
| CCL5 | CCL5 | C-C motif chemokine ligand 5 | 6352 |
| SLAMF1 | SLAMF1 | signaling lymphocytic activation molecule family member 1 | 6504 |
| SYK | SYK | spleen associated tyrosine kinase | 6850 |
| CCR2 | CCR2 | C-C motif chemokine receptor 2 | 729230 |
| VAV1 | VAV1 | vav guanine nucleotide exchange factor 1 | 7409 |
| ZAP70 | ZAP70 | zeta chain of T-cell receptor associated protein kinase 70 | 7535 |
| HAVCR2 | HAVCR2 | hepatitis A virus cellular receptor 2 | 84868 |
| TNFRSF14 | TNFRSF14 | TNF receptor superfamily member 14 | 8764 |
| CD3D | CD3D | CD3d molecule | 915 |
| CD3E | CD3E | CD3e molecule | 916 |
| CD247 | CD247 | CD247 molecule | 919 |
| CD4 | CD4 | CD4 molecule | 920 |
| CD6 | CD6 | CD6 molecule | 923 |
| CD83 | CD83 | CD83 molecule | 9308 |
| CD86 | CD86 | CD86 molecule | 942 |
| CD40LG | CD40LG | CD40 ligand | 959 |
| CD74 | CD74 | CD74 molecule | 972 |
| TESPA1 | TESPA1 | thymocyte expressed, positive selection associated 1 | 9840 |

  
  

Download Table

| **ID:GO:0022610     &nbspName:biological adhesion** | | | |
| --- | --- | --- | --- |
| C=1673; O=98; E=30.46; R=3.22; PValue=0e+00; FDR=0e+00 | | | |
| userid | Gene Symbol | Gene Name | Entrez Gene |
| RASGRP1 | RASGRP1 | RAS guanyl releasing protein 1 | 10125 |
| EBI3 | EBI3 | Epstein-Barr virus induced 3 | 10148 |
| CD96 | CD96 | CD96 molecule | 10225 |
| SEMA4D | SEMA4D | semaphorin 4D | 10507 |
| CXCL13 | CXCL13 | C-X-C motif chemokine ligand 13 | 10563 |
| TNFSF13B | TNFSF13B | tumor necrosis factor superfamily member 13b | 10673 |
| BTN3A1 | BTN3A1 | butyrophilin subfamily 3 member A1 | 11119 |
| CORO1A | CORO1A | coronin 1A | 11151 |
| NLRP3 | NLRP3 | NLR family pyrin domain containing 3 | 114548 |
| CCR1 | CCR1 | C-C motif chemokine receptor 1 | 1230 |
| CCR7 | CCR7 | C-C motif chemokine receptor 7 | 1236 |
| SIRPA | SIRPA | signal regulatory protein alpha | 140885 |
| HAPLN3 | HAPLN3 | hyaluronan and proteoglycan link protein 3 | 145864 |
| DOCK2 | DOCK2 | dedicator of cytokinesis 2 | 1794 |
| GPR183 | GPR183 | G protein-coupled receptor 183 | 1880 |
| S1PR1 | S1PR1 | sphingosine-1-phosphate receptor 1 | 1901 |
| AIF1 | AIF1 | allograft inflammatory factor 1 | 199 |
| ETS1 | ETS1 | ETS proto-oncogene 1, transcription factor | 2113 |
| FYN | FYN | FYN proto-oncogene, Src family tyrosine kinase | 2534 |
| ABI3BP | ABI3BP | ABI family member 3 binding protein | 25890 |
| PTPN22 | PTPN22 | protein tyrosine phosphatase, non-receptor type 22 | 26191 |
| GBP1 | GBP1 | guanylate binding protein 1 | 2633 |
| LAT | LAT | linker for activation of T-cells | 27040 |
| ADAMDEC1 | ADAMDEC1 | ADAM like decysin 1 | 27299 |
| CXCR3 | CXCR3 | C-X-C motif chemokine receptor 3 | 2833 |
| GPR18 | GPR18 | G protein-coupled receptor 18 | 2841 |
| HCK | HCK | HCK proto-oncogene, Src family tyrosine kinase | 3055 |
| NCKAP1L | NCKAP1L | NCK associated protein 1 like | 3071 |
| HLA-DMB | HLA-DMB | major histocompatibility complex, class II, DM beta | 3109 |
| HLA-DPA1 | HLA-DPA1 | major histocompatibility complex, class II, DP alpha 1 | 3113 |
| HLA-DPB1 | HLA-DPB1 | major histocompatibility complex, class II, DP beta 1 | 3115 |
| HLA-DRA | HLA-DRA | major histocompatibility complex, class II, DR alpha | 3122 |
| HLA-E | HLA-E | major histocompatibility complex, class I, E | 3133 |
| HLA-G | HLA-G | major histocompatibility complex, class I, G | 3135 |
| ICAM2 | ICAM2 | intercellular adhesion molecule 2 | 3384 |
| IFNG | IFNG | interferon gamma | 3458 |
| IL2RA | IL2RA | interleukin 2 receptor subunit alpha | 3559 |
| IL7R | IL7R | interleukin 7 receptor | 3575 |
| IDO1 | IDO1 | indoleamine 2,3-dioxygenase 1 | 3620 |
| IRF1 | IRF1 | interferon regulatory factor 1 | 3659 |
| IRF4 | IRF4 | interferon regulatory factor 4 | 3662 |
| ITGAL | ITGAL | integrin subunit alpha L | 3683 |
| ITGAX | ITGAX | integrin subunit alpha X | 3687 |
| ITGB2 | ITGB2 | integrin subunit beta 2 | 3689 |
| ITGB7 | ITGB7 | integrin subunit beta 7 | 3695 |
| ITK | ITK | IL2 inducible T-cell kinase | 3702 |
| THEMIS | THEMIS | thymocyte selection associated | 387357 |
| LAG3 | LAG3 | lymphocyte activating 3 | 3902 |
| LCK | LCK | LCK proto-oncogene, Src family tyrosine kinase | 3932 |
| LCP1 | LCP1 | lymphocyte cytosolic protein 1 | 3936 |
| LY9 | LY9 | lymphocyte antigen 9 | 4063 |
| LYN | LYN | LYN proto-oncogene, Src family tyrosine kinase | 4067 |
| PIK3CD | PIK3CD | phosphatidylinositol-4,5-bisphosphate 3-kinase catalytic subunit delta | 5293 |
| PIK3CG | PIK3CG | phosphatidylinositol-4,5-bisphosphate 3-kinase catalytic subunit gamma | 5294 |
| PLEK | PLEK | pleckstrin | 5341 |
| SASH3 | SASH3 | SAM and SH3 domain containing 3 | 54440 |
| APBB1IP | APBB1IP | amyloid beta precursor protein binding family B member 1 interacting protein | 54518 |
| LAX1 | LAX1 | lymphocyte transmembrane adaptor 1 | 54900 |
| SIRPG | SIRPG | signal regulatory protein gamma | 55423 |
| PTGER4 | PTGER4 | prostaglandin E receptor 4 | 5734 |
| PTPN6 | PTPN6 | protein tyrosine phosphatase, non-receptor type 6 | 5777 |
| SLAMF7 | SLAMF7 | SLAM family member 7 | 57823 |
| RAC2 | RAC2 | ras-related C3 botulinum toxin substrate 2 (rho family, small GTP binding protein Rac2) | 5880 |
| CCL2 | CCL2 | C-C motif chemokine ligand 2 | 6347 |
| CCL5 | CCL5 | C-C motif chemokine ligand 5 | 6352 |
| SELL | SELL | selectin L | 6402 |
| PARVG | PARVG | parvin gamma | 64098 |
| SLAMF1 | SLAMF1 | signaling lymphocytic activation molecule family member 1 | 6504 |
| STAT1 | STAT1 | signal transducer and activator of transcription 1 | 6772 |
| ZFP36L2 | ZFP36L2 | ZFP36 ring finger protein like 2 | 678 |
| SYK | SYK | spleen associated tyrosine kinase | 6850 |
| CCR2 | CCR2 | C-C motif chemokine receptor 2 | 729230 |
| TNFRSF4 | TNFRSF4 | TNF receptor superfamily member 4 | 7293 |
| VAV1 | VAV1 | vav guanine nucleotide exchange factor 1 | 7409 |
| WAS | WAS | Wiskott-Aldrich syndrome | 7454 |
| ZAP70 | ZAP70 | zeta chain of T-cell receptor associated protein kinase 70 | 7535 |
| FERMT3 | FERMT3 | fermitin family member 3 | 83706 |
| HAVCR2 | HAVCR2 | hepatitis A virus cellular receptor 2 | 84868 |
| SKAP1 | SKAP1 | src kinase associated phosphoprotein 1 | 8631 |
| TNFRSF14 | TNFRSF14 | TNF receptor superfamily member 14 | 8764 |
| SIGLEC10 | SIGLEC10 | sialic acid binding Ig like lectin 10 | 89790 |
| PSTPIP1 | PSTPIP1 | proline-serine-threonine phosphatase interacting protein 1 | 9051 |
| CD2 | CD2 | CD2 molecule | 914 |
| CD3D | CD3D | CD3d molecule | 915 |
| RSAD2 | RSAD2 | radical S-adenosyl methionine domain containing 2 | 91543 |
| CD3E | CD3E | CD3e molecule | 916 |
| CD247 | CD247 | CD247 molecule | 919 |
| CD4 | CD4 | CD4 molecule | 920 |
| CD6 | CD6 | CD6 molecule | 923 |
| IL32 | IL32 | interleukin 32 | 9235 |
| CD7 | CD7 | CD7 molecule | 924 |
| CD8A | CD8A | CD8a molecule | 925 |
| CD83 | CD83 | CD83 molecule | 9308 |
| CD86 | CD86 | CD86 molecule | 942 |
| CD40LG | CD40LG | CD40 ligand | 959 |
| CD74 | CD74 | CD74 molecule | 972 |
| SPOCK2 | SPOCK2 | SPARC/osteonectin, cwcv and kazal like domains proteoglycan 2 | 9806 |
| TESPA1 | TESPA1 | thymocyte expressed, positive selection associated 1 | 9840 |

  
  

Download Table

| **ID:GO:0030097     &nbspName:hemopoiesis** | | | |
| --- | --- | --- | --- |
| C=701; O=66; E=12.76; R=5.17; PValue=0e+00; FDR=0e+00 | | | |
| userid | Gene Symbol | Gene Name | Entrez Gene |
| RASGRP1 | RASGRP1 | RAS guanyl releasing protein 1 | 10125 |
| IKZF1 | IKZF1 | IKAROS family zinc finger 1 | 10320 |
| LILRB4 | LILRB4 | leukocyte immunoglobulin like receptor B4 | 11006 |
| NLRP3 | NLRP3 | NLR family pyrin domain containing 3 | 114548 |
| BATF2 | BATF2 | basic leucine zipper ATF-like transcription factor 2 | 116071 |
| CCR1 | CCR1 | C-C motif chemokine receptor 1 | 1230 |
| CCR7 | CCR7 | C-C motif chemokine receptor 7 | 1236 |
| CSF1R | CSF1R | colony stimulating factor 1 receptor | 1436 |
| DOCK2 | DOCK2 | dedicator of cytokinesis 2 | 1794 |
| GPR183 | GPR183 | G protein-coupled receptor 183 | 1880 |
| ETS1 | ETS1 | ETS proto-oncogene 1, transcription factor | 2113 |
| PLCL2 | PLCL2 | phospholipase C like 2 | 23228 |
| FLT3LG | FLT3LG | fms related tyrosine kinase 3 ligand | 2323 |
| TNFRSF13B | TNFRSF13B | TNF receptor superfamily member 13B | 23495 |
| PTPN22 | PTPN22 | protein tyrosine phosphatase, non-receptor type 22 | 26191 |
| GPR18 | GPR18 | G protein-coupled receptor 18 | 2841 |
| HCLS1 | HCLS1 | hematopoietic cell-specific Lyn substrate 1 | 3059 |
| NCKAP1L | NCKAP1L | NCK associated protein 1 like | 3071 |
| HLA-G | HLA-G | major histocompatibility complex, class I, G | 3135 |
| IRF8 | IRF8 | interferon regulatory factor 8 | 3394 |
| IFI16 | IFI16 | interferon gamma inducible protein 16 | 3428 |
| IFNG | IFNG | interferon gamma | 3458 |
| IL2RA | IL2RA | interleukin 2 receptor subunit alpha | 3559 |
| IL7R | IL7R | interleukin 7 receptor | 3575 |
| IRF1 | IRF1 | interferon regulatory factor 1 | 3659 |
| IRF4 | IRF4 | interferon regulatory factor 4 | 3662 |
| IRF7 | IRF7 | interferon regulatory factor 7 | 3665 |
| ITK | ITK | IL2 inducible T-cell kinase | 3702 |
| THEMIS | THEMIS | thymocyte selection associated | 387357 |
| LCK | LCK | LCK proto-oncogene, Src family tyrosine kinase | 3932 |
| LY9 | LY9 | lymphocyte antigen 9 | 4063 |
| LYN | LYN | LYN proto-oncogene, Src family tyrosine kinase | 4067 |
| MEF2C | MEF2C | myocyte enhancer factor 2C | 4208 |
| MFNG | MFNG | MFNG O-fucosylpeptide 3-beta-N-acetylglucosaminyltransferase | 4242 |
| PIK3CD | PIK3CD | phosphatidylinositol-4,5-bisphosphate 3-kinase catalytic subunit delta | 5293 |
| PIP4K2A | PIP4K2A | phosphatidylinositol-5-phosphate 4-kinase type 2 alpha | 5305 |
| PLCG2 | PLCG2 | phospholipase C gamma 2 | 5336 |
| PLEK | PLEK | pleckstrin | 5341 |
| SASH3 | SASH3 | SAM and SH3 domain containing 3 | 54440 |
| HERC6 | HERC6 | HECT and RLD domain containing E3 ubiquitin protein ligase family member 6 | 55008 |
| PPP2R3C | PPP2R3C | protein phosphatase 2 regulatory subunit B''gamma | 55012 |
| TMEM176A | TMEM176A | transmembrane protein 176A | 55365 |
| PTGER4 | PTGER4 | prostaglandin E receptor 4 | 5734 |
| PTPN6 | PTPN6 | protein tyrosine phosphatase, non-receptor type 6 | 5777 |
| SLAMF1 | SLAMF1 | signaling lymphocytic activation molecule family member 1 | 6504 |
| SPI1 | SPI1 | Spi-1 proto-oncogene | 6688 |
| ZFP36L2 | ZFP36L2 | ZFP36 ring finger protein like 2 | 678 |
| SYK | SYK | spleen associated tyrosine kinase | 6850 |
| BTK | BTK | Bruton tyrosine kinase | 695 |
| TLR4 | TLR4 | toll like receptor 4 | 7099 |
| TYROBP | TYROBP | TYRO protein tyrosine kinase binding protein | 7305 |
| VAV1 | VAV1 | vav guanine nucleotide exchange factor 1 | 7409 |
| ZAP70 | ZAP70 | zeta chain of T-cell receptor associated protein kinase 70 | 7535 |
| CD2 | CD2 | CD2 molecule | 914 |
| CD3D | CD3D | CD3d molecule | 915 |
| RSAD2 | RSAD2 | radical S-adenosyl methionine domain containing 2 | 91543 |
| CD3E | CD3E | CD3e molecule | 916 |
| CD4 | CD4 | CD4 molecule | 920 |
| CD8A | CD8A | CD8a molecule | 925 |
| CD83 | CD83 | CD83 molecule | 9308 |
| CD86 | CD86 | CD86 molecule | 942 |
| CD40LG | CD40LG | CD40 ligand | 959 |
| ISG15 | ISG15 | ISG15 ubiquitin-like modifier | 9636 |
| CD74 | CD74 | CD74 molecule | 972 |
| CD79B | CD79B | CD79b molecule | 974 |
| TESPA1 | TESPA1 | thymocyte expressed, positive selection associated 1 | 9840 |

  
  

Download Table

| **ID:GO:0030098     &nbspName:lymphocyte differentiation** | | | |
| --- | --- | --- | --- |
| C=305; O=47; E=5.55; R=8.46; PValue=0e+00; FDR=0e+00 | | | |
| userid | Gene Symbol | Gene Name | Entrez Gene |
| RASGRP1 | RASGRP1 | RAS guanyl releasing protein 1 | 10125 |
| IKZF1 | IKZF1 | IKAROS family zinc finger 1 | 10320 |
| NLRP3 | NLRP3 | NLR family pyrin domain containing 3 | 114548 |
| CCR7 | CCR7 | C-C motif chemokine receptor 7 | 1236 |
| DOCK2 | DOCK2 | dedicator of cytokinesis 2 | 1794 |
| GPR183 | GPR183 | G protein-coupled receptor 183 | 1880 |
| PLCL2 | PLCL2 | phospholipase C like 2 | 23228 |
| FLT3LG | FLT3LG | fms related tyrosine kinase 3 ligand | 2323 |
| PTPN22 | PTPN22 | protein tyrosine phosphatase, non-receptor type 22 | 26191 |
| GPR18 | GPR18 | G protein-coupled receptor 18 | 2841 |
| NCKAP1L | NCKAP1L | NCK associated protein 1 like | 3071 |
| HLA-G | HLA-G | major histocompatibility complex, class I, G | 3135 |
| IFNG | IFNG | interferon gamma | 3458 |
| IL2RA | IL2RA | interleukin 2 receptor subunit alpha | 3559 |
| IL7R | IL7R | interleukin 7 receptor | 3575 |
| IRF1 | IRF1 | interferon regulatory factor 1 | 3659 |
| IRF4 | IRF4 | interferon regulatory factor 4 | 3662 |
| ITK | ITK | IL2 inducible T-cell kinase | 3702 |
| THEMIS | THEMIS | thymocyte selection associated | 387357 |
| LCK | LCK | LCK proto-oncogene, Src family tyrosine kinase | 3932 |
| LY9 | LY9 | lymphocyte antigen 9 | 4063 |
| MFNG | MFNG | MFNG O-fucosylpeptide 3-beta-N-acetylglucosaminyltransferase | 4242 |
| PIK3CD | PIK3CD | phosphatidylinositol-4,5-bisphosphate 3-kinase catalytic subunit delta | 5293 |
| PLCG2 | PLCG2 | phospholipase C gamma 2 | 5336 |
| SASH3 | SASH3 | SAM and SH3 domain containing 3 | 54440 |
| PPP2R3C | PPP2R3C | protein phosphatase 2 regulatory subunit B''gamma | 55012 |
| PTGER4 | PTGER4 | prostaglandin E receptor 4 | 5734 |
| PTPN6 | PTPN6 | protein tyrosine phosphatase, non-receptor type 6 | 5777 |
| SLAMF1 | SLAMF1 | signaling lymphocytic activation molecule family member 1 | 6504 |
| SPI1 | SPI1 | Spi-1 proto-oncogene | 6688 |
| ZFP36L2 | ZFP36L2 | ZFP36 ring finger protein like 2 | 678 |
| SYK | SYK | spleen associated tyrosine kinase | 6850 |
| BTK | BTK | Bruton tyrosine kinase | 695 |
| VAV1 | VAV1 | vav guanine nucleotide exchange factor 1 | 7409 |
| ZAP70 | ZAP70 | zeta chain of T-cell receptor associated protein kinase 70 | 7535 |
| CD2 | CD2 | CD2 molecule | 914 |
| CD3D | CD3D | CD3d molecule | 915 |
| RSAD2 | RSAD2 | radical S-adenosyl methionine domain containing 2 | 91543 |
| CD3E | CD3E | CD3e molecule | 916 |
| CD4 | CD4 | CD4 molecule | 920 |
| CD8A | CD8A | CD8a molecule | 925 |
| CD83 | CD83 | CD83 molecule | 9308 |
| CD86 | CD86 | CD86 molecule | 942 |
| CD40LG | CD40LG | CD40 ligand | 959 |
| CD74 | CD74 | CD74 molecule | 972 |
| CD79B | CD79B | CD79b molecule | 974 |
| TESPA1 | TESPA1 | thymocyte expressed, positive selection associated 1 | 9840 |
